# Nutrition and nurture in infancy and childhood: Bio‐Cultural perspectives A 3 day conference

**DOI:** 10.1111/mcn.13579

**Published:** 2024-01-03

**Organised by the Maternal, Paternal and Infant Nutrition and Nurture Unit (MAINN)**

**University of Central Lancashire, Preston, UK**

**Wednesday 19 – Friday 21 April 2023**

**Grange Over Sands, Cumbria**

**Collated Abstracts**

**Keynote Speakers**

## Exploring the psychological impact of donor human milk and milk donation for families and communities

**Amy Brown**

Professor Maternal and Child Health

Swansea University

We know that donor human milk (DHM) can protect the health and development of premature babies. Likewise, we know that being able to meet infant feeding goals can be important for maternal mental health. But can receiving DHM for a baby support parental wellbeing? And how might being able to donate milk, or not, play a role? This talk brings together findings from three new research studies conducted in collaboration with the Human Milk Foundation to highlight the ways in which receiving and donating milk can impact upon wellbeing.

Study one explored parents’ experiences of receiving DHM for their baby in neonatal care or in the community via an open‐ended question online survey. It identified how receiving DHM could help ease maternal guilt at not being able to breastfeed, supported personal feeding plans and helped parents to feel supported and cared for at a difficult time. For those who were able to breastfeed, DHM often supported mothers to continue to build their own supply, with some going on to become a donor in the future. Fathers talked about how it helped them play a role at a difficult time, either organising donor milk for their baby or helping support their partner and baby.

Study two used a pre and post design to measure parental anxiety and depression before and after receiving donor milk for maternal or infant health reasons, finding that both significantly decreased after receiving milk. Although there are several explanations for this, parents attributed the care and kindness that they received and the relief at being able to give their baby donor milk to the decreases in their symptoms of anxiety and depression.

Finally, study three explored mothers’ experiences of being able to donate their milk or not. It showed how being able to donate milk supported wellbeing by increasing feelings of ‘giving back’, being part of a community and playing a role in helping others, often at a time when mothers felt vulnerable. This was especially important for mothers whose own baby had been in neonatal care, who were bereaved or experiencing postnatal depression. When mothers were unable to donate, often due to logistics or health factors, this could leave them feeling let down or abandoned, with their milk often going to waste.

Together the findings have clear implications for supporting donor milk use and prioritisation, whilst also adding to our understanding of the complex interplay between infant feeding and wellbeing.

## Preventing maternal and child malnutrition: lessons learned from evaluating 10 years of multi‐sectoral interventions in Nepal

**Kenda Cunningham**

Sr. Research Advisor

Helen Keller International

Between 2011 and 2022, stunting in Nepal declined more than 16 percentage points, but the variation by geography, wealth and other factors remains. Quality diets remain a struggle for many as do other underlying determinants for maternal and child nutrition. USAID has invested during this time to support the Government of Nepal's Multi‐sectoral Nutrition Plan through an at‐scale program known as Suaahara. Aiming to reduce maternal and child undernutrition, Suaahara implemented social and behavior change activities as well as those to build capacity of frontline workers and government across sectors and levels. Nutrition‐specific activities as well as health, family planning, agriculture, water and sanitation, governance, and gender equality and social inclusion activities were implemented between 2012 and 2022 in about 60% of the country. Recent mixed‐methods rigorous evaluations conducted by external researchers have shown the effect of these interventions including reductions in maternal underweight, improvements in maternal dietary diversity and all complementary feeding indicators; improvements in service availability at local health facilities and capacity of facility and community health workers; and marked improvements in municipal government awareness of nutrition, their roles and responsibilities in implementing nutrition plans and programs, and allocation of funds for nutrition. These successes lay the groundwork for Nepal's future investments in nutrition, including its upcoming third phase of its Multi‐Sectoral Nutrition Plan, which USAID also plans to support.

## Multi‐disciplinary efforts to support breastfeeding in the healthcare system – successes, challenges and lessons learned

Nathan C. Nickel

Director, Manitoba Centre for Health Policy

Associate Professor of Community Health Sciences

Manitoba Centre for Health Policy

University of Manitoba

Despite the evidence documenting the maternal‐child health, population health, and cost benefits of the Baby Friendly Hospital Initiative, the number of designated Baby Friendly facilities in many jurisdictions lag behind targets. This presentation will present an overview of multi‐disciplinary, collaborative, quality improvement initiatives designed to support facilities along their journey towards becoming Baby Friendly. Dr. Nickel will present an overview of quality improvement change initiatives focused on the complex changes necessary to achieve Baby Friendly designation. He will share details about the process that the Breastfeeding Committee of Canada used to create a pan‐Canadian, multi‐jurisdictional collaborative focused on Baby Friendly. He will describe the quality improvement methods and framework used by throughout the collaborative to support Baby Friendly. And, Dr. Nickel will highlight some of the key outcomes and lessons learned that emerged from this work.

## Learning from the past, looking to the future: lessons from 40 years in the field of breastfeeding

Professor Mary Renfrew

Professor Emerita

University of Dundee

Breastfeeding in the UK and globally is facing extraordinary challenges. Adverse societal norms, misleading marketing of infant formula, and a contentious media discourse create often insurmountable barriers for women and for those who support them. Despite advances in knowledge and positive developments in health policy and practice, factors including workforce shortages, enduring inappropriate healthcare practices, and inadequate health professional education undermine efforts to provide quality care for all women and babies. At the same time, food security challenges resulting from the combined pressures of the Covid 19 pandemic, global economic and political challenges, and climate change are starting to have an adverse impact on infant formula supplies. This presentation will draw on more than 40 years of work in breastfeeding to consider possible solutions and to propose future research priorities.

## Using the Baby Friendly Initiative to create systemic change on a national level – The UNICEF UK neonatal programme

Anne Woods

Deputy Director UNICEF UK Baby Friendly Initiative

UNICEF UK

Based on the UN Convention on the Rights of the Child (CRC), the Baby Friendly Initiative is a global programme of UNICEF and the World Health Organization and aims to improve practice for infant feeding in health care settings. The Baby Friendly Initiative was introduced into the UK in 1994 and has been very successful in changing routine care for babies, their mothers and families in hospital and community settings. There are currently 99% of maternity services and 92% of health visiting services working towards Baby Friendly accreditation. In addition, in universities, there are 76% of Midwifery programmes and 32% of Health Visiting programmes working towards the award (January 2023).

In 2012, the UK Baby Friendly Initiative undertook a major review which resulted in all the standards being expanded to cover holistic care for all babies, their parents, and families and for the first time, a decision was made to introduce bespoke standards for neonatal units. It was felt these expanded set of evidence‐based standards which encompassed care related to building close and loving relationships, infant feeding and supporting parents to be partners in care could have the potential to transform neonatal units and greatly improve outcomes for the most vulnerable babies. In the UK, around 1:7 sick or premature babies are admitted to a neonatal unit each year.

The Baby Friendly neonatal standards were launched in 2015 and since then support for implementation of the standards at a local, regional, and national level has been overwhelmingly positive. 125 out of 179 neonatal units (84%) are currently working to implement the standards, with 17 units having achieved full Baby Friendly UK neonatal accreditation.

This presentation will share the learning from nearly three decades of implementing the UNICEF UK Baby Friendly Initiative with a focus on the neonatal standards. The presentation will also include reflections on the rising and continued impact of the Commercial Milk Formula (CMF) industry, the current state of UK maternity services and what this may mean for babies, their parents and families in the future

### Oral Presentations

 

## Implementing a co‐parenting breastfeeding eHealth resource in multiple health care organizations throughout a health region to increase breastfeeding health literacy

Jennifer Abbass‐Dick

Ontario Tech University, Oshawa, Canada

**Background**: Breastfeeding is recommended by all leading health authorities due to its importance to the health of women and their infants. Nevertheless, breastfeeding rates remain suboptimal putting the health of women and their children at risk (Victora et al., 2016). The Baby Friendly Hospital Initiative, Ten Steps, clearly call for health care organizations to educate families on the importance, establishment and maintenance of breastfeeding and how to overcome common challenges (WHO, 2018); however, families report receiving inadequate, impractical information filled with conflicting messages (Beggs et al., 2021; Sihota et al., 2019). Implementing an evidence informed eHealth resource (Abbass‐Dick et al., 2020) in clinical interactions with health care providers in both the health department and hospitals throughout a health region will address this longstanding clinical issue. eHealth is increasingly being used and has been found to be effective in increasing breastfeeding outcomes. The implementation of this eHealth resource throughout a health region will increase opportunities for breastfeeding support across the perinatal period, consistent messaging and families access to credible and comprehensive breastfeeding information.

**Methods**: This study will inform health care providers about the eHealth resource implementation within programs and determine their specific needs. Quantitative data, collected via an online questionnaire informed by the Consolidated Framework for Implementation Research (CFIR) will explore a wide range of contextual factors and will look systematically at barriers and facilitators (Damschroder et al., 2009). Factors which will be explored include those related to the intervention itself; the inner context (hospital and health unit); the outer context (outside the organizations); the health care professionals; and the implementation process itself.

**Findings**: The findings will be used to inform future implementation activities, tailoring them to the local context.

**Conclusion**: The provision of an eHealth resource alone is not sufficient to increase breastfeeding rates and health literacy, as the effective implementation in clinical practice is required. This study will inform effective implementation of the eHealth resource in clinical interactions spanning the childbearing period through multiple organizations in a health region to increase health literacy and breastfeeding rates.

## A narrative inquiry approach to understand how women from different socioeconomic backgrounds experience the revised UNICEF UK Baby Friendly Initiative (BFI) standards

Pippa Atkinson, Nicola Crossland, Gill Thomson and Anna Byrom

University of Central Lancashire, Preston, United Kingdom

**Background**: The UNICEF UK BFI revised standards emphasise the importance of supporting and valuing the mother‐baby relationship and aim to promote safe and informed infant feeding behaviours irrespective of infant feeding method (UNICEF UK BFI, 2013). Currently, there is no research relating to how women experience the revised BFI standards over time and whether the socioeconomic context influences the care she receives.

**Methods**: This longitudinal qualitative study employed narrative research methodology (Riessman, 2008), to gain an understanding of women's infant feeding care experiences. As this research focused on the women's storytelling, experience‐centred narrative provided a useful approach to understand the meaning of the stories (Squire, 2013). This study was undertaken in an area in the Northwest of England where all services (maternity, health visiting and Children's Centres) providing infant feeding care have embedded the BFI standards and been awarded BFI Gold accreditation. Research ethics permission was obtained from the NHS Health Research Authority and Health and Care Research Wales. Women from high and low socioeconomic backgrounds were recruited.

Diaries and interviews were used to collect stories from women pregnant with their first baby, at specific points antenatal until around 6 months after birth. Women were prompted to complete an entry in their diary after an appointment with a health professional who had provided them with information about feeding and caring for their baby. Diary entries were completed within a OneNote notebook that provided various options for recording thoughts and feelings. Interviews were undertaken to further explore the stories recorded in the diary entries. Reflexive activities were used throughout the study to consider assumptions and biases. After a full record of each participant's story had been obtained, re‐storying identified sequence, content, and consequence for each story. Content analysis (Riessman, 1993) was then used to identify the multi‐system level factors that influenced the women's infant feeding experiences.

**Findings**: Eight women participated in the study. Half were from a higher socio‐economic background and half from a lower socio‐economic background. Six themes were identified, and case‐studies created to illustrate the interactions between the multi‐system level factors. Similarities and differences between the case‐studies which related to the women's socio‐economic background were then explored to answer the research questions.

**Conclusion**: Findings from this study could be used by organisations when embedding the standards to improve care and reduce any inequalities mothers and babies experience when accessing infant feeding care.

**References**

Riessman, C.K. (1993). *Narrative analysis*. SAGE.

Squire, C. (2013). From experience‐centred to socioculturally‐orientated approaches to narrative. In M. Andrews., C. Squire & M. Tamboukou (Eds.), *Doing Narrative Research* (pp. 47–71) SAGE.

UNICEF UK Baby Friendly Initiative (2013) *The evidence and rationale for the UNICEF UK Baby Friendly Initiative Standards*. Retrieved 25 September 2023, from https://www.unicef.org.uk/babyfriendly/about/evidence-and-rationale-for-the-baby-friendly-standards

## Parental Socioeconomic Status and Peer Group Influence on Children's Nutrition Awareness and Food Choice in Ibadan, Nigeria

Akindele Abimibayo Adeoya^1,2*^, Adewale Olugbemiga Adeleye^3^, Adetoun Tayewo Akinwusi^4^, Ryoichi Nagatomi^1,5^

^1^Medicine and Science in Sports and Exercise Laboratory, Tohoku University Graduate School of Medicine, Sendai, Miyagi Prefecture, Japan. ^2^Division for Interdisciplinary Advanced Research and Education, Tohoku University Advanced Graduate School, Sendai, Miyagi Prefecture, Japan. ^3^Department of Human Kinetics and Health Education, Faculty of Education, Olabisi Onabanjo University, Ogun State, Nigeria. ^4^Department of Human Kinetics and Health Education, Faculty of Education, University of Ibadan, Oyo State, Nigeria. ^5^Division of Biomedical Engineering for Health and Welfare, Graduate School of Biomedical Engineering, Tohoku University, Sendai, Miyagi Prefecture, Japan

**Background**: Nutritional food choices are a challenge among students at public primary schools in Nigeria, most of whom are from low socioeconomic backgrounds. Most children imitate their peers, especially in selecting food items in school, leading to the selection of a poor nutritious diet. This study investigated parental socioeconomic status and peer group influence (PGI) on children's nutrition awareness and food choices in Ibadan, Nigeria.

**Methods**: This pre‐test/posttest quasi‐experimental study was conducted with 100 fourth‐ and fifth‐grade students in a public elementary school in Ibadan. A multi‐stage sampling procedure was used, and the final participants were randomly selected. Self‐developed and validated questionnaires and a nutrition education module appropriate for school‐aged pupils were used to collect data. A paired T‐test and an analysis of variance were used for statistical analysis. Details on the methods have been published (Adeoya, Akinwusi & Nagatomi, 2023).

**Results**: The findings demonstrated that peer groups significantly influenced participants’ nutritional awareness (t = 3.1, df = 99, *p* < 0.05; high PGI: mean = 33.8, SD = 10.5; low PGI: mean = 27.2 and SD = 8.4) and food choice (t = 3.2, df = 99, *p* < 0.05; high PGI: mean = 44.3, SD = 2.6; low PGI: mean = 42.43 and SD = 3.07). Furthermore, parental socioeconomic status significantly influenced the children's food choices (F_(4,95)_ = 3.892, *p* < 0.05) but not their nutrition awareness (F_(4,95)_ = 0.265, *p* > 0.05).

**Conclusion**: The constraint of a low socioeconomic background remains a practical barrier to healthy eating for children and an important limiting factor to quality eating behavior and variety of food despite adequate nutritional awareness. Nationalizing the school feeding program for public primary schools with adequate budgetary allocation and monitoring and evaluation by relevant stakeholders is recommended to ensure efficiency.

**Reference**

Adeoya, A. A., Akinwusi, A. T., & Nagatomi, R. (2023). Effectiveness of nutrition education in enhancing knowledge and attitude of pupils on choice of school mid‐day meal in Ibadan, Nigeria. *Food Science & Nutrition*, 00, 1–9. https://doi.org/10.1002/fsn3.3359

## Breastfeeding in public: urban design guidelines to increase women's comfort

Lisa Amir^1,2^, Julie Rudner^3^, Jenny Donovan^4^, Steph Amir^1^

^1^La Trobe University, Melbourne, Australia. ^2^Royal Women's Hospital, Melbourne, Australia. ^3^La Trobe University, Bendigo, Australia. ^4^Inclusive Design, Melbourne, Australia

**Background**: Some mothers find it challenging to breastfeed outside the home, and this may contribute to cessation of breastfeeding earlier than planned (Hauck et al., 2021). The physical environment can play an indirect role in supporting or deterring breastfeeding. Mothers in the UK reporting their neighbourhoods lacked safe play areas for children had lower rates of breastfeeding than other mothers (Peregrino et al., 2018).

Urban designers aim to make shared spaces – such as malls, streets, plazas and parks – welcoming for every member of the community. However, the needs of breastfeeding women are rarely considered. This project explored design features that invite or deter breastfeeding outside the home.

**Methods**: We conducted interviews and focus groups at Royal Women's Hospital, Melbourne, Australia in December 2018 to understand women's experiences when breastfeeding outside the home (n = 28). Specific focus groups included Indigenous families and one for women speaking Amharic, Arabic, Cantonese and Vietnamese. Interviews included women with a range of disabilities.

**Results**: Many participants reported avoiding breastfeeding in public spaces due to social expectations or physical comfort. Mothers reported that the best spaces for breastfeeding were dignified, safe, comfortable, accessible, compatible with their other needs and responsibilities with a high level of amenity. Some women preferred spaces that provide a balance between the need for privacy and the need for social connection.

We developed design guidelines that provide a range of spaces, enabling women to have their desired level of privacy. Our novel idea is to create semi‐private spaces – nooks, corners, pause places – where mothers can sit comfortably and still interact with others to the degree they wish, facilitated by low walls, chairs with high backs, screens or plants to provide a degree of privacy.

**Conclusions**: Breastfeeding‐friendly towns and cities are places where, in addition to dedicated breastfeeding spaces, much of the infrastructure that enables people to participate in and enjoy community life (workplaces, parks, shopping centres, community buildings) can also be comfortably and discreetly used for breastfeeding (Donovan et al., 2019). We recommend use of our new design guidelines for public institutions, councils, shopping centres, or other organisations designing or managing shared spaces.

**References**

Donovan, J., Rudner, J., & Amir, L. (2019). Here's how to make our cities breastfeeding‐friendly. The Conversation. https://theconversation.com/heres-how-to-make-our-cities-breastfeeding-friendly-110176

Hauck, Y. L., Bradfield, Z., & Kuliukas, L. (2021). Women's experiences with breastfeeding in public: An integrative review. Women & Birth, 34(3), e217‐e227.

Peregrino, A. B., Watt, R. G., Heilmann, A., & Jivraj, S. (2018). Breastfeeding practices in the United Kingdom: Is the neighbourhood context important? Maternal & Child Nutrition, 14(4), e12626.

## Clandestine labour: Australian parents’ experiences of returning to work and breastfeeding

Jennifer Ayton^1^, Alison Graham^2^, Sue Pearson^1^, Gemma Kitsos^1^, Emily Hansen^1^

^1^University of Tasmania, Hobart, Australia. ^2^Tasmanian Department of Health, Hobart, Australia

**Background**: Transitioning back to paid employment after the birth of a child is a significant adjustment period for both mothers and fathers. Balancing multiple responsibilities can intersect with work and family lives and breastfeeding practices. Understanding parents’ experiences around employment and infant feeding will help to inform workplace policies to better support working families who breastfeed.

**Methods**: This qualitative study undertaken in 2019, used one‐to‐one phone interviews to explore 42 Tasmanian public service employed mothers’ (n = 38) and fathers’(n = 4) experiences of transitioning back to work after the birth of their child and how they negotiated to breastfeed.

**Results**: The findings suggest that there is an unfolding narrative of returning to work whilst breastfeeding, one that starts with Leave‐taking, followed by a Return to and Doing work, and ends with parents’ creative use of Coupling strategies to mitigate stress. This narrative generates a form of work‐family breastfeeding conflict which occurs when work (whether part or full‐time) interferes with family breastfeeding life, and breastfeeding, including expressing work conflicts with work life. Consequently, expressing breastmilk and or breastfeeding becomes a type of clandestine labour; invisible work that is unrecognised as an integral part of the return to work for both parents, parental/maternal leave, and family‐friendly employment policies.

**Conclusions**: The concealed nature of the time and commitment that breastfeeding and expressing breastmilk involves for working postnatal families has public health implications for mothers, fathers, and infants. These include a negative impact on the duration of exclusive breastfeeding, stress on family and work relationships, workplace productivity, and retention.

## Understanding Motivations, Barriers, and Enablers to Skin to Skin Contact and Baby‐wearing in Mother‐Infant Dyads

Roisin Bailey^1^, Helen McIntyre^1^, Kate Thomson^2^, Merryl Harvey^3^

^1^University of Leicester, Leicester, United Kingdom. ^2^Birmingham City University, Birmingham, United Kingdom. ^3^Birmingham City University (retired), Birmingham, United Kingdom

**Background**: Skin‐to‐skin contact is a key component of breastfeeding support and the establishment of mother‐infant relationships (UNICEF UK, 2017). Skin‐to‐skin contact has recently been facilitated through baby‐wearing garments. However, little is known about the safety, efficacy, or experience of using such garments, or the maternal experience of engaging in skin to skin contact beyond the first hour after birth.

This study aimed to explore mothers’ experiences of facilitating skin‐to‐skin contact throughout the postnatal period with their healthy, term infants, and evaluate the use of baby‐wearing to facilitate ongoing skin to skin contact.

**Methods**: This study used a qualitative design, with semi‐structured interviews conducted at 6 weeks’ postnatal. Data collection took place in the homes of participants in the West Midlands, England. Thematic analysis utilised the health promotion theory of salutogenesis (Antonovsky, 1979).

Purposive sampling was used to enrol a subset of participants (n = 44) from a randomised controlled trial of the skin‐to‐skin contact facilitating garment (n = 98).

**Findings**: Initial engagement in skin to skin contact was motivated by mothers’ beliefs and attitudes, and their perception of social acceptability and expectation. Relational bonding, and facilitating breastfeeding and lactation, both motivated mothers to begin, and to continue to engage in, skin to skin contact.

Mothers engaged in skin‐to‐skin contact when they were able to establish privacy, modesty, agency, and autonomy; garments were used to varying degrees to facilitate this. Mothers who continued to engage had social support, and an understanding of its benefits.

The value of continued skin to skin contact was felt most significantly by mothers who had ceased exclusive breastfeeding before they intended to. In these circumstances, skin to skin contact was used either to safeguard or repair the dyadic relationship.

**Conclusion**: Baby‐wearing offers an extension the concept of kangaroo mother care to this population of healthy infants and their mothers. Baby‐wearing garments are a feasible method of supporting ongoing engagement in skin‐to‐skin contact for mothers and their healthy, term babies, with high acceptability. Provision of garments may mitigate some of the experiential barriers described by mothers engaging in ongoing skin to skin contact.

**References**

Antonovsky, A. (1979). Health, Stress, and Coping. San Francisco, California: Jossey‐Bass Inc.

UNICEF UK. (2017). Guide to the UNICEF UK Baby Friendly Initiative Standards. (2nd ed.) London: UNICEF UK.

## Eyes on the Baby: Design and implementation of multiagency SUDI prevention

Helen Ball^1^, Laura Murray^1^, Louise Cooper^1^, SUDI Prevention Steering Committee^2^

^1^Durham University, Durham, United Kingdom. ^2^County Durham, Durham, United Kingdom

**Background**: Sudden unexpected infant deaths (SUDI) now predominantly cluster in vulnerable families living in impoverished communities (Pease et al., 2020). The 2020 National Safeguarding Practice Review report found that families experiencing infant deaths in 2018 were already known to multiple services including safeguarding, drug and alcohol, probation, care leavers, and mental health (NCSPR 2020). The review recommended implementation of multi‐agency working to enhance targeted SUDI prevention.

**Methods**: Funded by the North‐East and North Cumbria ARC (Applied Research Collaboration) Eyes on the Baby is a co‐produced project led by Durham University's Infancy & Sleep Centre, Durham County Council Public Health Team, and County Durham Safeguarding Children Partnership to implement multi‐agency SUDI prevention in County Durham. Using Normalisation Process Theory as an implementation framework (Murray et al., 2010) we have trained the County Durham council and allied services workforce to be alert to the circumstances in which SUDI occurs, to explore infant sleep practices with families, and to offer information and signpost to support.

**Results and conclusions**: Regular contact with the front‐line workforce allows us to sustain awareness of SUDI prevention via staff forums and a network of Eyes on the Baby champions embedded within teams. Ongoing evaluation of both implementation and outcomes reported by staff and families allow us to better understand multiagency approaches to specialist public health issues in general and SUDI prevention in particular, and to determine whether this approach warrants broader roll‐out in other local authorities.

## Anya: Changing the landscape of parenting and infant feeding support

Shel Banks^1^, Chen Mao Davies^2^

^1^LatchAid, Preston, United Kingdom. ^2^LatchAid, Cirencester, United Kingdom

**Background**: Anya is a digital parenting and breastfeeding app (Apple/Android), supporting parents through the first 1001 days of a child's life, from conception to their 2nd birthday, with timely and trustworthy information and support.

Anya has been created with leading infant feeding and maternal health experts and is designed to raise the confidence and skill level of parents to feed, parent and feel supported to make better informed choices, thereby contributing to longer‐term improved breastfeeding rates and related positive public health outcomes.

**Methods**: Anya is a unique and award‐winning WHO Code compliant feeding app, funded by Innovate UK and The Q Foundation.

Available worldwide, with over 10,000 users in over 100 countries, LatchAid is free at the point of entry, ORCHA certified, with upgrades to premium usage prescribed by the NHS and partners across the UK.

In 2022 the Government's Small Business Research Initiative (SBRI) provided funding to further pursue positive results in dealing with inequalities, using coproduction in 2 community programmes in England over a 3‐6 month period.

The SBRI enables the NHS to access innovations that solve unmet needs: an accelerated access collaborative initiative in partnership with the AHSN network.

**Findings:** Our 2021 App pilot demonstrated users’ improved exclusive breastfeeding rates and positive reports about usage from professionals and families.

In this presentation we plan to share with you the initial findings from our 2023 report on the learning from the SBRI funded initiative, specifically targetting health inequalities in infant feeding and perinatal health across the UK.

**Conclusion:** The app uses cutting‐edge interactive CGI 3D technology to give an anatomical view of babies attaching at the breast in optimal positions and a variety of poses, with 360° camera control and customisable avatars, alongside moderated chat channels, a revolutionary Artificial Intelligence powered virtual breastfeeding supporter providing tailored 24/7 responses, timely access to IBCLCs and other experts in the field, and regular live expert webinars and virtual drop‐ins.

Anya is designed to address the need for remote support and innovative approaches to supporting families with parenting, infant feeding and parenting, in today's world and tomorrow's.

**References**

[1] Staff, M., University of Exeter & South West AHSN (2022): LatchAid NHS Pilot Outcomes report.

[2] Breastfeeding UK average rate is 47.6% Nuffield Trust (2020/1): https://www.nuffieldtrust.org.uk/resource/breastfeeding#background

## We know what we measure, but what are we measuring? A comparison of the measurement of breastfeeding practices using data from rural Pakistan

Jo‐Anna Baxter^1,2^, Yaqub Wasan^3^, Amjad Hussain^3^, Alison Daniel^4^, Sajid Soofi^3^, Zulfiqar Bhutta^1,2,3^, Deborah O'Connor^1,5^

^1^Department of Nutritional Sciences, University of Toronto, Toronto, Canada. ^2^Centre for Global Child Health, Hospital for Sick Children, Toronto, Canada. ^3^Aga Khan University, Karachi, Pakistan. ^4^World Health Organization, Geneva, Switzerland. ^5^Translational Medicine, Hospital for Sick Children, Toronto, Canada

**Background:** Exclusive breastfeeding (EBF) offers undeniable benefits for infant health and development (WHO 2002; Victora et al., 2016). To understand the proportion of exclusively breastfed (EBF) infants 0‐5.9 months, many low‐ and middle‐income countries rely on national surveys that use a point prevalence estimate of intake over the past 24‐h. This estimate can be misunderstood as the proportion of children with an EBF duration to 6‐months, although the measure actually represents the prevalence of EBF among children <6 months (Pullum 2014). Given the importance of this measure to national‐level EBF policy‐setting, accurately measuring EBF is crucial.

**Methods:** Using data from a nested‐substudy within the MaPPS Trial in rural Pakistan (ClinicalTrials. gov: NCT04451395), we aimed to (1) describe feeding practices among 3‐month infants approached for study recruitment (n = 356) and compare with national‐level estimates; and (2) determine the reliability of prevalence estimates of EBF using the standard 24‐h recall method and an adapted 2‐week recall among recruited infants with complete data (n = 172). Mothers of recruited infants had to report practicing EBF or predominantly breastfed (PBF). Infant recall data was used to classify breastfeeding practices into four groups: EBF (infant received breastmilk only); PBF (breastmilk and/or water or nonnutritive liquids [NNL; e.g., gripe water, rice water]); mixed feeding; and no breastfeeding. All data was collected by highly‐trained female datal collectors in a district with a long‐standing, community‐based research program.

**Findings:** From national‐level data, 52.1% of Pakistani infants 2‐3 months are estimated to be EBF (NIPS 2013). Among infants approached for study recruitment, we found 12.4% were EBF, 52.8% PBF, 28.7% mixed feeding, and 6.2% no breastfeeding. For infant with both measures (24‐h vs. 2‐week recall), we found similar estimates of EBF (17.4% vs. 16.3%) and PBF (82.6% vs. 83.7%). Among PBF infants, in addition to breastmilk, 15% received water; 45% NNL; and 40% received both water and NNL.

**Conclusion:** We found that EBF estimates were comparable between recalls and breastfeeding was widely practiced using data collected by trusted female study personnel. However, EBF rates were much lower than national estimates. This could reflect response bias within the national‐level data, and should be investigated further.

**References**

National Institute of Population Studies (NIPS) and ICF International. (2013). *Pakistan Demographic and Health Survey 2012‐13*. Maryland, USA: NIPS and ICF International.

Pullum T. W. (2014). Exclusive breastfeeding: aligning the indicator with the goal. *Global health, science and practice*, 2(3), 355–356. https://doi.org/10.9745/GHSP-D-14-00061

Victora, C. G., Bahl, R., Barros, A. J., França, G. V., Horton, S., Krasevec, J., Murch, S., Sankar, M. J., Walker, N., Rollins, N. C., & Lancet Breastfeeding Series Group. (2016). Breastfeeding in the 21st century: epidemiology, mechanisms, and lifelong effect. *Lancet*, 387(10017), 475–490. https://doi.org/10.1016/S0140-6736(15)01024-7

World Health Organization. (2002). *Global strategy for infant and young child feeding*. Geneva: World Health Organization.

## Breastfeeding experiences of women with perinatal mental health conditions: A systematic review and thematic synthesis

Hayley Billings, Janet Horsman, Hora Soltani, Rachael Spencer

Sheffield Hallam University, Sheffield, United Kingdom

**Background:** Early cessation of breastfeeding is particularly reported amongst women with perinatal mental health (PMH) conditions which deprives such mothers and their infants of its widely known benefits (Dennis and McQueen, 2007; Tanganhito et al., 2020; Victora et al., 2016). Exploring experiences of breastfeeding among women with a variety of PMH health conditions can elucidate key information on barriers and facilitators to inform health intervention strategies.

**Methods:** A systematic search of five electronic databases was carried out. Study selection, data extraction and critical appraisal of included studies were undertaken by two authors. Data was synthesised and coded thematically using NVivo software, with illustrative quotes used to support the themes.

**Findings:** The review included 17 full text articles. Included studies were published between 2003 and 2021 with settings in 9 different countries (Australia, Canada, Democratic Republic of Congo, Ghana, Ireland, New Zealand, Sweden, UK and USA). PMH conditions varied between studies and included: postnatal depression, emotional/mental distress, postnatal blues, mood disorder, severe mental illnesses, eating disorders, obsessive compulsive disorder, and traumatic childbirth/Posttraumatic stress disorder.

Themes and subthemes included: (1) Vulnerabilities: Expectations versus reality; Self‐perception as a mother; Isolation (2) Positive outcomes: Bonding and closeness; Sense of achievement (3) Challenges: Striving for control; Inconsistent advice and lack of support; Concerns over medication safety; and Perceived impact on milk supply.

**Conclusion:** The intensity and magnitude of positive outcomes that women described, and the challenges experienced were exacerbated in mothers with PMH conditions. Positive experiences enhanced mothers’ self‐esteem and confidence and were described as having a potential for healing. The challenging experiences were particularly influenced by a lack of support, shame, fear of stigmatisation and worries over medication safety.

Appropriate social and professional support, clear and consistent advice, and adequate antenatal education are required to ensure that women are aware of the potential challenges associated with breastfeeding during the early neonatal period. Further research into medication safety, use of alternative medication and clearer guidance on the suitability of each type of medication during breastfeeding are urgently needed.

## Reducing readmissions for newborn jaundice in a GOLD accredited UNICEF BFI maternity service

Jennifer Birtwistle and Susan Henry

East Lancashire Hospitals NHS Trust, East Lancashire, United Kingdom

**Background:** East Lancashire Hospitals NHS Trust (ELHT) Maternity service was the first service to receive the UNICEF B.F.I. GOLD award in 2017. The four themes of the UNICEF GOLD standards, leadership, culture, monitoring and progression, have really helped our services to achieve long term sustainability and progress interventions to improve parent experiences. By monitoring our jaundice readmissions, we have been able to collaborate, action plan and progress to reduce these readmissions. Our aim is to keep families together at home and optimise the feeding experience.

**Findings:** Our data demonstrates that, particularly during the covid‐19 pandemic in 2020, there was a sharp incline in jaundice readmissions, specifically in the latter half of the year moving into early 2021. This could have been related to several national lockdowns, reduced maternity home visiting, an eagerness to be discharged home at the earliest convenience by either health professionals or the family themselves, as well as reduced face‐to‐face peer support.

It is well known that late preterm/early term infants (34 – 38 + 6) are at increased risk of hyperbilirubinemia (Boies and Vaucher, 2016) and this was a recurrent theme identified amongst our jaundice readmissions, as well as babies that had previously been treated for jaundice and/or had early feeding difficulties. Task and finish groups were set up alongside our GOLD leadership team to develop action plans moving forwards targeting this specific group of babies in particular.

**Conclusion:** Through robust monitoring, action planning and 3 monthly reviews, we were able to reduce jaundice readmissions from 43 in quarter 1 of 2021 to 27 in quarter 1 of 2023 (1.2% decrease per total births in quarter). This resulted in less disruption in the postnatal period to mothers, babies and their families, and optimisation of their feeding experiences.

**Reference**

Boies, E. and Vaucher, Y., 2016. ABM Clinical Protocol #10: Breastfeeding the Late Preterm (34–36 6/7 Weeks of Gestation) and Early Term Infants (37–38 6/7 Weeks of Gestation), Second Revision 2016. Breastfeeding Medicine, 11(10), pp. 494‐500.

## Influence of marketing via digital platforms observed across seven international studies (Indonesia, Vietnam, India, Thailand, Nigeria, Philippines and Mexico) that impact knowledge and behavior of pregnant women, mothers and health workers

Brenda K. Brewer^1^, Nadine Nasser^2^

^1^Westat, Rockville, MD, USA. ^2^Access to Nutrition Foundation, Utrecht, Netherlands

**Background:** The aim of “The Code” and subsequent WHA resolutions is to end inappropriate promotion of breastmilk substitutes (BMS) for infants and young children up to 36 months (WHO, 1981). The Code prohibits promotion, including via media, of covered products to health workers, mothers and pregnant women. Recent studies including global review of Code violations across 95 countries reveals marketing promotions (e.g., print and social media) were the most common violation reported, and online purchasing and BMS social media sites are more prevalent (Becker, 2022). A WHO study found that baby food companies are paying social media platforms and influencers to gain direct access to pregnant women and mothers (WHO, 2022). The WHO status report of national implementation noted of 144 countries with legal measures on the Code, only 37 explicitly mention BMS promotion on digital platforms. No legislation prohibiting BMS promotions explicitly exempted digital marketing (WHO, 2022). Study results from seven country assessments (Vietnam [2015], Indonesia [2015], India [2016], Thailand [2017], Nigeria [2017], Philippines [2020], Mexico [2020‐2021] documented online retail promotions and social media marketing.

**Methods:** Studies in seven countries assessed online retail promotions and social media following NetCode Protocol for Media Monitoring (WHO, 2017). Staff monitored selected websites for 8 weeks (4 weeks in Vietnam/India). Mothers were interviewed in all countries except Mexico, due to covid restrictions. Data included: mothers’ report of promotion; promotions observed in selected online retailers and social media.

**Findings:** In all countries except Thailand, online media promotions far exceeded traditional media promotions. In all countries online retail promotions were greater than promotions in physical retail outlets. However, online retailer promotions were fewest in India and Nigeria where regulations are more Code‐aligned. Similarly, the fewest number of mothers’ reports of internet messages or promotions was in the Philippines and Indonesia.

**Conclusions:** Extensive promotions of BMS products on online retail platforms is concerning. Online promotions and contact with mothers is increasing in all countries through social media and companies’ own websites. National Code laws can help regulate online promotions and can be strengthened by explicitly addressing digital BMS marketing.

**References**

Becker, G.E., et al., (2022) Global evidence of persistent violations of the International Code of Marketing of Breast‐milk Substitutes: A systematic scoping review. Maternal & Child Nutrition 18(S3):e13335.

World Health Organization (WHO). (1981). International Code of Marketing of Breast‐milk Substitutes. Retrieved from: International Code of Marketing of Breast‐Milk Substitutes (who. int).

World Health Organization (WHO). (2022). How the marketing of formula milk influences our decisions on infant feeding. Retrieved from: Maternal, Newborn, Child and Adolescent Health and Ageing Department (who. int).

World Health Organization (WHO). (2017). Monitoring the marketing of breast‐milk substitutes: protocol for periodic assessment. Retrieved from: NetCode toolkit: monitoring the marketing of breast‐milk substitutes: protocol for periodic assessments (who. int).

World Health Organization (WHO). (2022). Marketing of breast‐milk substitutes: national implementation of the international code, status report 2022. Retrieved from: Marketing of breast‐milk substitutes: national implementation of the international code, status report 2022 (who. int).

## The emotional rollercoaster of lactation care: How can we better protect those providing breastfeeding support?

Amy Brown

Swansea University, Swansea, United Kingdom

**Background:** We know that infant feeding and mental health are closely linked. Breast and bottle feeding can bring about a range of emotions from pride and reward to regret and feelings of shame. Many women talk about how even though breastfeeding can feel challenging, they still want to carry on because it is important to them. But what about the wellbeing of those doing the supporting? The mental health of midwives, health visitors and other health professionals has hit the headlines, particularly during Covid‐19. A growing number of studies have examined concepts of stress, burnout, and resilience in these groups (Coetzee S. & Klopper 2010, Hunter et al., 2019). But although lactation support is part of their role, the specific emotional impact of trying to support breastfeeding and families in a society that often does not protect it, is often not examined. Moreover, the stories of those in specific lactation roles such as IBCLC, breastfeeding counsellors, and peer supporters are missing (Kendall‐Tackett 2013, Brown 2022). The aim of this study was therefore to examine the experiences and needs of those working in lactation support.

**Methods:** A two‐part online survey conducted before (n = 727) and again during Covid‐19 (n = 520) that examined the mental health and motivations of a range of professionals and supporters working in lactation. Concepts of burnout, empathy and imposter syndrome were examined alongside specific stressors and motivators of the role

**Results:** Personal and work‐related burnout were high, rising further during Covid‐19. However, burnout in relation to support families was low with this aspect of the work often the primary driver to continuing. Lactation supporters were brought to the work through their own challenging experiences, desire to drive systemic change and high levels of empathy. Stressors of the role included of a lack of investment in breastfeeding support, financial pressures, and conflict with those who do not think that breastfeeding is important. However, motivation to continue was heightened by the reward of being able to help, relationships with colleagues and pride and identity in the role.

**Conclusions:** Alongside support for breastfeeding women, investment is needed to protect and nurture those doing the care with clear strategies to support wellbeing on an individual and systemic level. Both families and lactation supporters would be protected by better investments in an environment that better supported breastfeeding.

**References**

Brown, A. (2022). The compassion code: power, perseverance and passion in lactation support

Coetzee S. & Klopper H. (2010). Compassion fatigue within nursing practice: A concept analysis. Nursing & health sciences, 12(2):235‐43.

Hunter, B., Fenwick, J., Sidebotham, M. and Henley, J., (2019). Midwives in the United Kingdom: Levels of burnout, depression, anxiety and stress and associated predictors. Midwifery, 79, p.102526.

Kendall‐Tackett K. (2013). Caring for Ourselves When Caring for Others: What Lactation Consultants Need to Know About Compassion Fatigue. Clinical Lactation, 1;4(4):137‐8.

## Delivering Early Breastfeeding Support (DEBS) providing antenatal and proactive postnatal breastfeeding support by a dedicated infant feeding team

Caroline Buchan and Sharon Gilchrist

NHS Lothian, Livingston, United Kingdom

**Background:** The Scottish Government's stretch aim is to reduce the breastfeeding attrition rate at 6‐8 weeks by 10% by 2025. The MIN Survey (Scottish Government, 2018) and the Breastfeeding Series from The Lancet (2016) were key determinants in driving the national priority to support breastfeeding and an evidence base to target support for women, focusing on attrition.

NHS Lothian's breastfeeding drop off rate at 10‐14 days in 2019‐2020 was 13%, in West Lothian it was 23%. The Scottish Government therefore prioritised funding for the DEBS project in West Lothian. An aim to reduce the attrition rate of any breast milk by 2% at 10‐14 days was agreed.

**Method:** DEBS uses Quality Improvement Methodology and is based on the FEST study (Hoddinott, Craig, Maclennan, Boyers, & Vale, 2012) The model for improvement has provided a method to build the evidence about what works implementing change ideas through PDSA/iterative testing to develop a care bundle. This support spans both maternity and health visiting services.

**Results:** DEBS has improved the drop off rate for any breast milk by 44% at first Health Visitor visit in the chosen area. Exclusive breastfeeding rates in DEBS are currently 54% at first Health Visitor visit and 42% at 6‐8 weeks in comparison to 34% and 28% across Scotland.

**Conclusion:** DEBS provides women with access to practical 1:1 antenatal and postnatal breastfeeding support, tailored to individual needs, in line with the Scottish Government Stretch aim. DEBS supports parent‐ infant relationships, gives women confidence to tackle social and cultural pressures and could positively impact the current environmental crisis whilst reducing the financial burden on the NHS.

**References**

Hoddinott, P., Craig, L., Maclennan. G., Boyers, D., & Vale, L. (2012). Process evaluation for the Feeding Support Team (FEST) randomised controlled feasibility trial of proactive and reactive telephone support for breastfeeding women living in disadvantaged areas. BMJ Open, 2, 1‐12. https://doi.org/10.1136/bmjopen-2012-001039

Lancet. (2016). Breastfeeding Series. Retrieved from: https://www.thelancet.com/series/breastfeeding

Scottish Government. (2018). Scottish Maternal and Infant Nutrition Survey 2017. Retrieved from: https://www.gov.scot/publications/scottish-maternal-infant-nutrition-survey-2017/pages/1/

## Stakeholder and parent Coproduction within an NHS‐tailored evidence synthesis of breastfeeding support in the UK: The Action4Breastfeeding project

Phyll Buchannan^1^, Joyce Marshall^2^, Alison McFadden^3^, Shona Shinwell^3^, Albert Farre^3^, Sara Cumming^3^, Laura Hay^3^, Anna Gavine^3^

^1^Breastfeeding Network, Newcastle upon Tyne, United Kingdom. ^2^University of Huddersfield, Huddersfield, United Kingdom. ^3^University of Dundee, Dundee, United Kingdom

**Background:** Coproduction in research enhances the quality and relevance of research findings. Stakeholder engagement was integral to the Action4Breastfeeding project. Our approach to stakeholder and parent involvement was ‘active involvement’ (Pollock et al., 2018) throughout the process of evidence synthesis including planning, production and dissemination.

**Methods:** Engagement was achieved through a stakeholder working group and a parents’ panel, supplemented by focus group discussions with women from groups least likely to breastfeed. All four UK countries were represented. The stakeholder working group included members of third sector organisations, policymakers, NHS practitioners, and commissioners. The parents’ panel included women who had breastfed a child within the last 3 years and fathers. Focus group participants were recruited by a peer support organisation working with disadvantaged families. The three groups met separately four times during the study. Coproduction activities included: a) discussing priorities for breastfeeding support, b) agreeing criteria to assess transferability of effective interventions from global evidence to the UK setting; c) identifying barriers to implementing breastfeeding support in NHS settings, and d) developing and prioritising strategies to overcome the identified barriers. The online meetings were supplemented with a modified Delphi survey. Focus groups were held online and in‐person. Members of the stakeholder working group and parents panel attended in‐person workshops held around the UK as the final stage of the project. A project extension for women with multiple long‐term conditions mirrored this work.

**Findings:** Practical elements of engaging stakeholders and parents in co‐creation within an evidence synthesis will be presented, including achieving trust, confidentiality, and good working relations, valuing contributions and maintaining engagement. Examples of diversity and differing priorities within the various stages of the evidence synthesis will be provided. Ways of managing these to produce an NHS‐tailored implementation and evaluation strategy framework will be discussed. We plan to continue engagement beyond the funded project to ensure effective knowledge mobilisation (Grindall et al., 2022).

**Conclusion:** Co‐creation was essential to this evidence synthesis and whilst not always easy to achieve, resulted in rich discussions and diverse priorities that will contribute to the development of cost‐effective breastfeeding support interventions in the UK.

**References**

Grindell, C., Coates, E., Croot, L. et al., (2022). The use of Coproduction, Codesign and co‐creation to mobilise knowledge in the management of health conditions: a systematic review. BMC Health Serv Res 22, 877.

Pollock, A., Campbell, P., Struthers, C., Synnot, A., Nunn, J., Hill, S. et al., (2018). Stakeholder involvement in systematic reviews: a scoping review. Systematic reviews. 7(1):208.

## Connecting the dots between foetal, premature and full‐term behaviour while in skin‐to‐skin contact: The nine stages of instinctive behaviour

Karin Cadwell and Kajsa Brimdyr

Healthy Children Project, Harwich, MA, USA

**Background:** The competence of a newborn born prematurely may be the direct result of behaviour training that begins during the first 12 weeks of foetal life.

**Method:** Using Widström's 9 Instinctive stages as the template, we use the full‐term newborn's instinctive behaviour during the first hour after birth while skin‐to‐skin and the movements of the fetus by 20 weeks’ gestation to hypothesise the pre‐feeding behaviors of pre‐term infants while being held skin‐to‐skin.

**Findings:** The fetus has invested in learning specific tasks, in a specific order, evolutionarily necessary to optimise the chance of survival. (Widström et al., 2020). The premature infant can be seen in videos to progress through the same stages as does the full‐term newborn when in skin‐to‐skin contact with the mother, albeit over days and weeks, rather than 1 h. An important template of behaviours is developed in utero and activated after birth. The survival mechanism is a pattern and sequence that was practised as intrauterine movements of the fetus and then implemented as the full‐term newborn's patterns of movement during the first hour after birth when skin‐to‐skin with the mother (Widström et al., 2019). The preterm infant also has the ability to go through these important patterns of movement, when given the opportunity to be skin‐to‐skin with their parent. The template of behaviours described as the 9 stages, the Birth Cry, Relaxation, Awakening, Activity, Rest, Crawling, Familiarisation, Suckling, and Sleeping, have been developed and practised in utero, in the same specific order.

**Conclusion:** Both the full‐term newborn and the preterm infant have been training for and are prepared for this experience after birth: to find the breast and to initiate breastfeeding. Rigorous research is needed to examine not just the premature infant's suckling behaviour, but the journey to suckling. All newborns, whether full‐term or premature, should be guaranteed the opportunity to utilise their template of practised survival skills while experiencing skin‐to‐skin contact.

**References**

Brimdyr, K., & Cadwell, K. (2021). Connecting the dots between fetal, premature and full‐term behaviour while in skin‐to‐skin contact: The nine stages of instinctive behaviour. Breastfeeding Review, 29(3), 17–24.

Widström, A.‐M., Brimdyr, K., Svensson, K., Cadwell, K., & Nissen, E. (2019). Skin‐to‐skin contact the first hour after birth, underlying implications and clinical practice. Acta Paediatrica, 108(7), 1192–1204. https://doi.org/10.1111/apa.14754

Widström, A.‐M., Brimdyr, K., Svensson, K., Cadwell, K., & Nissen, E. (2020). A plausible pathway of imprinted behaviors: Skin‐to‐skin actions of the newborn immediately after birth follow the order of fetal development and intrauterine training of movements. Medical Hypotheses, 134, 109432. https://doi.org/10.1016/j.mehy.2019.109432

## Considering the Impact of Powdered Baby Formula in Planetary and Population Health Policy

Karin Cadwell, PhD, Anna Blair, PhD, Cindy Turner‐Maffei, MA, & Kajsa Brimdyr PhD

Healthy Children Project, Harwich, MA, USA

**Background:** Breastfeeding is situated in the nexus between the health of the earth's environment and the health of its population. Although human milk feeding has negligible cost to the environment and is the undisputed foundation of optimal population health, around two‐thirds of humans are fed processed foods, such as powdered baby formula, as a substitute for human milk in the first 2 years after birth.

Production‐to‐gate assessment of the environmental cost of powdered standard infant, special, follow‐on and growing‐up formula data for Egypt and more than a dozen other countries in the Asia Pacific region, North America, South America and Europe have been calculated.

**Method:** Compute a minimal estimate of greenhouse gas (GHG) “farm to gate” emissions for powdered baby formula products sold using data obtained from Euromonitor. Translate the CO_2_ eq. emissions from powdered baby formula into easily understood comparisons using the Greenhouse Gas Equivalencies Calculator.

**Findings:** Powdered infant formula is a highly processed food that contributes GHG emission for a kilogram of milk formula (including standard infant formula, follow on formula, and toddler milk), from a low of around 4 kg CO_2_ eq. in Asia Pacific countries to a high of more than 10 kg CO_2_ eq. for “growing up” powdered formula in North America.

**Conclusion:** Along with the acknowledged health and social benefits of breastfeeding, the cost to the environment should be considered in developing and funding infant feeding policies and supportive practices.

Andresen, E. C., Hjelkrem, A.‐G. R., Bakken, A. K., & Andersen, L. F. (2022). Environmental Impact of Feeding with Infant Formula in Comparison with Breastfeeding. *International Journal of Environmental Research and Public Health*, *19*(11), 6397. https://doi.org/10.3390/ijerph19116397

Cadwell, K., Blair, A., Turner‐Maffei, C., Gabel, M., & Brimdyr, K. (2020). Powdered Baby Formula Sold in North America: Assessing the Environmental Impact. *Breastfeeding Medicine*, bfm.2020.0090. https://doi.org/10.1089/bfm.2020.0090

Dadhich, J., Smith, J. P., Iellamo, A., & Suleiman, A. (2021). Climate Change and Infant Nutrition: Estimates of Greenhouse Gas Emissions From Milk Formula Sold in Selected Asia Pacific Countries. *Journal of Human Lactation*, *37*(2), 314–322. https://doi.org/10.1177/0890334421994769

Karlsson, J. O., Garnett, T., Rollins, N. C., & Röös, E. (2019). The carbon footprint of breastmilk substitutes in comparison with breastfeeding.

## Bainne na Beatha (Milk of Life) and Beyond: Abductive analysis of narrative interviews about infant feeding issues for parents in Ireland during the pandemic

Tanya Cassidy

Maynooth University, Maynooth, Ireland

**Background:** Maternity services in Ireland are some of the oldest in the world, but they have also provoked some strong critical discussions especially in this century. At the same time, Ireland has some of the lowest rates of breastfeeding in the world, although these rates have been rising and continue to rise throughout the pandemic. We investigated, using innovative combinations of methods and abductive analysis, experiences of becoming parents during the pandemic, which included infant feeding experiences. We offer discussions treating both difficulties and benefits related to the pandemic, especially in relation to whether or not the birth took place in hospital or at home, and for early home discharge experiences which combines the best of both.

**Methods:** Our research was funded by two Irish Research Council (IRC) New Foundations grants, and were supported by two community groups (Irish Neonatal Health Alliance [INHA] and Friends of Breastfeeding). We received ethical approval from Dublin City University (DCU).

We reached over one hundred and fifty people who received project information and a consent form, with over one hundred completing the interview in the end. Each interview followed best practice for narrative interviews, and lasted approximately 1 h.

Using Abductive Analysis we reviewed these extensive data and are currently preparing a full length manuscript, but we will meanwhile highlight some of the results in this paper.

**Finding:** The method itself (narrative interviews) are the key to our discussion. Not only does this method benefit research by offering far more detailed information, including details about place of birth (home vs. hospital), infant feeding issues such as the ‘top up trap’, but also how to overcome this trap and ultimately to experience a long‐term breastfeeding journey, even under adverse circumstances.

Meanwhile the method also benefits many participants, for whom telling their stories has an almost therapeutic quality, where breastfeeding often had heightened expectations following the difficulties associated with their birth experiences. In addition, people talked hoping that their stories could be used to help others to avoid such negative experiences, but at the same time retain some of the positive aspects to help parents regarding their infant feeding journeys in a culture where breastfeeding is still fighting to become the norm.

At the same time, this method takes a lot longer and in the rapid production of knowledge we saw during the height of Covid 19, this can be a problem. At the same time, because of the technological links associated with online communication that became much more common for most people during the pandemic, it meant that conducting Zoom interviews with people who have young children was considered the best way to enable people to participate. We had discussions about the advantages, as well as the disadvantage for breastfeeding that the move to online had for many.

Despite the fact that we did have an unprecedented response to our project, in the end we need to recognize that this is a qualitative project, and therefore gaps and limitations exit in our data. One clear limitation is the lack of racial diversity among our participants. Similarly most respondents were well educated, and many were from mid to high socioeconomic backgrounds.

**Conclusions:** There have been a huge number of surveys conducted with people who became parents during the pandemic, some of which also discuss infant feeding experiences during the pandemic. We however collected detailed narrative accounts, capturing historical testimonies of the major historical event which so impacted not only maternity health services around the world, but also breastfeeding rates, especially in countries like Ireland where these rates significantly increased throughout the pandemic.

## Who Gets to Breastfeed? A Narrative Ecological Analysis of Women's Infant Feeding Experiences in the UK

Joanne Clarke^1^, Gill Thomson^2^, Jenny Ingram^3^, Debbie Johnson^3^, Kate Jolly^1^

^1^University of Birmingham, Birmingham, United Kingdom ^2^University of Central Lancashire, Preston, United Kingdom. ^3^University of Bristol, Bristol, United Kingdom.

**Background:** Research suggests that infant feeding should be seen as part of a complex system with interacting factors at different ecological levels (individual, social/community networks, cultural/institutional) affecting individual women's behaviours (Trickey et al., 2018). Although, as yet, more work is needed to implement an ecological approach in breastfeeding programmes. In our study we aimed to understand how factors operating at different levels—the mother‐infant dyad, the health service, family and social networks, and wider community infrastructure—interacted with women's motivations and experiences of breastfeeding.

**Methods:** A secondary analysis of interviews with first‐time mothers (n = 24), all of whom planned to breastfeed, was undertaken as part of the Assets‐based feeding help Before and After birth (ABA) feasibility trial (Clarke et al., 2020). A complex‐systems lens approach was used to explore interactions between multi‐level factors and women's experiences. We used categorical content analysis (Iborra, 2007; Gondim and Bendassolli, 2014) to investigate interrelationships between key factors and identify different infant feeding typologies.

**Findings:** We identified two typologies: “by hook or by crook” (n = 17) women persisted with breastfeeding despite hurdles, whereas “disappointed” (n = 7) women ceased breastfeeding early. Differences between the typologies were seen at sociodemographic, social and service level. “By hook or by crook” women showed more proactive help‐seeking behaviours, accessed wider support and had positive experiences of support. “Disappointed” women were more likely to be White‐British, younger, to have considered mixed‐feeding antenatally and have experienced negative support. Their babies were more likely to have received unexpected “top‐ups” and be seen as having feeding difficulties.

**Conclusion:** The ecological approach moves the focus away from women's individual decisions to consideration of the multi‐level factors needed to be in place to enable successful breastfeeding. Encouragement of help‐seeking behaviours and improvement of facilities, support and services is required.

**References**

>Clarke, J. L., Ingram, J., Johnson, D., Thomson, G., Trickey, H., Dombrowski, S. U., et al., (2020). The ABA intervention for improving breastfeeding initiation and continuation: feasibility study results. Matern. Child Nutr. 16, e12907. https://doi.org/10.1111/mcn.12907

Gondim, S. M. G., and Bendassolli, P. F. (2014). The use of the qualitative content analysis in psychology: a critical review. Psicol. Estudo 19, 191–199. https://doi.org/10.1590/1413-737220530002

Iborra, A. (2007). “A content analysis of narratives from a categorical and holistic point of view to study changes after a rite of passage”, in Capturing Identity: Quantitative and Qualitative Methods, eds M. Watzlawik, and A. Born (Lanham, MD: University Press of America), 39–52.

Trickey, H., Thomson, G., Grant, A., Sanders, J., Mann, M., Murphy, S. and Paranjothy, S. (2018). A realist review of one‐to‐one breastfeeding peer support experiments conducted in developed country settings. Matern. Child Nutr. 14, e12559.

## Parents' and professionals' views and experiences of support for close and loving parent–infant relationships

Nicola Crossland^1^, Lucy Cross^1^, Gill Thomson^1^, Victoria Moran^1^, Reyhan Furman^1^, Cath Coucill^1^, Sue Henry^2^, Sophie Henry^1^

^1^University of Central Lancashire, Preston, United Kingdom. ^2^East Lancashire Hospitals NHS Trust, Blackburn, United Kingdom

**Background:** Support for breastfeeding is a fundamental element of maternal and child healthcare [1]. The UNICEF UK Baby Friendly Initiative (BFI) is a staged accreditation programme providing practice standards to support infant feeding and parent‐infant relationship building in healthcare and community settings [2]. In the UK, the BFI standards were revised in 2012 to include a focus on supporting a close parent–baby relationship and responsive infant feeding; this element of the standards has not yet been researched. Our qualitative interview study explored the views, experiences and needs of mothers and birthing parents, and infant feeding professionals, to ascertain what matters to these stakeholders in receiving or providing support for parent–infant relationships during infant feeding care.

**Methods:** In‐depth interviews were carried out with mothers and birthing parents who had had a baby in the previous 12 months from three areas in North‐West England where both the maternity and community services were fully BFI accredited, and with professionals offering infant feeding care employed at a fully BFI accredited organisation anywhere in the UK. Participants were recruited via social media and professional networks. Semi‐structured interviews were conducted online via videocall, recorded, and transcribed. Thematic analysis [3] was used to identify key themes.

**Findings:** Twelve mothers aged between 29 and 39 years took part. Five were first‐time mothers. Seven had exclusively breastfed their most recent baby while five had combined breast and formula feeding. Seven health professionals providing infant feeding care with a mean of 8 years' experience took part. Overarching themes described the ‘Components of a relationship focus’, ‘How to have a relationships focus’, ‘Consequences of a relationships focus’ and ‘Enabling the relationships focus’. Mothers’ priorities were breastfeeding knowledge, maternal mental health, and mother–baby bonding. Professionals shared these while also citing additional priorities including breastfeeding rates, infant development, acceptability, reflective practice, and translating standards into practice.

**Conclusion:** The findings from this study provide insights into the needs and priorities of parents and professionals in relation to support for close and loving parent–infant relationships.

**References**

Braun V & Clarke V (2006). Using thematic analysis in psychology, Qualitative Research in Psychology, 3:2, 77‐101, DOI: 10.1191/1478088706qp063oa

UNICEF UK. (2013). The evidence and rationale for the Unicef UK Baby Friendly Initiative standards, UNICEF UK, London. Retrieved from: https://www.unicef.org.uk/wp-content/uploads/sites/2/2013/09/baby_friendly_evidence_rationale.pdf

Victora CG, Bahl R, Barros AJD, Franca GVA, Horton S, Krasevec J, Murch S, Sankar MJ, Walker N, Rollins NC (2016). Breastfeeding in the 21st century: epidemiology, mechanisms, and lifelong effect. Lancet 387 10017: 475–490.

## Healthcare Ethics: The Intersection of Practice and Culture

Darby Dickton^1^ and Jimi Francis^2^

^1^Foundation for Maternal Infant Lactation Knowledge, San Antonio, USA. ^2^University of Texas ‐ San Antonio, San Antonio, USA

**Background:** Although standards of practice and opinion largely dictate our clinical practices, there is occasionally gaps in cases where we are unable to find the correct course of action in a book or journal. In such cases that do not fit our typical algorithms, the field of ethics can guide us. Ethics can be viewed as an intersection between clinical practice and the law. While ethics is best based on logical assertions there will always be a cultural context that can shape a case and the unique difficulties which may confront you. We have witnessed the great influences of context in ethics in the recent international challenges experienced during the COVID pandemic, but such examples can be found on a much smaller scale in cases that may seem routine.

**Methods:** In this workshop, we will explore these concepts with an interactive review of 3 cases. The first case will explore the case of a 27‐year‐old vegan mother with a 9‐month‐old, malnourished infant, the current research available on deficiencies in this population and how to handle them while providing an appropriate level of protective care for the infant (Karcz & Królak‐Olejnik, 2021). The second will explore drug use during lactation in the case of a 22‐year‐old first‐time mother and the role of lactation consultants in discerning a potentially harmful situation and the legal responsibilities in addressing it (Jansson, 2009). The final case will look at the implications and options for mothers feeding or supplementing with formulas during severe formula shortages, as well as the ethical implication of milk banking and donor milk (Fang et al., 2021).

**Conclusion:** These cases will provide an opportunity to discuss the relevant laws and practices and how to implement them in ways that are comfortable for each level of provider.

**References**

Fang, M. T., Chatzixiros, E., Grummer‐Strawn, L., Engmann, C., Israel‐Ballard, K., Mansen, K., O'connor, D. L., Unger, S., Herson, M., Weaver, G., & Biller‐Andorno, N. (2021). Developing global guidance on human milk banking. Bulletin of the World Health Organization, 99(12), 892. https://doi.org/10.2471/BLT.21.286943

Jansson, L. M. (2009). ABM Clinical Protocol #21: Guidelines for Breastfeeding and the Drug‐Dependent Woman. Breastfeeding Medicine, 4(4), 225. https://doi.org/10.1089/BFM.2009.9987

Karcz, K., & Królak‐Olejnik, B. (2021). Vegan or vegetarian diet and breast milk composition ‐ a systematic review. Critical Reviews in Food Science and Nutrition, 61(7), 1081–1098. https://doi.org/10.1080/10408398.2020.1753650

## Developmental and nutritional changes in children with severe acute malnutrition treated with an improved ready‐to‐use therapeutic food and psychosocial support in Mwanza, Tanzania

Fredrick Cyprian^1^, Theresia Setebe^1^, Happyness Kunzi^1^, Jayne Webster^2^, Melissa Gladstone^3^, Erica Sanga^1^, Henrik Friis^4^, Adolfine Hokororo^5^, Maimuna Ahmed^5^, Suzanne Filteau^2^, George PrayGod^1^, Mette Frahm Olsen^4,6^

^1^National Institute for Medical Research (NIMR), Mwanza, Tanzania, United Republic of. ^2^Faculty of Epidemiology and Population Health, London School of Hygiene and Tropical Medicine, London, United Kingdom. ^3^University of Liverpool, Liverpool, United Kingdom. ^4^Dept of Nutrition, Exercise and Sports, University of Copenhagen, Copenhagen, Denmark. ^5^Bugando Medical Centre, Mwanza, Tanzania, United Republic of. ^6^Dept of Infectious Diseases, Rigshospitalet, Copenhagen, Copenhagen, Denmark

**Background:** More than 250 million children in low and middle‐income countries are at risk of not reaching their developmental potential due to poverty and malnutrition (Lu et al., 2016). Severe Acute Malnutrition (SAM) affects a large proportion of children in Sub‐Saharan Africa. Children with SAM are at higher risk of nutritional deficiencies including essential fatty acids (EFAs) which are important to brain development and these are not improved with standard RUTF.

Breakthroughs in ready‐to‐use therapeutic foods (RUTF) for management of SAM have played a major role in restoring physical health however, children's cognitive function may not recover without additional support (Grantham‐McGregor et al., 2013). Programs that support both nutrition and psychosocial stimulation benefit these outcomes (Hurley et al., 2016). Therefore, we conducted a trial development study to develop and pilot a novel RUTF (with increased EFAs) alongside a short, locally appropriate psychosocial intervention for children with SAM.

**Methods:** This study involves children aged 6 to 36 months in Mwanza, Tanzania. SAM children recruited to receive weekly enriched RUTF alongside psychosocial intervention for 8 weeks. Non‐malnourished children were recruited for reference levels of EFAs and child development. Outcomes included anthropometry and development using the Malawi Developmental Assessment Tool (MDAT), Family Care Indicators, and the Observation of Maternal Child Interaction.

**Results:** 82 children (52.4% girls) with SAM were recruited; mean age of 15.5 months (SD 6.9) and 88 non‐malnourished controls (47.7% girls); mean age 17.5 months (SD 7.9). At baseline, the global MDAT Z‐scores were lower in SAM children than non‐malnourished children −2.59 (−3.04, −2.15). After receiving the enriched RUTF and psychosocial stimulation global MDAT Z‐sores improved in children with SAM reducing the difference to non‐malnourished children with most effect observed on fine motor. Furthermore, levels of n‐3 fatty acids were also lower in SAM than controls at baseline −1.63 (95% CI −2.09; −1.18) FA% but significantly improved by 0.39 (95% CI 0.13; 0.66) FA% at the 8th week of intervention.

**Conclusion:** Improved RUTF alongside psychosocial stimulation showed to improve development in SAM children. Future randomized studies with a larger sample should be done to confirm these findings.

## Maternal and infant food insecurity in the UK. A problem hiding in plain sight?

Flora Douglas^1^, Emma MacIver^1^, Tracy Davis^2^

^1^Robert Gordon University, Aberdeen, United Kingdom. ^2^NHS Grampian, Aberdeen, United Kingdom

**Background:** Lone parents with children under five children are amongst the most food insecure in the UK (Cheong et al., 2021; Tobi et al., 2022). Yet maternal and infant food insecurity experience remains poorly understood in the UK. Drawing on findings from qualitative research conducted with parents of infants and young children, and early years health professionals, this paper highlights the hidden nature of poverty and related maternal and infant food insecurity and asks questions about how well this problem is recognised and understood within health care.

**Methods:** Two interview studies were conducted with 22 pregnant women and mothers with at least one child under five during 2020 and 2021 in north‐east Scotland. One study included interviews with 18 midwives, health visitors and family nurses (HCP). The studies investigated experiences of parenting on a low income, and, health professional support related to financial hardship challenges during pregnancy and early infancy. HP's perceptions of poverty within caseloads and experiences of raising financial issues during practice were also investigated. Data were thematic analysed using Grounded Theory principles.

**Findings:** Key parent themes included inadequate social security income co‐existing with restricted access to paid employment, anxieties around food and other resource provision for their children, going without food themselves and relying on charity or extended family for help with feeding. Fear of raising child protection concerns, shame and embarrassment, and exacerbating partner abuse prevented parents disclosing financial hardship and food insecurity to HPs. HPs themselves were aware of poverty within some households but not universally confident they could recognise the problem, were inhibited from raising the issue because of poverty stigma, and reported lacking time and knowledge to do so effectively.

**Conclusion:** Our findings point to economic, nutritional, and social vulnerability of lone parents that existed before the current cost of living crisis. As mothers continue to remain responsible for infant feeding either as food producers themselves or through infant formula procurement from commercial sources (Frank, 2018; Doonan, 2018) there is an urgent need to develop a better understanding of the nature and extent of maternal and infant food security in the UK to develop more effective public policy and health care practice.

**References**

Cheong, C. K., Dean, L., Dougall, I., Hinchliffe, S., Mirani, K., Vosnaki, K., & Wilson, V. (2020). The Scottish Health Survey 2018 edition; amended in February 2020: Volume 1, Main report.

Doonan, C. (2018). Rights for whom? Linking baby's right to eat with economic, social, and cultural rights for women. Canadian Food Studies/La Revue canadienne des études sur l'alimentation, 5(1), 8.

Frank, L. (2018). Finding formula: community‐based organizational responses to infant formula needs due to household food insecurity. Canadian Food Studies/La Revue canadienne des études sur l'alimentation, 5(1), 90‐112.

Tobi, R., Goudie, S., Taylor, A., Ralling, J., & Haydon, P. (2020). The Broken Plate 2020‐The State of the UK's Food System.

## Exploring the Impact of the Baby Friendly Hospital Initiative on Breastfeeding and Psychosocial Outcomes in the UK

Sophie Ellerington, Vicky Fallon, Joanne Harrold and Paul Christiansen

University of Liverpool, Liverpool, United Kingdom

**Background:** The benefits of breastfeeding to both mother and infant are well established, yet 30 years on from the launch of the Baby Friendly Hospital Initiative (BFHI), exclusive breastfeeding rates have not substantially increased in the UK. There is limited research into the benefit the initiative has upon breastfeeding, and upon wider outcomes in resource‐rich settings. This study aims to understand the relationship between BFHI accreditation and breastfeeding and maternal wellbeing in the UK.

**Methods:** 482 mothers of infants aged birth to 12 months completed an online survey which comprised infant feeding history and psychosocial measures. Mothers were also asked which trust they delivered at and this was coded according to BFHI accreditation information (full accreditation, partial accreditation, or no accreditation).

**Findings:** Results showed significant improvements in breastfeeding initiation within an hour of birth for women delivering in fully BFHI accredited hospitals, but no significant improvements for longer‐term breastfeeding outcomes and psychological wellbeing outcomes, compared to those delivering in hospitals with partial accreditation, and those delivering in hospitals with no accreditation.

**Conclusion:** This study reflects the findings of similar research into the impact of the BFHI in resource‐rich settings which suggest the BFHI may not improve breastfeeding rates (beyond the first hour of birth) or maternal wellbeing, both being underpinning principles of the initiative. Further research is needed to develop a clearer understanding of the UK BFHI and its impact and to ensure the design and implementation of the initiative delivers optimal care for all mothers and infants in the UK*.*

**References**

Fallon, V. M., Harrold, J. A., & Chisholm, A. (2018). The impact of the UK Baby Friendly Initiative on maternal and infant health outcomes: A mixed‐methods systematic review. *Maternal and Child Nutrition*, *15*(3).

Pérez‐Escamilla, R., Martinez, J. L., & Segura‐Pérez, S. (2016). Impact of the Baby‐friendly Hospital Initiative on breastfeeding and child health outcomes: A systematic review. *Maternal & Child Nutrition, 12*(3), 402–417. https://doi.org/10.1111/mcn.12294

Victora, C. G., Bahl, R., Barros, A. J. D., França, G. V. A., Horton, S., Krasevec, J., Murch, S., Sankar, M. J., Walker, N., & Rollins, N. C. (2016). Breastfeeding in the 21st century: epidemiology, mechanisms, and lifelong effect. *The Lancet*, *387*(10017), 475–490. https://doi.org/10.1016/S0140-6736(15)01024-7

## Factors affecting the implementation of effective interventions to support women to breastfeed: A systematic review and mixed methods synthesis

Albert Farre^1^, Shona Shinwell^1^, Sara Cumming^1^, Phyll Buchanan^2^, Anna Gavine^1^, Joyce Marshall^3^, Fiona Lynn^4^, Louise Wallace^5^, Alison McFadden^1^

^1^University of Dundee, Dundee, United Kingdom. ^2^Breastfeeding Network, Paisley, United Kingdom. ^3^University of Huddersfield, Huddersfield, United Kingdom. ^4^Queen's University Belfast, Belfast, United Kingdom. ^5^The Open University, Milton Keynes, United Kingdom

**Background:** Existing evidence suggests that when standalone breastfeeding support interventions are offered to women who choose to breastfeed, the duration and exclusivity of breastfeeding are likely to be increased (Gavine et al., 2022). However, many women in the UK and elsewhere continue to report that lack of adequate breastfeeding support results in them stopping breastfeeding earlier than planned. Therefore, one key research question is now to identify how known effective interventions can successfully be implemented in practice.

As part of the of the Action4Breastfeeding study, this review aimed to synthesise existing evidence on factors affecting the implementation of effective interventions identified in the updated Cochrane review on breastfeeding support for healthy women with healthy term babies (Gavine et al., 2022).

**Methods:** We systematically searched six bibliographic databases. Citation and reference searches of outcome papers from known effective interventions were also undertaken. No restrictions were applied on publication date and language. Articles reporting relevant qualitative and/or quantitative research were included. Quality appraisal was undertaken following study selection. Qualitative data were synthesised thematically (Thomas & Harden, 2008), and quantitative data narratively (Popay et al., 2006). A cross‐study synthesis (Kavanagh et al., 2012) integrated qualitative and quantitative findings.

**Findings:** Sixteen articles were included in the final synthesis. A range of eighteen factors affecting the implementation of effective interventions were identified and grouped around five broad types of implementation factors. The types of factors with a more widespread evidence base were related to the implementation process (particularly those relating to the ability to monitor and collect quantitative and qualitative feedback about the progress and quality of implementation) followed by those relating to the external context of the implementing organisation (particularly in terms of the organisation's knowledge of their women's/families’ breastfeeding support needs).

**Conclusion:** Available evidence on the range of factors known to have impacted implementation of effective breastfeeding support interventions can inform guidance for organisations to develop robust implementation strategies which may be more likely to ensure successful implementation of evidence into practice.

**References**

Gavine, A., Shinwell, S. C., Buchanan, P., Farre, A., Wade, A., Lynn, F., Marshall, J., Cumming, S. E., Dare, S., & McFadden, A. (2022). Support for healthy breastfeeding mothers with healthy term babies. Cochrane Database of Systematic Reviews, 10.

Kavanagh, J., Campbell, F., Harden, A., & Thomas, J. (2012). Mixed Methods Synthesis: A Worked Example. In K. Hannes & L. Craig (Eds.), Synthesizing Qualitative Research: Choosing the Right Approach (pp. 113–136). Wiley‐Blackwell.

Popay, J., Roberts, H., Sowden, A., Petticrew, M., Arai, L., Rodgers, M., Britten, N., Roen, K., & Duffy, S. (2006). Guidance on the conduct of narrative synthesis in systematic reviews: A product from the ESRC Methods Programme. Version 1. ESRC.

Thomas, J., & Harden, A. (2008). Methods for the thematic synthesis of qualitative research in systematic reviews. BMC Medical Research Methodology, 8(1), 45.

**Acknowledgments**

This study is funded by the NIHR Health and Social Care Delivery Research programme (NIHR 130955). The views expressed are those of the authors and not necessarily those of the NIHR or the Department of Health and Social Care.

## Macronutrient variance in Human Donor Milk: Implications for Fragile Infants

Jimi Francis^1^, Alex Nicholas^2^, Farjana Zaman^1^, Kelly McGlothen^3^, Elizabeth Brownell^3^, Darby Dickton^4^

^1^University of Texas, San Antonio, USA. ^2^University ofTexas, San Antonio, USA. ^3^University of Texas Health Science Center, San Antonio, USA. ^4^Foundation for MILK, San Antonio, USA

**Background:** Human milk is known to be the best source of nutrients for human infants, particularly preterm infants. (Lewis et al., 2017). When a mother's own milk is not available donor human milk is an alternative. (Ellsworth, Sturza, and Stanley 2021) Donor human milk (DHM) is categorized by caloric quantity, typically 20 calories per ounce and 24 calories per ounce. The DHM is prepared for distribution in lots or batches. (Moro et al., 2019). This study analyzed multiple Lots from one milk bank for macronutrient content to evaluate the range of macronutrient and energy content within and between Lot samples.

**Methods:** Frozen DHM was collected from a local hospital neonatal intensive care unit. Samples were chosen randomly from each Lot of either 20 kcal/ounce or 24 kcal/ounce. Each bottle was thawed and warmed to 40 degrees Celsius. Each sample was ultrasonically homogenized, and 3 3‐ml aliquots were removed from each bottle with 150 samples analyzed. The triplicate samples were analyzed using a MIRIS human milk analyzer. Descriptive statistics were calculated in Excel as well as multinominal regression, and ANOVA based on the expected energy density in relation to these macronutrient values. P values < 0.05 was considered statistically significant.

**Results:** Highly statistically significant differences were found within and between lots of the DHM. Protein content differed in 58% of the samples from the protein content listed on the label by more than 0.03 g/dl. The energy content differed from the label in 72% of the samples with an average difference of more than 3 units.

**Conclusions:** Differences have been reported between milk banks. (Jo et al., 2020). In this study, the differences found in macronutrient content between and within the Lots may pose a challenge for consistency of calorie intake in infants receiving only DHM if the energy content is assumed based on the labeling. Due to the differences found in this analysis, the nutrient intake may not meet the needs of an ill or fragile infant being fed DHM. Samples of DHM being fed to high‐risk infants should be regularly evaluated for macronutrient content to ensure the adequacy of the feeds.

**References**

Ellsworth, Lindsay, Julie Sturza, and Kate Stanley. 2021. “An Alternative to Mother's Own Milk: Maternal Awareness of Donor Human Milk and Milk Banks.” Journal of human lactation: official journal of International Lactation Consultant Association 37(1): 62–70. http://www.ncbi.nlm.nih.gov/pubmed/32735504 (March 29, 2021).

Jo, Diana B. et al., 2020. “Macronutrient Analysis of Donor Human Milk Labelled as 24 Kcal/Oz.” Journal of Perinatology 2020 40:4 40(4): 666–71. https://www.nature.com/articles/s41372-020-0624-2 (September 27, 2022).

Lewis, Erin D., Caroline Richard, Bodil M. Larsen, and Catherine J. Field. 2017. “The Importance of Human Milk for Immunity in Preterm Infants.” Clinics in Perinatology 44(1): 23–47.

Moro, Guido E. et al., 2019. “Processing of Donor Human Milk: Update and Recommendations From the European Milk Bank Association (EMBA).” Frontiers in pediatrics 7(FEB). https://pubmed.ncbi.nlm.nih.gov/30873395/ (September 27, 2022).

## Lactation Physiokinetics: Using new technology for a new perspective

Jimi Francis^1^, Darby Dickton^2^

^1^University of Texas, San Antonio, USA. ^2^Foundation for MILK, San Antonio, USA

**Background:** While breastfeeding initiation has increased, little progress has been made to increase the duration of exclusive breastfeeding of infants. (Survey, n.d.) New tools and techniques are needed to identify the specific problems and facilitate the development of personalized, precise, and targeted interventions when breastfeeding begins to fail. (Lee & Kelleher, 2016). The incomplete understanding of the physiokinetics of human milk transfer drives the current lack of evidence‐based interventions, resulting in only slight progress in increasing the number of months infants are breastfed. Despite the critical nature of breastfeeding, there is little understanding of the underlying physiological and biomechanical systems that lead to inadequate or complete failure of milk transfer.

**Methods:** Using Systems Biology (Breitling, 2010) and Systems Medicine (Saqi et al., 2016) as the framework, combined with Engineering design principles, a new tool using biosensors is being developed to describe and physically map milk movement from breast to infant's stomach, in real‐time, identifying points of regulation throughout the maternal‐infant lactation physiokinetics (MILK) system. The biosensor array, or BAR, is used to identify potential points of malfunction and map the features of the MILK system. The initial model of the MILK system included 8 maternal variables and 8 infant variables, most of which are measured synchronously using biosensors.

**Results:** All sensing elements of the MILK BAR are low‐cost, robust, and biocompatible to meet operational demands. Multiple sensor channels have been integrated for each use on mom and infant. Several regulatory factors have been identified and quantified that are important in milk transfer on both the maternal side and the infant side of the feeding system.

**Conclusions:** Utilizing technology (Lemmen et al., 2021) to develop ways to improve the outcomes through the prediction and prevention of milk transfer failure can lead to precision interventions for all moms choosing to breastfeed their infants, whether they are late‐preterm, premature, critically ill infants or a full‐term infant, it is crucial to recognize how to milk transfer works. A better understanding of how infants facilitate the movement of milk from breast to mouth using a systems approach will lead health care providers to earlier recognition of upsets in the finely balanced system that is lactation physiokinetics.

**References**

Breitling, R. (2010). What is systems biology? Frontiers in Physiology, 1 MAY, 159. https://doi.org/10.3389/FPHYS.2010.00009/BIBTEX

Lee, S., & Kelleher, S. L. (2016). Biological underpinnings of breastfeeding challenges: the role of genetics, diet, and environment on lactation physiology. American Journal of Physiology‐Endocrinology and Metabolism, 311(2), E405. https://doi.org/10.1152/AJPENDO.00495.2015

Lemmen, C., Simic, D., & Stock, S. (2021). A vision of future healthcare: Potential opportunities and risks of systems medicine from a citizen and patient perspective—results of a qualitative study. International Journal of Environmental Research and Public Health, 18(18). https://doi.org/10.3390/ijerph18189879

Saqi, M., Pellet, J., Roznovat, I., Mazein, A., Ballereau, S., De Meulder, B., & Auffray, C. (2016). Systems Medicine: The Future of Medical Genomics, Healthcare, and Wellness. Methods in Molecular Biology (Clifton, N.J.), 1386, 43–60. https://doi.org/10.1007/978-1-4939-3283-2_3

Survey, C. N. I. (n.d.). Breastfeeding Among U.S. Children Born 2012‐2019. Retrieved September 27, 2022, from: https://www.cdc.gov/breastfeeding/data/nis_data/results.html

## Infant feeding indicators of household food insecurity: Worries, feeding adjustments, and strategies to maximize food

Lesley Frank and Jane Francis

Acadia University, Wolfville, Canada

**Background:** Household food insecurity (HFI), compromised access to food due to financial constraint, disproportionately affects households with children. Qualitative research shows that HFI may lead mothers to initiate breastfeeding, but severe levels may undermine breastfeeding success (Frank, 2020). National Canadian data show that food insecure (FI) mothers stop exclusive breastfeeding earlier than those who are food secure (FS) (Orr, Dachner, Frank, & Tarasuk, 2018). However, little quantitative research investigates other infant feeding practices in relation to HFI and HFI measures do not reflect infant feeding compromises that may occur due to financial constraint.

**Methods:** Twenty‐five interviews with caregivers in Nova Scotia, Canada were conducted in 2020 to inform 36 potential indicators of infant food insecurity (IFI) across a variety of feeding methods and dimensions of FI (Radimer, Olson, & Campbell, 1990). A cross‐sectional survey was created including these indicators, other infant feeding questions, and the Canadian HFI module. The survey was tested in interviews (n = 20) and piloted online in 2022 to diverse caregivers with children under two (n = 1489). Survey responses were summarized using descriptive statistics and chi‐square tests were conducted.

**Results:** Among respondents, 31% experienced HFI; 34% provided only breastmilk to their infant, 10% only formula, and 57% some combination of breastmilk and formula. FI caregivers started breastfeeding (48%) and continued for as long as possible (64%), because of financial concerns. However, 26% fed formula while breastfeeding because of milk supply concerns due to not enough money for mother's food. FI caregivers worried about money to purchase formula (64%) and several qualitative and quantitative adjustments to feeding were captured, including running out of formula and not having money to buy more (13%). Fifty‐one percent of FI caregivers borrowed money to help feed their infant and 4% stole formula from retail outlets. Statistically significant differences (*p* < 0.05) were found between FI and FS caregivers across IFI indicators, but affirmative responses occurred even within FS households.

**Conclusion:** Multi‐dimensional differences related to infant feeding were found based on HFI status. Further research is warranted to assess how inclusion of IFI indicators might alter HFI assessment within families with infants, including households classified as FS.

**References**

1. Frank, L. (2020). Out of milk: Infant food insecurity in a rich nation. Vancouver: UBC Press.

2. Orr, S.K., Dachner, N., Frank, L., & Tarasuk, V. (2018). Relation between household food insecurity and breastfeeding in Canada. Canadian Medical Association Journal, 190(11), e312‐e319.

3. Radimer, K.L., Olson, C.M., Campbell, C.C. (1990). Development of indicators to assess hunger. The Journal of Nutrition, 120(suppl 11), 1544‐1548.

## A proactive infant feeding intervention delivered by health visitors to mothers in vulnerable positions: A qualitative evaluation study on mothers’ postpartum experiences

Marianne Frederiksen^1^, Virginia Schmied^2^, Charlotte Overgaard^1,3^

^1^Department of Health Science and Technology, University of Aalborg, Aalborg, Denmark. ^2^School of Nursing and Midwifery, Western Sydney University, Parramatta, Australia. ^3^The Unit of Health Promotion, University of Southern Denmark, Esbjerg, Denmark

Reflecting international guidelines, exclusive breastfeeding for 6 months is recommended in Denmark. However, as in other high‐income countries, social inequity exists in breastfeeding uptake and duration, and the establishment and maintenance of breastfeeding can be a challenging experience, particularly for mothers in vulnerable positions (Francis et al., 2020).

Consequently, a proactive infant feeding intervention has been implemented in Aalborg Municipality, Denmark, as part of community‐based project to reduce inequity in child health. The intervention consists of six telephone calls delivered by a health visitor during the first 6 months following birth. It targets mothers living with obesity, and/or mothers in a low socioeconomic position, ethnic minority background, and different psychosocial challenges. However, our previous research in the setting found that engaging with services can be an ambivalent experience for this group, and that the development of trusting relationships are central (Frederiksen et al., 2021).

To identify potential for development and generate in‐depth knowledge of the context and the user perspective (Leeming et al., 2017), the study explores how mothers in vulnerable positions experience receiving the intervention, and how contextual conditions shape these experiences. The study is designed as a qualitative evaluation study using serial semi‐structured interviews (Calman et al., 2013). Twenty women will be included, and three interviews with each will be conducted. As the project is in an early phase, this paper presents the protocol and preliminary findings.

**References**

Calman, L., Brunton, L., & Molassiotis, A. (2013). Developing longitudinal qualitative designs: lessons learned and recommendations for health services research. BMC Medical Research Methodology, 13(14).

Francis, J., Mildon, A., Stewart, S., Underhill, B., Tarasuk, V., Di Ruggiero, E., Sellen, D., & O'Connor, D. L. (2020). Vulnerable mothers’ experiences breastfeeding with an enhanced community lactation support program. Maternal and Child Nutrition, 16(3), 1–11.

Frederiksen, M. S., Schmied, V., & Overgaard, C. (2021). Supportive encounters during pregnancy and the postnatal period: An ethnographic study of care experiences of parents in a vulnerable position. Journal of Clinical Nursing, 30(15–16), 2386–2398.

Leeming, D., Marshall, J., & Locke, A. (2017). Understanding process and context in breastfeeding support interventions: The potential of qualitative research. Maternal and Child Nutrition, 13(4), 1–10.

## Association of breast feeding with healthful complementary feeding practices and the development and diversification of complementary feeding: data from Scottish Maternal and Infant Nutrition Survey

Ada L. Garcia, Jiali Huang, Charlotte Wright

Human Nutrition dept, University of Glasgow, Glasgow, United Kingdom

**Background:** We analysed data from the 2018 Scottish Maternal and Infant Nutrition Survey (MINS) for infants aged 7‐10 months to explore how breastfeeding relates to adherence to complementary feeding (CF) recommendations, diversification of the diet and development of feeding skills.

**Method:** Breastfeeding history and CF practices were assessed using a self‐completed questionnaire. Non‐healthful CF was age of first solids before 6 months, and consumption of sugar sweetened beverages (SSB), sweet or salty snack foods (treats), unmodified cow's milk or commercial baby foods. Diet was assessed via a short food frequency table. Feeding skills were the daily number of meals and snacks and self‐feeding. Sociodemographic characteristics were maternal age and Scottish Index of Multiple Deprivation (SIMD).

**Results:** Of the 2730 mothers participating, 20% never breastfed and 48% continued breastfeeding beyond 6 months; 47% started CF at ≥6 months. Treats were eaten daily by 26% babies, 14% ever had SSB and 4% unmodified cow's milk, while 39% had commercial baby foods most days; 82% had 3 meals daily; 24% had no regular snack, but 16% had 3 + . A median of 3 out of 9 possible food groups were eaten daily, the commonest being fruit (73%) and breakfast cereals (71%). Most babies (83%) self‐fed finger foods daily, while 30% self‐fed purees.

Compared to formula fed infants, babies breastfed ≥6 months were more likely to start solids ≥6 months (66% vs 37%) and less likely to give treats (15% vs 45%), SSBs (11% vs 20%) and commercial baby foods (31% vs 53%); all *p* < 0.001. These associations remained highly significant, even after adjustment for child and maternal age and SIMD. Despite starting solids later, infants breastfed ≥6 months ate the same number of food groups and meals as formula fed infants, were just as likely to self‐feed purees and were more likely to self‐feed finger foods daily (87% vs. 81% *p* < 0.001).

**Conclusions:** Those mothers who persist with breast feeding seem to have a broad commitment to healthful CF that does not simply reflect their age or underlying socioeconomic status. Breast feeding infants start solids later, but eat a similarly diverse diet and have similar feeding skills.

## Identifying effective breastfeeding support for healthy women and those with long‐term conditions

Anna Gavine^1^, Shona Shinwell^1^, Laura Hay^1^, Phyll Buchanan^2^, Albert Farre^1^, Angie Wade^3^, Fiona Lynn^4^, Joyce Marshall^5^, Sara Cumming^4^, Shadrach Dare^6^, Flavia Vasconcelos^1^, Alison McFadden^1^

^1^University of Dundee, Dundee, United Kingdom. ^2^Breastfeeding Network, NA, United Kingdom. ^3^UCL, London, United Kingdom. ^4^Queens University, Belfast, United Kingdom. ^5^Huddersfield University, Huddersfield, United Kingdom. ^6^GCU, Glasgow, United Kingdom

**Background:** Many women stop breastfeeding before they intended and report a lack of support from healthcare providers (Fox, McMullen, & Newburn, 2015). Moreover, women with multiple long‐term conditions may have additional difficulties breastfeeding (Scime, Patten, Tough & Chaput, 2022). The aim of this work is to identify effective interventions to support all women to breastfeed.

**Methods:** This presentation is comprised of two linked work packages of the Action4breastfeeding study. The first is an update of the Cochrane Review on Breastfeeding Support for healthy women with healthy term babies (Gavine et al., 2022). As this Cochrane review excludes women with long‐term conditions, an additional Systematic Review to identify effective interventions for women with long‐term conditions is currently underway.

**Results:** The updated Cochrane review identified 116 randomised controlled trials which involved 98,816 women and their babies. Interventions were grouped into ‘breastfeeding only’ interventions and ‘breastfeeding plus’ interventions which contain other aspects of maternal and newborn care. We found moderate certainty evidence that ‘breastfeeding only’ support helped reduce the number of women stopping any at exclusive breastfeeding at 4‐6 weeks, 3‐4 months and 6 months. For ‘breastfeeding plus’ the evidence is less certain but there was some evidence of a beneficial effect on exclusive breastfeeding. Meta‐regression identified that a schedule of 4‐8 contacts may help with exclusive breastfeeding. However, there were no differences in terms of who provides the support (i.e. peer or professional) or the mode of deliver (e.g. face‐to‐face, telephone, digital).

The Systematic Review on breastfeeding support for women with long‐term conditions has identified 20 studies meeting the inclusion criteria. Analysis is currently underway, and results will be presented.

**Conclusion:** For healthy women, organized support can help increase the duration and exclusivity of breastfeeding. Given the increase in prevalence of maternal long‐term conditions (NHS England, 2016), we need to better understand if support can also be effective for women with long‐term conditions. Further linked work is also on‐going to better understand how the effective interventions identified in these work packages can be implemented in an NHS setting.

**References**

Fox R, McMullen S, Newburn M. UK women's experiences of breastfeeding and additional breastfeeding support: a qualitative study of Baby Café services. BMC pregnancy and childbirth. 2015 Dec;15(1):1‐2.

Gavine A, Shinwell SC, Buchanan P, Farre A, Wade A, Lynn F, Marshall J, Cumming SE, Dare S, McFadden A. Support for healthy breastfeeding mothers with healthy term babies. Cochrane Database of Systematic Reviews 2022, Issue 10. Art. No.: CD001141. DOI: 10.1002/14651858. CD001141. pub6.

NHS England. Better Births. Improving Outcomes of Maternity Services in England. A 5 Year Forward View for Maternity Care. London; 2016.

Scime NV, Patten SB, Tough SC, Chaput KH. Maternal chronic disease and breastfeeding outcomes: a Canadian population‐based study. The Journal of Maternal‐Fetal & Neonatal Medicine. 2022 Mar 19;35(6):1148‐55.

**Acknowledgment**

This study is funded by the NIHR Health and Social Care Delivery Research programme (NIHR 130955). The views expressed are those of the authors and not necessarily those of the NIHR or the Department of Health and Social Care.

## It feels like an old‐fashioned telephone ringing in my breast: Autistic people's experiences of infant feeding and breastfeeding support

Aimee Grant^1^, Amy Brown^1^, Sara Jones^1^, Kathryn Williams^2^, Jennifer Leigh^3^, Eleanor Healer^1^

^1^Swansea University, Swansea, United Kingdom. ^2^Autistic UK CIC, Cardiff, United Kingdom. ^3^University of Kent, Kent, United Kingdom

**Background:** Around 2% of the population are Autistic (CDC, 2022) – including three of the research team. Autism is associated with differences in communication style and sensory processing, which can be intensely disabling without acceptance of differences and associated accommodations (Woods et al., 2018). We conducted a systematic review to identify the views and experiences of Autistic birthing parents in relation to infant feeding, identifying only eight peer‐reviewed articles (Grant, et al., 2022).

**Methods:** Online survey, with closed and open question, for UK‐based Autistic people who had been pregnant. Closed questions were analysed descriptively and open questions were analysed using inductive thematic analysis.

**Results:** 165 participants from the UK had given birth; breastfeeding and formula feeding experiences were reported by 140 and 84 participants respectively. Almost half of breastfeeding parents found it to be positive in some way, and almost 90% felt determined to breastfeed, despite any challenges encountered. A sizeable minority did not struggle with pain or the intensity of breastfeeding. Despite these positives, many reported struggles with the sensory aspects of breastfeeding, with around one‐third of mothers reporting struggling with: the intensity of the baby touching their body, unpleasant suckling sensations, and an uncomfortable or painful milk let‐down reflex. When it came to formula feeding, three‐quarters of participants noted that it was easy to understand how to prepare infant formula, although almost half were anxious about preparing formula safely. Participants reported a very high degree of unmet needs in relation to maternity care, with environments described as a sensory “nightmare”, health professionals often misunderstanding Autism and Autistic needs and participants feeling misunderstood, not listened to or unsupported. Three‐quarters of breastfeeding participants received some breastfeeding support in the postnatal period. Conversely, over three‐quarters of formula feeding participants noted that they had not received support with learning how to formula feed.

**Conclusion:** There is an urgent need for maternity services to meet the needs of Autistic birthing parents. Staff should expect Autistic people to present differently in relation to communication and breastfeeding‐related pain and sensory experiences; either experiencing these much more or much less than the average parent.

## Infant feeding policies and monitoring systems: A qualitative study across six European countries

Helen Gray^1^, Irena Zakarija‐Grković^2^, Adriano Cattaneo^3^, Charlene Vassallo^4^, Mariella Borg Buontempo^4^, Susanna Harutyunyan^5^, Maria Enrica Bettinelli^6^, Stefanie Rosin^7^

^1^Lactation Consultants of Great Britain, Bath, United Kingdom. ^2^University of Split School of Medicine, Split, Croatia. ^3^IBFAN Italia, Trieste, Italy. ^4^Health Promotion and Disease Prevention, Msida, Malta. ^5^Yerevan State Medical University, Yerevan, Armenia. ^6^University of Milan, Milan, Italy. ^7^Charité‐Universitätsmedizin Berlin, Berlin, Germany

**Background:** Implementation of the Global Strategy for Infant and Young Child Feeding varies widely among countries. Policymakers would benefit from insights into obstacles and enablers. This qualitative study explored some of the obstacles and enablers affecting the implementation of national infant and young child feeding policies and monitoring systems in six European countries (Croatia, Germany, Lithuania, Spain, Turkey and Ukraine).

**Methods:** The countries in the sample were chosen based on their scores on the two indicators for national policy and for monitoring systems in their respective World Breastfeeding Trends Initiative (WBTi) assessment reports. An online questionnaire was circulated to national WBTi teams, and inductive thematic analysis and a SWOT (Strengths, Weaknesses, Opportunities and Threats) analysis were conducted on the responses.

**Results:** Common enablers and strengths identified included: strong and continuous government commitment to infant and young child feeding, a proactive national breastfeeding authority, an effective national monitoring and evaluation system, implementation of the International Code of Marketing of Breastmilk Substitutes in national legislation, the integration of skilled breastfeeding supporters, the implementation of the Baby‐friendly Hospital Initiative, and positive cultural norms and traditions supporting optimal infant and young child feeding. In those cases where countries had faced extreme adversity, UNICEF took an important active role. The principal obstacles identified were weak government leadership, a lack of adequate national legislation on the International Code, and cultural norms which devalued women's care work including breastfeeding, while conflicts of interest and a strong influence of the baby feeding industry weakened and undermined breastfeeding policies and programmes everywhere.

**Conclusion:** Government commitment, adequate funding and strong measures for the protection of optimal infant and young child feeding are essential to the implementation of national policies and monitoring systems.

**References**

Global Breastfeeding Collective. (2021). Global breastfeeding scorecard 2021. Protecting breastfeeding through bold national actions during the Covid‐19 pandemic and beyond. https://www.globalbreastfeedingcollective.org/media/1591/file

Gray, H., Zakarija‐Grković, I., Cattaneo, A., Vassallo, C., Borg Buontempo, M., Harutyunyan, S., Bettinelli, M. E., Rosin, S. (2022). Infant feeding policies and monitoring systems: A qualitative study of European Countries. Maternal and Child Nutrition, 18(4), e13425. https://doi.org/10.1111/mcn.13425

Gupta, A., Suri, S., Dadhich, J. P., Trejos, M., & Nalubanga, B. (2019). The World Breastfeeding Trends initiative: Implementation of the global strategy for infant and young child feeding in 84 countries. Journal of Public Health Policy, 40(1), 35–65. https://doi.org/10.1057/s41271-018-0153-9

WHO. (2002). Global strategy for infant and young child feeding. World Health Organization, Geneva.

Zakarija‐Grković, I., Cattaneo, A., Bettinelli, M. E., Pilato, C., Vassallo, C., Borg Buontempo, M., Gray, H., Meynell, C., Wise, P., Harutyunyan, S., Rosin, S., Hemmelmayr, A., Šniukaitė‐Adner, D., Arendt, M., & Gupta, A. (2020). Are our babies off to a healthy start? The state of implementation of the global strategy for infant and young child feeding in Europe. International Breastfeeding Journal, 15(1), 51. https://doi.org/10.1186/s13006-020-00282-z

## Study protocol for *Positive feeding Of the Preterm infant (PoP‐study)*: A feasibility study of a developmentally supportive feeding strategy in the NICU

Linn Jahren Gustavsen^1,2^, Nina M. Kynø^1^, Edel J. Svendsen^1^, Bente S. Tandberg^2,3^, Claus A. Klingenberg^4,5^

^1^Oslo Metropolitan University, Oslo, Norway. ^2^Vestre Viken Hospital Trust, Drammen, Norway. ^3^Lovisenberg Diaconal University College, Oslo, Norway. ^4^University Hospital of North Norway, Tromso, Norway. ^5^The Artic University of Norway, Tromso, Norway

**Background:** For the immature preterm infant, family‐centered, developmentally supportive and neuroprotective care are established as standard of care in the modern NICUs (Coughlin et al., 2009; Gooding et al., 2011). Despite this, strategies to support feeding milestones in preterm infants, is still an area with widely use of experience‐based knowledge. Breastfeeding is challenging for the preterm infant and the prevalence of problematic feeding is high (Pados et al., 2021). Randomized, controlled studies regarding developmentally supporting strategies for feeding report the quality of evidence to be low and risk for bias as high, though single studies report positive outcomes (Watson & McGuire, 2016). NICUs are using elements of developmentally supportive strategies, but there is lack of systematically implementation (McFadden et al., 2021). The aim of this study is to develop the intervention *Positive feeding of the Preterm infant* (PoP) and test the feasibility to implement it in a single room NICU.

**Methods:** The study will be conducted as a mixed method feasibility study in a level 3a single room NICU, divided into three substudies. Substudy 1 will develop a prototype of the Pop‐intervention by a) explore problematic feeding in the preterm infant using the validated NeoEat questionnaire, b) review of literature, and c) arranging workshops for parents, nurses, and physicians. The prototype will be utilized in subsstudy 2, a feasibility study to test the implementation of the intervention over a period of twelve months assessing recruitment, retention, data collection, resources needed, acceptability and suitability, and evaluation of the intervention. Substudy 3 will explore the parents experiences of the intervention, with qualitative interviews of the participants from the feasibility study.

**Conclusion:** The study will contribute to understand the facilitators and the barriers for implementing a developmentally supportive feeding strategy in the NICU and present preliminary results of the PoP‐intervention.

**References**

Coughlin, M., Gibbins, S., & Hoath, S. (2009). Core measures for developmentally supportive care in neonatal intensive care units: theory, precedence and practice. Journal of advanced nursing, 65(10), 2239‐2248.

Gooding, J. S., Cooper, L. G., Blaine, A. I., Franck, L. S., Howse, J. L., & Berns, S. D. (2011). Family support and family‐centered care in the neonatal intensive care unit: origins, advances, impact. Seminars in perinatology, 35(1), 20‐28. https://doi.org/10.1053/j.semperi.2010.10.004

McFadden, A., Fitzpatrick, B., Shinwell, S., Tosh, K., Donnan, P., Wallace, L. M., Johnson, E., MacGillivray, S., Gavine, A., & Farre, A. (2021). Cue‐based versus scheduled feeding for preterm infants transitioning from tube to oral feeding: the Cubs mixed‐methods feasibility study. Health Technology Assessment, 25(74).

Pados, B. F., Hill, R. R., Yamasaki, J. T., Litt, J. S., & Lee, C. S. (2021). Prevalence of problematic feeding in young children born prematurely: a meta‐analysis. BMC pediatrics, 21(1), 1‐15.

Watson, J., & McGuire, W. (2016). Responsive versus scheduled feeding for preterm infants. Cochrane Database Syst Rev(8). https://doi.org/10.1002/14651858.CD005255.pub5

## Infant colic and touch‐based treatments

Leena Hannula^1^, Sandra Rinne^1^, Tiina Väänänen^2^

^1^Metropolia University of Applied Sciences, Helsinki, Finland. ^2^University of Tampere, Tampere, Finland

**Background:** Infantile colic is a common cause for distress among parents. Several studies have indicated that manual treatments (osteopathy, reflexology or affective touch) might be beneficial in reducing infants’ colic symptoms. Individual studies have evaluated the effect of a single manual treatment versus treating control group with placebo or sham treatment. However, there are no prior intervention studies that would evaluate and compare the effect of different manual treatments on infants with colic symptoms. The objective of this study is to compare the effects of three types of touch‐based interventions, osteopathic manual treatment, reflexology and affective touch, with standard care with normal counselling in reducing infantile colic symptoms as reported by their parents as well as self‐reported stress and well‐being of the parents.

**Methods:** The infants were pseudo‐randomised in receiving reflexology, osteopathy and affective touch treatments. For osteopathy, reflexology and caring touch groups there were three treatment sessions within a 3‐week period. In every treatment intervention group, each hands‐on treatment lasted 20‐30 min. However, an hour was set aside for each treatment session, so that the treatment could be carried out without haste.

**Results:** In total, 143 infants (< 10 weeks) and parents (36 osteopathy, 40 reflexology, 40 affective touch and 27 control group) met the inclusion criteria. In the end, 101 families completed all the follow‐up questionnaires. The results were analysed statistically. Average treatment effect was examined with time*group interaction, where osteopathy and reflexology where categorised in one group. Mixed model estimation was used to account for the hierarchical nature of the data. Three time points were available for the control design (T1, T3 and T4) and a linear treatment effect was fitted. Infant age and gender as well as their interactions with time were controlled for as covariates.

Only the contrast Osteopathy/Reflexology versus Control yielded statistically significant differences (one‐tailed p‐value, no correction for multiple comparisons): reported crying time, last 24 h (*p* = 0.0425) and reported sleep, last week (*p* = 0.049).

**Conclusions:** Other results related to colic symptoms, parental and postpartum stress and infant feeding in the intervention and control groups will be discussed. The feedback from the participating parents was very positive and no adverse treatment effects were reported.

## Managing connection and disconnection: Relationships as the centre of Lactation Consultant care

Jennifer Hocking^1,2^, Virginia Schmied^3^

^1^Australian Catholic University, Melbourne, Australia. ^2^Western Sydney University, University, Australia. ^3^Western Sydney University, Sydney, Australia

**Background:** Australia has the most International Board Certified Lactation Consultants per capita in the world. Despite this there is little published research that examines or describes the clinical practice of this professional group whose key clinical role is supporting women to breastfeed (Carroll and Reiger, 2005).

**Methods:** A focused ethnographic study was carried out by the lead author across a range of practice settings: hospital, community and private practice. Participant observation was carried out in each practice context and clinicians were interviewed about their perception of their role. Theoretical frameworks included Merleau‐Ponty's theory of embodiment and theories of care (Hamington 2004, Noddings 2013).

**Findings:** Care influenced by relationships was the overarching theme to arise from the fieldwork and interviews. Lactation Consultants in the study based their care on reciprocal and participatory relationships with women. LCs used strategies to achieve this that included consistent kindness and gentleness in their first contact with women, invitations for women to tell their own story of birth and breastfeeding, and prioritizing as much continuity of care as the model of care allowed. While LCs were often seen by other health professionals to be the ‘fixers’ of breastfeeding problems, they saw their work as more than this.

**Conclusion:** Relational care offered by LCs acknowledges the mother‐baby dyad as the centre of the breastfeeding relationship. Similarly, LCs worked in a way that promoted this relational bond.

**Reference**

Carroll, K. and K. Reiger (2005). “Fluid experts: lactation consultants as postmodern professional specialists.” *Health Sociology Review* 14(2): 101‐110.

Hamington, M. (2004). Merleau‐Ponty and embodied epistemology: caring habits and caring knowledge. Embodied Care: Jane Addams, Maurice Merleau‐Ponty, and Feminist Ethics. Chicago, University of Illinois Press: 38‐60.

Noddings, N. (2013). Caring: a relational approach to ethics and moral education. Los Angeles, University of California Press.

## Baby Food Marketing in the UK, what do caregivers see and perceive?

Grace Hollinrake, Amy Brown and Sofia Komninou

Swansea University, Swansea, United Kingdom

**Background:** Increasingly research is examining how taste preferences and eating behaviours are shaped during infancy. One important period is when infants are introduced to complementary foods. There is a large and growing market of baby food products (BFP), including purées and baby snacks, marketed as suitable for complementary feeding. However, concerns have been raised over content and taste with many having high levels of sugar, despite regulations. Although BFP variety and composition have been examined in research, caregivers’ perceptions of them and reasons for choosing specific products have not.

**Method:** A self‐report, online questionnaire for parents/carers of infants aged 4‐12 months, who had introduced solid foods was used. Questions examined frequency and reason for use of different BFP products and brands.

**Results:** 271 parents responded with 173 using BFP. The main reasons for using BFP were convenience, time saving, and acceptance/enjoyment. Conversely, parents who did not use BFP preferred to give homemade foods earlier, disliking the content of BFP and being distrustful of them.

Notably, snack products were used more frequently than purées, and were often used for convenience or as a distraction to settle infant behaviour rather than for nutritional content. Some caregivers viewed them as a safer and less messy way to allow babies to self‐feed and experience different textures. BFP snack foods were seen as healthier than adult snack foods.

Specific products were often chosen due to perception of ingredients. This included viewing products with multiple or more unusual vegetables in them as healthier, or misleading ‘health halo’ statements such as ‘organic’, ‘no added salt’ or ‘no additives’ driving choice. Certain products such as pouches were viewed as convenient as babies could self‐feed. Finally, some BFP were used as the brand was perceived to be trustworthy and used the ‘best’ ingredients.

**Conclusion:** Much like with the infant formula industry, marketing and promotional strategies are affecting parents’ decision making when it comes to choosing foods to offer their baby. Often these are misleading and distract from problematic elements such as high sugar content. Parents deserve accurate information about the content and impact of BFP.

**References**

Brown, A., & Rowan, H. (2016). Maternal and infant factors associated with reasons for introducing solid foods. *Maternal & Child Nutrition, 12*(3), 500–515.

Crawley, H., & Westland, S. (2017). Baby foods in the UK: A review of commercially produced jars and pouches of baby foods marketed in the UK. *First Steps Nutrition Trust*. https://static1.squarespace.com/static/59f75004f09ca48694070f3b/t/5a93f8855229b264ff6086/1519646858256/Baby_Food_in_the_UK+_2017.pdf

Garcia, A. L., Looby, S., McLean‐Guthrie, K., & Parrett, A. (2019). An Exploration of Complementary Feeding Practices, Information Needs and Sources. *International Journal of Environmental Research and Public Health, 16*(22).

Garcia, A. L., Curtin, L., Ronquillo, J. D., Parrett, A., & Wright, C. M. (2020b). Changes in the UK baby food market surveyed in 2013 and 2019: The rise of baby snacks and sweet/savoury foods. *Archives of Disease in Childhood, 105*(12), 1162–1166.

Hutchinson, J., Rippin, H., Threapleton, D., Jewell, J., Kanamäe, H., Salupuu, K., Caroli, M., Antignani, A., Pace, L., Vassallo, C., Lande, B., Hildonen, C., Rito, A. I., Santos, M., Gabrijelcic Blenkus, M., Sarkadi‐Nagy, E., Erdei, G., Cade, J. E., & Breda, J. (2020). High sugar content of European commercial baby foods and proposed updates to existing recommendations. *Maternal & Child Nutrition, 17*(1).

## Breastfeeding Sick Children in Hospital: Exploring mothers’ experiences of breastfeeding a medically complex child in the paediatric setting.

Lyndsey Hookway, Amy Brown and Aimee Grant

Swansea University, Swansea, United Kingdom

**Background:** There is a paucity of literature exploring the challenges of breastfeeding sick children within paediatrics. Previous research has largely focused on single conditions and hospitals which limits understanding of the challenges in this population (Hookway et al., 2021). Although evidence suggests that current healthcare professional training in paediatrics is often inadequate (McLaughlin and Young, 2011; Brewer, 2012; Colaceci et al., 2017), it is unclear where the specific training and knowledge gaps are.

**Methods:** This qualitative study of UK mothers explored the challenges of breastfeeding sick children in paediatrics. Mothers were invited via an online screening questionnaire to participate in an interview study. From 504 respondents, a clinically and geographically diverse sample of 30 mothers of children aged 2‐36 months with various conditions and demographic backgrounds was chosen.

**Results:** A reflexive thematic analysis was undertaken, and seven themes generated. There were numerous ways in which breastfeeding was more challenging during illness, including previously unreported impacts such as high fluid losses, severe fluid restriction with high caloric need, iatrogenic withdrawal, neurological irritability and changes to breastfeeding behaviour. Mothers described breastfeeding as an invaluable act of emotional and immunological nurture that was mutually meaningful.

There were many complex psychological challenges related to breastfeeding a sick child, such as guilt, disempowerment, and trauma. Wider struggles such as staff resistance to bed‐sharing, lack of food and inadequate breast pump provision were stressful and made breastfeeding more challenging. These struggles often existed against a backdrop of staff ambivalence, lack of support or encouragement and inability to provide accurate breastfeeding information that was fit for purpose.

**Conclusions:** There are numerous challenges of breastfeeding and responsively parenting sick children in paediatrics, and these also impacted maternal mental health. Staff skill and knowledge gaps were widespread, and the clinical environment was not always conducive to supporting breastfeeding. This study highlights strengths in clinical care and provides insight into what measures are perceived as supportive by mothers. It also highlights areas for improvement, which may inform more nuanced paediatric breastfeeding standards.

## Text message conversations between peer supporters and women to deliver infant feeding support using behaviour change techniques: A qualitative analysis

Jenny Ingram^1^, Olivia Knox^1^, Denise Parker^1^, Debbie Johnson^1^, Stephan Dombrowski^2^, Gill Thomson^3^, Joanne Clarke^4^, Pat Hoddinott^5^, Kate Jolly^4^

^1^University of Bristol, Bristol, United Kingdom. ^2^University of New Brunswick, Fredericton, Canada. ^3^University of Central Lancashire, Preston, United Kingdom. ^4^University of Birmingham, Birmingham, United Kingdom. ^5^University of Stirling, Stirling, United Kingdom

**Background:** An infant feeding assets‐based peer support intervention (ABA) was developed to include components associated with higher breastfeeding rates: woman‐centred proactive support, spanning the antenatal and postnatal period, and continuity of peer feeding helpers (1). The intervention was delivered through face‐to‐face, telephone and text message by peer supporters (with training in ABA) called Infant Feeding Helpers (IFHs) from 30‐weeks’ gestation until 5‐months postnatal. It draws on Behaviour Change Techniques (BCTs) in line with recommendations for theory‐based interventions (2). The specific BCTs underpinning ABA included providing ‘social support’ and ‘changing the social environment’.

**Objective:** To analyse text message conversations between IFHs and new mothers to understand how peer support can influence and support women's feeding experiences.

**Design:** Qualitative thematic analysis of text messages using both inductive and deductive approaches. Deductive analysis was used to code if BCTs were employed by IFHs in their interactions with women. BCTs coded in text messages were then compared with those from antenatal meeting recordings and documented in interview transcripts.18 primiparous women and 7 Infant Feeding Helpers from one community site in South‐West England were included.

**Findings:** Three key themes were identified in the18 text message conversations: ‘breastfeeding challenges’, ‘mother‐centred conversations’, and ‘emotional and practical support’. The core BCTs of ‘social support’ and ‘changing the social environment’ were found at least once in all text message conversations. Meanwhile, ‘instruction to perform the behaviour’ was used in over 50% of conversations. Generally, the use of BCTs was greatest between birth and 2 weeks during a period of daily texts when women reported many feeding challenges. The number and range of BCTs used in text messages were similar to those documented in audio‐recorded meetings and interview accounts.

**Conclusions:** Infant Feeding Helpers were able to provide engaging and successful breastfeeding peer support through text messages. Messaging was shown to be an appropriate and accessible method of delivering BCTs focussing on ‘social support’ and ‘changing the social environment’. Peer supporters delivering BCTs via text messages is acceptable and appropriate to use if in‐person support may be limited in future.

**References**

1. Clarke JL et al., The ABA intervention for improving breastfeeding initiation and continuation: feasibility study results. Maternal & Child Nutrition. 2020;16(1):e12907.

2. Michie S et al., The behaviour change wheel: a new method for characterising and designing behaviour change interventions. Implementation Science 2011;6:42.

## Supporting women with learning disabilities in making infant feeding decisions

Clare Johnson^1^, Douglass Emma^1^, Geraldine Lucas^1^, Sally Dowling^1,2^

^1^University of the West of England, Bristol, United Kingdom. ^2^University of Bristol, Bristol, United Kingdom

**Background:** Women with learning disabilities are less likely to breastfeed than other women. During pregnancy they are supported by a range of professionals and there may be competing concerns. Preparing to feed a baby involves learning new skills and information. We were interested in how these women are supported to make infant feeding choices, what resources are available and how they are used; little is known about the acceptability of existing resources/visual images. We are an interdisciplinary team: learning disability nursing, visual arts, midwifery and public health.

**Methods:** This was a three‐stage project:

1. A scoping review (Johnson et al., 2022). Using a systematic process we searched fourteen electronic databases; nine items were identified. We conducted a synthesis of the grey literature and a thematic analysis of published literature.
2. Qualitative interviews with seven UK health professionals (Dowling et al., 2022), and a thematic analysis.
3. A focus group with women with learning disabilities. Photo elicitation was used to gather views and perspectives. Thematic analysis and a critical visual analysis were undertaken.

**Findings:** Women with learning disabilities need tailored support, including accessible resources. There is little evaluation of the imagery used in existing resources. Health professionals highlighted the importance of unconditional, positive regard, being part of the support network, and the need for an individualised approach. In talking to women with learning disabilities we identified that there is a need for resources, avoiding the one‐size‐fits‐all approach, recognising and embracing differences in terms of understanding, visual literacy and cultural taste.

**Conclusion:** Our findings suggest that women with learning disabilities can make and put into practice infant feeding decisions if they have access to the right support at the right time. Further development of a suite of resources is needed.

**References**

Dowling, S., Douglass, E., Lucas, G., & Johnson, C. (2022). Supporting women with learning disabilities in infant feeding decisions: UK healthcare professionals’ experiences. Maternal & Child Nutrition, e13432. https://doi.org/10.1111/mcn.13432

Johnson, C., Douglass, E., Lucas, G., & Dowling, S. (2022). Supporting women with learning disabilities in infant feeding decisions: a scoping review. Maternal and Child Nutrition, 18. https://doi.org/10.1111/mcn.13318

## Finding the formula: Using community science to explore the real‐world experiences of formula preparation

Sara Jones^1^, Rebecca Ellis^1^, Amy Brown^1^, Vicky Sibson^2^, Emma Yhnell^3^, Sharon Breward^4^, Phyll Buchanan^5^, Aimee Grant^1^

^1^Swansea University, Swansea, United Kingdom. ^2^First Steps Nutrition Trust, London, United Kingdom. ^3^Cardiff University, Cardiff, United Kingdom. ^4^Betsi Cadwaldr University Health Board, Bangor, United Kingdom. ^5^The Breastfeeding Network, Edinburgh, United Kingdom

**Background:** In the UK 85% of babies receive infant formula by 3 months of age (McAndrew et al., 2012). Gastrointestinal infections in infancy have been attributed to formula feeding (Renfrew et al., 2012). This is partly because fully formula fed babies do not receive the gut and immune protective properties of breastmilk, however, the preparation of infant formula also plays a role. Powdered infant formula (PIF) is not sterile and can contain harmful bacteria, reconstituted formula can grow bacteria if made in advance for later use, and bottle‐feeding equipment can easily be contaminated (WHO, 2007). Therefore, to avoid sickness, the NHS recommends making up formula using water which is hot enough to kill pathogens (70°C or greater), making bottles one at a time for immediate use and ensuring hands, surfaces and equipment are clean (NHS, 2019). However previous research has indicated that parents often do not feel confident to do this or rely on outdated guidance (Brown, Jones & Evans, 2020). Our project, co designed with parents, aimed to explore parents’ usual PIF preparation practices, and was funded by the Food Standards Agency as part of a wider call to explore the application of community science research to food safety challenges.

**Methods:** 151 parents completed an at‐home experiment and online research diary. Parents were asked about their usual PIF preparation practices. They also received thermometers and instructions and were asked to measure the temperature of the water used to make PIF.

**Results:** We found several areas where parents practices did not always align with NHS advice. For example, around a fifth of parents (20.1%) reported not always making bottles one at a time for immediate use. Many parents (53.6%) did not always wash their hands, nor always clean and disinfect bottle preparation surfaces (66.9%). Time pressures and impracticability were barriers to following the NHS advice. The average water temperature reported was 69.6°C (SD12.2, range 36.5°C to 99.5°C). Of concern, 54.3% of parents reported temperatures below 70°C.

**Conclusion:** The research highlights a need for more parental support around preparing infant formula safely. Further investigation is needed.

**References**

Brown, A., Jones, S. & Evans, E. (2020) Marketing of infant milks in the UK: what do parents see and believe? https://static1.squarespace.com/static/59f75004f09ca48694070f3b/t/5f93548ed1d6ae350aa8050c/1603490967170/Marketing_of_infant_milk_in_the_UK-what_do_parents_see_and_believe_finala.pdf

McAndrew, F., Thompson, J., Fellows, L., Large, A., Speed, M. & Renfrew, M. (2012). Infant feeding survey 2010. Health and Social Care Information Centre. https://sp.ukdataservice.ac.uk/doc/7281/mrdoc/pdf/7281_ifs-uk-2010_report.pdf

NHS (2019, September 24). How to make up baby formula. https://www.nhs.uk/conditions/baby/breastfeeding-and-bottle-feeding/bottle-feeding/making-up-baby-formula/

Renfrew, M., Pokhrel, S., Quigley, M., McCormick, F., Fox‐Rushby, J., Dodds, R. et al., (2012). Preventing Disease and saving resources: the potential contribution of increasing breastfeeding rates in the UK. https://www.unicef.org.uk/wp-content/uploads/sites/2/2012/11/Preventing_disease_saving_resources.pdf

WHO (2007) Safe preparation storage and handling of powdered infant formula – Guidelines. Available from: https://apps.who.int/iris/bitstream/handle/10665/43659/9789241595414_eng.pdf?sequence=1&isAllowed=y

## Breastfeeding of late preterm twins: Initiation, duration, and experiences of mothers

Rakel Jonsdottir^1,2^, Renée Flacking^3^, Helga Jonsdottir^2^

^1^Neonatal Intensive Care Unit, Landspitali – The National University Hospital of Iceland, Reykjavik, Iceland. ^2^Faculty of Nursing, School of Health Sciences, University of Iceland, Reykjavik, Iceland. ^3^School of Health and Welfare, Dalarna University, Falun, Sweden

**Background:** Given the known short‐ and long‐term consequences of late preterm (LPT) and twin births and the maternal and infant health benefits of breastfeeding/breastmilk (Victora et al., 2016; Ward & Caughey, 2021), breastfeeding is of particular importance for LPT infants. Hence, it is a concern that twins and LPT infants have low rates of breastfeeding (Ostlund, Nordstrom, Dykes, & Flacking, 2010; Porta et al., 2019). The objectives of this study were to describe the initiation and duration of any and exclusive breastfeeding at the breast for mothers of LPT twins and term twins during the first 4 months and to explore the breastfeeding experiences of mothers.

**Methods:** A sequential explanatory mixed‐method design was used (Ivankova, Creswell, & Stick, 2006) encompassed quantitative data from 44 mothers of LPT twins and mothers of 82 term twins and qualitative data from 14 mothers of LPT twins. Analysis included descriptive statistics of quantitative data and deductive content analysis of the qualitative data, followed by condensation and synthesis.

**Results:** The findings showed that almost all mothers of LPT twins initiated breastfeeding, and that the breastfeeding rate during the first month was the same as in mothers of term twins. However, after the first month, more mothers of LPT twins ceased breastfeeding, leading to large differences in the breastfeeding rate at 4 months of twins’ age compared to term twins. Key factors influencing mothers’ experiences and decisions were their infants’ immature breastfeeding behaviors requiring them to express breast milk alongside breastfeeding, the burden of following task‐oriented feeding regimes, and the lack of guidance from healthcare professionals. As a result, mothers started to question the worth of their breastfeeding efforts, leading to changes in breastfeeding management with diverse results. Support from fathers and grandparents positively influenced sustained breastfeeding.

**Conclusions:** Mothers of LPT twins aspire and want to breastfeed but they face many challenges in breastfeeding during the first month, leading to more LPT twins’ mothers than term twins’ mothers ceasing breastfeeding during the following months. To promote and safeguard breastfeeding in this vulnerable group, care must be distinguished from routine term infant services, and healthcare professionals need to receive fitting/suitable education and training.

**References**

Ivankova, N.V., Creswell, J.W., Stick, S.L. (2006). Using mixed‐methods sequential explanatory design: From theory to practice. Field Methods,18:3‐20.

Ostlund, A., Nordstrom, M., Dykes, F., and Flacking, R. (2010). Breastfeeding in preterm and term twins ‐ maternal factors associated with early cessation: A population‐based study. Journal of Human Lactation, 26:235‐41.

Porta, R., Capdevila, E., Botet, F., Ginovart, G., Moliner, E., Nicolas, M., et al., (2019). Breastfeeding disparities between multiples and singletons by NICU discharge. Nutrients, 11:(9):2191.

Victora, C.G., Bahl, R., Barros, A.J., Franca, G.V., Horton, S., Krasevec, J., et al., (2016). Breastfeeding in the 21st century: Epidemiology, mechanisms, and lifelong effect. Lancet, 387:475‐90.

Ward, C., & Caughey, A. B. (2021). Late preterm births: neonatal mortality and morbidity in twins versus singletons. The journal of maternal‐fetal & neonatal medicine: the official journal of the European Association of Perinatal Medicine, the Federation of Asia and Oceania Perinatal Societies, the International Society of Perinatal Obstetricians, 1–6. Advance online publication. https://doi.org/10.1080/14767058.2021.1939303

## Changing culture and practice to promote and enhance exclusive breastfeeding in a formula focused environment

Patricia Leahy‐Warren^1^, Sandra O'Connor^2^, Michelle O'Driscoll^1^, Virginia Schmied^3^, Elaine Lehane^1^, Rhona O'Connell^1^, Margaret Murphy^1^, Catherine Buckley^4^, Helen Mulcahy^1^

^1^School of Nursing & Midwifery, University College Cork, Cork, Ireland. ^2^University Hospital Kerry, Kerry, Ireland. ^3^School of Nursing & Midwifery, Western Sydney University, Sydney, Australia. ^4^Northridge House Education and Research Centre, St. Lukes Home Castle Road, Mahon, Cork, Ireland

**Background:** The rates of exclusive breastfeeding in Ireland as the lowest in Europe. Having an enabling environment for breastfeeding encompasses an insight into mother and infant characteristics, as well as the barriers and facilitators of a variety of settings (health systems, community, family), that are all rooted within certain sociocultural and market systems (Gallegos et al., 2020).

**Methods:** Using an implementation science research design underpinned by the integrated i‐PARIHS framework (Harvey & Kitson, 2015), the focus of this paper is to present the baseline situational assessment to determine the existing policies, practices, and culture in relation to exclusive breastfeeding. Following ethical approval, data were collected from four clinical sites: two GP practices, a maternity hospital, and public health nursing services. Data instruments included: Physical Environmental Assessment Checklist; Policy audits using the BFHI checklist; Staff survey included demographics and validated instruments measuring readiness for change and the likelihood of successful implementation of change.

**Findings:** Findings from the environmental assessment differentiated between the GP practices, the maternity and public health nursing services in terms of parking, specific designated feeding areas, seating and signage on breastfeeding. With regard to the BFHI policy checklist, similarities and differences were found between community sites and maternity services, particularly around education and training of staff and compliance with the International Code of Marketing of Breastmilk Substitutes. Eighty‐five staff responses were received, the majority were female and in the 40‐49 age group. There was an openness and positive attitudes to change. Similarly, high scores were reported in the likelihood of successful implementation of change.

**Conclusion:** This situational analysis provides a very positive starting point for the facilitation of the environmental, culture and policy changes required to enhance successful breastfeeding in study settings.

**References**

Gallegos, D., Parkinson, J., (2020). Understanding breastfeeding behaviours: a cross‐sectional analysis of associated factors in Ireland, the United Kingdom and Australia. *International breastfeeding journal*, 15(1), 1‐12.

Harvey, G. Kitson, A., (2015). PARIHS revisited: from heuristic to integrated framework for the successful implementation of knowledge into practice. *Implementation science*, 11(1), pp. 1‐13.

## Systematic Review and Meta‐analysis of the effect of relaxation interventions on breastmilk quantity and maternal mental health

Ilana Levene^1^, Nurul Husna Mohd Shukri^2^, Frances O'Brien^3^, Maria Quigley^1^, Mary Fewtrell^4^

^1^National Perinatal Epidemiology Unit, University of Oxford, Oxford, United Kingdom. ^2^Universiti Putra Malaysia, Serdang, Malaysia. ^3^Newborn Care Unit, Oxford, United Kingdom. ^4^Institute for Child Health, University College London, London, United Kingdom

**Background:** Human milk feeding is an important public health goal (Victora et al., 2016). Rates of human milk feeding are low in the UK and variable worldwide. Maternal stress could impair lactation, through hormonal (Stuebe et al., 2012), neuronal or behavioural pathways (Mohd Shukri et al., 2018). This study aims to analyse the effect of relaxation interventions on lactation outcomes.

**Methods:** The question was defined as: “in lactating women does relaxation therapy, compared to standard care, improve milk quantity and maternal mental health?” The systematic search included major medical and psychological databases, using keywords and MeSH terms. Only randomised studies with interventions designed to achieve relaxation through reduction in mind‐stress were included. Fixed‐effects meta‐analysis was used to estimate a standardised mean difference (SMD) where units of measurement varied between studies. Standardised effect sizes were converted to representative clinical situations to assess clinical significance.

**Results:** The search took place in August 2022. Twelve studies are included, with data from 1533 lactating parents. Interventions included lactation‐specific guided relaxation, music, mindfulness and yoga. Seven studies measured milk quantity, either expressed or via direct breastfeeding. There was an outlier study accounting for all the statistical heterogeneity seen. The summary SMD was 0.75 including the outlier study and 0.53 excluding it. An interpretation of the size of this summary effect is an increase in daily breastmilk yield by 225 ml or 160 ml respectively, which is clinically significant. Five studies measured maternal anxiety, showing significant statistical heterogeneity. The summary SMD was ‐0.52, which is a medium effect size but of marginal clinical significance. Four studies measured maternal stress, showing significant statistical heterogeneity. The summary SMD was ‐0.71, which is a medium effect size and of clinical significance.

**Conclusion:** Systematic review and meta‐analysis provides evidence for a clinically significant increase in breastmilk quantity when a relaxation intervention is provided. There is evidence for a moderate, clinically significant, reduction in maternal stress and a smaller reduction in maternal anxiety, of marginal clinical significance. Given the low potential harm and high availability of relaxation interventions, they can be recommended, particularly for women with concerns over milk quantity and/or stress.

## Transforming Breastfeeding Culture: Implementation of Breastfeeding‐Friendly Community Initiative (BFCI)

Kris Y. W. Lok^1^, Heidi S. L. Fan^1^, Janet Y. H. Wong^2^, Christine Lam^3^, Marie Tarrant^4^, Patrick Ip^1^, Hextan Ngan^1^, Chia‐Chin Lin^1^

^1^University of Hong Kong, Hong Kong, China. ^2^Hong Kong Metropolitan University, Hong Kong, China. ^3^Queen Elizabeth Hospital, Hong Kong, China. ^4^University of British Columbia, Okanagan, Canada

**Background:** The global WHO/UNICEF Baby Friendly Hospital Initiative (BFHI) is a set of maternity practices that hospitals should implement to support breastfeeding. The BFHI is an evidence‐based effective intervention that contributes to increasing the initiation, exclusivity and duration of breastfeeding. In 2009, WHO encouraged health care systems to scale up the protection, promotion, and support of breastfeeding at the community level. Community support through training and social marketing is a vital link in an effective and sustainable program. The aim is to provide training based on an accredited framework (BFCI) for staff, management and mothers among public places in the community to promulgate breastfeeding friendly attitudes.

**Methods:** This study used a quasi‐experimental pretest‐post test design. From 2020 to 2022, we conducted 70 midwife led breastfeeding workshops online and recruited 2330 pregnant women from social media. Participants completed questionnaires before, immediately after, and 1 month after the workshop. Breastfeeding self‐efficacy and attitude were measured by the Breastfeeding Self‐Efficacy Scale‐Short Form (BSES‐SF) and Iowa Infant Feeding Attitude Scale (IIFAS), respectively. In addition, we recruited 1339 staff and management among public places in the community. Participants completed questionnaires on breastfeeding knowledge and attitudes pre and post workshop.

**Findings:** Among staff there was a significant improvement in both breastfeeding knowledge (*p* < 0.001) and staff attitude towards breastfeeding (*p* < 0.001) after the training workshops. The staff considered that the programme raised awareness of breastfeeding benefits, with 88.4% reported that they feel more confident supporting mothers in breastfeeding after the training. Among mother participants’ who have completed the pre‐ and postworkshop questionnaires, both the breastfeeding self‐efficacy (*p* < 0.001) and breastfeeding attitude (*p* < 0.001) have improved significantly after the training workshops. The improvement in self‐efficacy and breastfeeding attitude appeared to maintain 1 month after the workshop. There were 93.9% of the mother participants’ breastfeeding at the 1‐month post‐training follow‐up, among which half were still exclusively breastfeeding (48.3%).

**Conclusion:** Our findings highlight how the BFCI was provided and the positive experiences implementing BFCI is consistent with other developed countries. The next step is mapping this based on Complex Adaptive system based Breastfeeding Gear Model and provide additional evidence to inform breastfeeding policy based to advance or extend the breastfeeding protection, promotion and support globally in the community.

## The ripple effect of traumatic birth on breastfeeding, caregiving, and bonding: How breastfeeding served as a psychological protective factor for new mothers

Deborah Losada

Fielding Graduate University, Santa Barbara, CA, USA

**Background:** Traumatic birth, defined as causing deep distress that extends beyond the birth experience, can lead to detrimental outcomes for the maternal‐infant relationship. The benefits of breastfeeding have been well‐established and may counteract the sequelae from traumatic birth. The aims of this study were to (a) comprehend what led to traumatic birth and its impact on breastfeeding, caregiving, and bonding; (b) determine if and how breastfeeding may serve as a protective factor after traumatic birth; and (c) improve future care practices for perinatal women based on these findings.

**Methods:** Through purposive sampling, 13 participants with 15 traumatic births recruited from the Southwest and Midwest regions of the United States completed demographic and diary questions via Survey Monkey, followed up by Zoom one‐on‐one semi‐structured interviews. The diary‐interview method and photovoice aided in gathering data that was then analysed through interpretative phenomenological analysis (IPA).

**Findings:** Participants shared narratives that ranged from 3 months to 20 years after their traumatic birth experiences. Nine superordinate themes emerged with traumatic birth creating a ripple effect for certain themes under breastfeeding, caregiving, and bonding. **Traumatic birth themes were:** Setting the stage; A terrifying ordeal; and Lost ideals of motherhood. The ripple effect themes included: Enduring challenges; Lost ideals of motherhood; Going to the extreme to care for you; and Support: It takes a village. Unique themes also appeared for caregiving: The parenting journey: From guilt to grace and for bonding: My motivation to persevere.

**Conclusion:** Study implications indicate there may be a higher prevalence of traumatic birth than previously anticipated, breastfeeding may act a psychological protective factor after traumatic birth for new mothers, and current cultural norms and hospital policies may hinder optimal maternal‐infant bonding and breastfeeding success. Further research is necessitated to better understand the long‐term effects of traumatic birth on breastfeeding, caregiving, and bonding, how breastfeeding can be psychologically protective for mothers and infants, and the types of support that could enhance functioning for postpartum women and their families.

**References**

Greenfield, M., Jomeen, J., & Glover, L. (2016). What is traumatic birth? A concept analysis and literature review. *British Journal of Midwifery*, *24*(4), 254‐267. https://doi.org/10.12968/bjom.2016.24.4.254

Losada, D. S. (2022). *The Journey of Perinatal Women's Lived Experience of Traumatic Birth, Breastfeeding, Caregiving, and Bonding Using Photovoice and the Diary‐Interview Method* (29061656) [Doctoral dissertation, Fielding Graduate University]. ProQuest Dissertations and Theses Global. https://www.proquest.com/docview/2645847014

Smith, J. A., Flowers, P., & Larkin, M. (2009). *Interpretative phenomenological analysis:Theory, method and research*. SAGE.

Victora, C. G., Bahl, R., Barros, A. J., França, G. V., Horton, S., Krasevec, J., Murch, S., Sankar, M. J., Walker, N., & Rollins, N. C. (2016). Breastfeeding in the 21st century: Epidemiology, mechanisms, and lifelong effect. *The Lancet, 387*(10017), 475‐490. https://doi.org/10.1016/S0140-6736

## First breastfeeding attempt in preterm infants: Early timing and performance with and without nasal‐CPAP. A multicentre cohort study

Ragnhild Maastrup^1^, Ane Rom^2^, Sisse Walloee^3^, Helle B. Sandfeld^4^, Hanne Kronborg^5^

^1^Department of Neonatology, Copenhagen University Hospital Rigshospitalet, Copenhagen University Hospital, Copenhagen, Denmark. ^2^Research Unit Women's and Children's Health, Juliane Marie Centre, Copehangen University Hospital Rigshospitalet, Copenhagen, Denmark. ^3^OPEN ‐ Patient data Explorative Network, Dept of Clinical Research, University of Southern Denmark, Odense, Denmark. ^4^Department of Paediatrics, Randers Hospital, Randers, Denmark. ^5^Department of Public Health, Section for Nursing, Aarhus University, Aarhus, Denmark

**Background and aim:** The Baby‐friendly Hospital Initiative for neonatal wards (Neo‐BFHI) and The World Health Organization (WHO) recommend that stable preterm infants should initiate breastfeeding regardless of gestational age (GA), postmenstrual age (PMA), birthweight or current weight (Nyqvist et al., 2013, WHO/UNICEF 2020). The aim of the study was to describe PMA and performance at the first breastfeeding attempt in preterm infants using Neo‐BFHI's and WHO's recommendations and to detect differences across GA groups with and without nasal‐CPAP.

**Methods:** A multicentre cohort study from 13 Danish NICUs based on questionnaires answered by mothers of 992 preterm infants with GA 23–36 weeks.

**Results:** The lowest PMA at first breastfeeding attempt was 27,6 weeks. Of the extremely and very preterm infants, 61% and 46%, respectively, had the first breastfeeding attempt before PMA 32 weeks. During first breastfeeding attempt 29% were treated with nasal‐CPAP. There was a positive correlation between low GA and low PMA at first breastfeeding attempt (Pearson's correlation 0.765, *p* < 0.0001). Performance at the preterm infants first breastfeeding attempt were as follows: 24% “Smell the breast”, 23% “Lick and taste the milk”, 32% “Seek and find breast, get nipple in mouth”, 19% “Suckle and swallow briefly”, and very few infants displayed a breastfeeding behaviour beyond this. Performance was in general not affected by nasal‐CPAP treatment.

**Conclusion:** Stable preterm infants had their first breastfeeding attempt at a very early stage, even during nasal‐CPAP treatment. At the first breastfeeding attempt 78% of preterm infants did not swallow. Preterm infants need time to familiarize with the breast and hence all preterm infants should be allowed to follow their own pace in breastfeeding.

**References**

Nyqvist, K.H., Häggkvist, A.P., Hansen, M.N., Kylberg, E., Frandsen, A.L., Maastrup, R., … Haiek, L.N. (2013). Expansion of the Baby‐Friendly Hospital Initiative Ten Steps to Successful Breastfeeding into Neonatal Intensive Care: Expert Group Recommendations. *Journal of Human Lactation*, 29(3), 300‐9.

World Health Organization/UNICEF. (2020) *Protecting, promoting and supporting breastfeeding: the Baby‐friendly Hospital Initiative for small, sick and preterm newborns*. Geneva: World Health Organization and the United Nations Children's Fund (UNICEF). Retrieved from https://www.who.int/publications/i/item/9789240005648

## Analyzing the relationship between breastfeeding support and the incidence of childhood obesity using spatial analysis

Ellie MacGregor^1^, Anna Blair^2^, Nikki Lee^3^

^1^Academy of Lactation Policy and Practice, MA, USA. ^2^Healthy Children Project, Harwich, MA, USA. ^3^Private Practice, Tennessee, USA

**Background:** As the prevalence of childhood obesity continues to rise, identification of potential interventions by public health policy makers and health care providers is imperative. Breastfeeding, the most optimal method of infant feeding, has been demonstrated to protect against childhood obesity. Spatial analysis can be employed to examine the relationship between childhood obesity, breastfeeding rates and lactation care providers in a geographic region to target resources and interventions.

**Method:** A cross‐sectional, observational spatial analysis using retrospective data from the CDC, the State of Pennsylvania (USA) and professional lactation credentialing organizations.

**Findings:** County‐level breastfeeding rates, childhood obesity rates, and the number and type of lactation care provider were tested for significance at the *p* < 0.01 level using a two‐tailed significance test and bivariate Pearson's correlation. The results show a significant, inverse relationship between breastfeeding rates and childhood obesity prevalence at the county level, *p* < 0.01. There is also a significant, inverse relationship between the number of CLCs and the number of all professional lactation support providers and childhood obesity rates at the county level, *p* < 0.01. Thus, the availability of breastfeeding support is significantly related to breastfeeding rates and inversely related to childhood obesity rates across Pennsylvania.

**Conclusion:** Lactation support providers play a key role in providing education, care, and support to families considering a feeding choice. Access to professional lactation care increases breastfeeding initiation, exclusivity, and duration rates. In addition, the availability of breastfeeding support is significantly inversely related to childhood obesity rates across Pennsylvania.

**References**

Blair, A., MacGregor, E., & Lee, N. (2020). Childhood Obesity and Breastfeeding Rates in Pennsylvania Counties—Spatial Analysis of the Lactation Support Landscape. Frontiers in Public Health, 8, 123.

Centers for Disease Control & Prevention, Division of Nutrition, Physical Activity, and Obesity, National Center for Chronic Disease Prevention and Health Promotion. State Obesity Prevention Efforts Targeting the Early Care and Education Setting. (2018) Retrieved from: https://www.cdc.gov/obesity/ strategies/early‐care‐education/pdf/ECE_2018_QuickStartActionGuide_April2018_508. pdf (accessed on January 29, 2020).

Wang L, Collins C, Ratliff M, Zie B, Wang Y. Breastfeeding reduces childhood obesity risks. Child Obes. (2017) 13:197–204. https://doi.org/10.1089/chi.2016.0210

World Health Organization. Exclusive Breastfeeding to Reduce the Risk of Childhood Overweight and Obesity. (2014). Retrieved from: https://www.who.int/elena/titles/bbc/breastfeeding_childhood_obesity/en/ (accessed January 31, 2020).

## ‘Follow My Cue, Aye?’ Neonatal Cue Based Responsive Feeding QI project

L. McKerracher, C. Leong, K. Nicallen‐Roeder M. White, E. Gray, J. Nokes, J. Duncan, S. Chouman, L. Shaw, A. Wright

Neonatal Unit Ninewells Hospital, Dundee, UK

**Background:** There is growing appreciation of the immediate and longer term physical and developmental benefits of responsive feeding for babies as well as the positive effects on the infant‐caregiver relationship.^1^ In preterm babies within the neonatal unit setting, the transition from nasogastric tube feeding to orally feeding by breast or by bottle has traditionally been facilitated by the giving of a prescribed volume of feed given at strict, regular intervals; commencement of trial of suck feeds then happens according to gestation, weight or staff perception of readiness.^2^ In contrast, in a cue based responsive feeding approach, parents and staff are empowered to sensitively offer babies oral feeding experiences based on the display of developmentally appropriate feeding cues. Cue based feeding within the NICU has been associated with shorter time to acquisition to full oral feeding; shorter hospital stay and fewer adverse events.^3^

**Methods:** In August 2022, the Ninewells Hospital NICU began a cue based feeding quality improvement project. With involvement from neonatal nursing and medical staff, the neonatal AHP team, psychology and with parental engagement, a cue based responsive feeding approach was incorporated into routine care for all fully enterally fed babies >32 weeks.

**Results:** The NHS Scotland *‘My Feeding Journey ‐ Am I ready to Feed?*’ guide was modified and babies are now offered to breastfeed or bottle feed based on their cue based feeding score of 1‐5, as determined by staff and parents in partnership. The initial aim of the project was to achieve documentation of cue based feeding scores in 60% of feeds for all enterally fed babies >32 weeks by December 2022.

**Conclusions:** To date there has been a hugely positive response from parents and staff alike with a tangible culture shift in the approach to responsive feeding within the neonatal unit. Our data show there have been week on week improvements in the primary outcome measure of cuebased feeding score documentation which supports that active assessment and recognition of babies’ feeding cues is being incorporated into daily care. The project continues; monitoring of average weekly weight gain and qualitative data collection around parental & staff feedback is ongoing.

**References**

1. Ventura, A. K. (2017). Associations between Breastfeeding and Maternal Responsiveness: A Systematic Review of the Literature. *Advances in Nutrition: An International Review Journal*, *8*(3), 495–510. https://doi.org/10.3945/an.116.014753

2. White, A., & Parnell, K. (2013). The transition from tube to full oral feeding (breast or bottle) – A cue‐based developmental approach. *Journal of Neonatal Nursing*, *19*(4), 189–197. https://doi.org/10.1016/j.jnn.2013.03.006

3. Lubbe, W. (2017). Clinicians guide for cue‐based transition to oral feeding in preterm infants: An easy‐to‐use clinical guide. *Journal of Evaluation in Clinical Practice*, *24*(1), 80–88. https://doi.org/10.1111/jep.12721

## Allies for racial equity in breastfeeding: From the outside looking in

Anne Merewood and Farzana Hakimi

Boston University School of Medicine, Boston, USA

**Background:** The Center for Health Equity, Education, and Research (CHEER) in Boston, US, and CHEER International Group have worked with disadvantaged populations for 25 years. The work includes working with American Indian/Alaska Native populations; the African American community in Mississippi, with refugees in Greece, and most recently, creation of a breastfeeding support program in Herat, Afghanistan.

**Methods:** Using data driven outcomes, and a range of community based initiative engagement strategies, we present a long term perspective on an institutional and personal experience of engaging with underserved groups, and on fighting institutional racism as allies.

**Results:** We will share successful outcomes, for example, 100% Baby‐Friendly hospital designation with the Indian Health Service, increases in population breastfeeding rates in Mississippi, and establishment of breastfeeding programs in extreme circumstances including refugee camps in Greece, and in Herat, Afghanistan. We believe success in large part is due to establishment of trusting relationships with community partners and representative groups. Participants will be able to gain insights into successful programing and community engagement strategies as well as to working in complex circumstances amid institutionally racist settings.

**Conclusion:** Academic and community based programs locally, nationally, and internationally can work together to create successful strategic approaches to support breastfeeding in a range of settings and with an emphasis on racial

## Using an implementation science framework to assess a breastfeeding initiative: RE‐AIM and Mississippi CHAMPS

Anne Merewood^1^, Laura Burnham^2^, Jacqueline Berger^3^, Aishat Gambari^4^

^1^Boston University School of Medicine, Boston, USA. ^2^Boston Medical Center, Boston, USA. ^3^Georgia Southern University, Statesboro, USA. ^4^Emory University, Atlanta, USA

**Background:** We used RE‐AIM, a well‐established implementation science framework measuring Reach, Effectiveness, Adoption, Implementation, and Maintenance, to evaluate the broader impact of a statewide breastfeeding initiative including 38 hospitals, that has been ongoing since 2014. Mississippi had one of the lowest breastfeeding rates in the US.

**Methods:** Mississippi CHAMPS (Communities and Hospitals Advancing Maternity Practices) supported hospitals to adopt the Ten Steps to Successful Breastfeeding, to gain Baby‐Friendly designation, and to engage intensively with local community partners and organizations to ensure that mother's voices were equitably represented in conversations around breastfeeding support in the hospital and community. We applied the RE‐AIM analysis methods, using quantitative and qualitative data to assess the Reach, Effectiveness, Adoption, Implementation, and Maintenance of the program components.

**Results:** The RE‐AIM framework proved a successful vehicle for assessing a large‐scale breastfeeding initiative. We found that MS CHAMPS reached 98% of eligible birthing women (99% of African American women) and 65% of WIC breastfeeding peer counselors throughout the state. Effectiveness: Average hospital breastfeeding initiation rates increased significantly from 56% to 66% and racial disparities decreased by 17%. Adoption: 95% of eligible Mississippi hospitals enrolled into CHAMPS (39/41), and the program engaged with 25 community‐based organizations, in various capacities. Implementation: 20 CHAMPS hospitals gained Baby‐Friendly status (up from 0) and the proportion of hospitals designated Baby‐Friendly or in the final stages of the pathway rose from 15% to 90%. Maintenance: CHAMPS maintains a funded presence in Mississippi, and all designated hospitals maintained Baby‐Friendly status.

**Conclusion:** An established implementation science framework was a useful tool by which to assess a breastfeeding program, and such frameworks could be used in the future to evaluate public health approaches to breastfeeding. Widespread change in healthcare practices is possible when implemented in partnership with diverse institutions and community groups.

## Building the Evidence for Skilled Lactation Support Delivery through the Canada Prenatal Nutrition Program: Overview of a Multi‐Phased Implementation Research Programme

Alison Mildon^1^, Jane Francis^2^, Bronwyn Underhill^3^, Stacia Stewart^3^, Yi Man Ng^3^, Christina Rousseau^4^, Jo‐Anna Baxter^1^, Deborah O'Connor^1,5^, Daniel Sellen^1^

^1^University of Toronto, Toronto, Ontario, Canada. ^2^Acadia University, Wolfville, Nova Scotia, Canada. ^3^Parkdale Queen West Community Health Centre, Toronto, Ontario, Canada. ^4^The Stop Community Food Centre, Toronto, Ontario, Canada. ^5^The Hospital for Sick Children, Toronto, Ontario, Canada

**Alison Mildon and Jane Francis are co‐first authors**.

**Deborah O'Connor and Daniel Sellen are co‐senior authors**

**Background:** Skilled lactation support is less accessible to socially/economically disadvantaged families in high‐income countries, contributing to breastfeeding inequities (Cattaneo, 2012; WHO, 2018). Integrating lactation support within community perinatal services can help address this problem (Rollins et al., 2016). The national Canada Prenatal Nutrition Program (CPNP) targets vulnerable families through a flexible community‐based programming model. Core activities include health and nutrition education, food supports, and referrals to other services. The CPNP aims to support breastfeeding but lacks a framework for the delivery of lactation support. Our academic‐community partnership is addressing this gap through an active multi‐phased implementation research programme.

**Methods:** Research priorities and designs of this partnership are determined collaboratively, with lead researchers embedded within participating CPNP sites. Phase one included prospective cohort and qualitative studies at one CPNP site in Toronto where a charitably‐funded lactation support program offers free in‐home lactation consultant services and breast pumps as an enhancement to core CPNP services. Phase two aimed to test the effectiveness and feasibility of delivering similar lactation supports in two additional Toronto CPNP sites. Phase two was suspended during COVID‐19 and is currently being redesigned within the current, third phase of research.

**Findings and Implications:** Participating CPNP sites in Toronto primarily served low‐income families and newcomers to Canada. Findings showed high uptake of lactation services and identified free in‐home support delivered by skilled, non‐judgmental providers as key intervention elements (Francis et al., 2020). Breastfeeding to 6 months was prevalent (80%) but exclusive breastfeeding was not (16%) (Mildon et al., 2022). Additional results suggesting a need to strengthen prenatal preparation for lactation, mitigate hospital formula supplementation and ensure access to skilled lactation support from birth have informed changes to current lactation services. The next phase of research will evaluate this enhanced delivery model, and a national survey of CPNP sites will identify promising lactation support interventions appropriate for implementation studies in diverse settings, including rural and Indigenous communities. The ultimate aim of this work is to build the evidence for a suite of approaches for delivering lactation support through the CPNP, strengthening programming guidance and contributing to a reduction in breastfeeding inequities.

**Reference**

Cattaneo, A. (2012). Academy of Breastfeeding Medicine founder's lecture 2011: inequalities and inequities in breastfeeding: an international perspective. *Breastfeed Med, 7*, 3‐9. https://doi.org/10.1089/bfm.2012.9999

Francis, J., Mildon, A., Stewart, S., Underhill, B., Tarasuk, V., Di Ruggiero, E., … O'Connor, D. L. (2020). Vulnerable mothers’ experiences breastfeeding with an enhanced community lactation support program. *Matern Child Nutr, 16*, e12957. https://doi.org/10.1111/mcn.12957

Mildon, A., Francis, J., Stewart, S., Underhill, B., Ng, Y. M., Rousseau, C., … Sellen, D. W. (2022). Associations between use of expressed human milk at 2 weeks postpartum and human milk feeding practices to 6 months: a prospective cohort study with vulnerable women in Toronto, Canada. *BMJ Open, 12*, e055830. https://doi.org/10.1136/bmjopen-2021-055830

Rollins, N. C., Bhandari, N., Hajeebhoy, N., Horton, S., Lutter, C. K., Martines, J. C., … Victora, C. G. (2016). Why invest, and what it will take to improve breastfeeding practices? *The Lancet, 387*, 491‐504. https://doi.org/10.1016/s0140-6736(15)01044-2

WHO. (2018). ***Guideline:** counselling of women to improve breastfeeding practices*. Retrieved from https://apps.who.int/iris/bitstream/handle/10665/280133/9789241550468-eng.pdf?ua=1

## Neuropsychological Development Across Birthing and Feeding Modalities

Nathan Mittelstadt^1^, Darby Dickton^2^, Jimi Francis^3^

^1^Texas A&M ‐ San Antonio, San Antonio, USA. ^2^Foundation for Maternal Infant Lactation Knowledge, San Antonio, USA. ^3^University of Texas ‐ San Antonio, San Antonio, USA

**Background:** Long term developmental studies examining birth interventions and feeding modalities have been fraught with difficulties. While neuropsychological evaluation has strongly suggested a causal relationship between breastfeeding and improved cognition in children (Horta et al., 2015), there is some controversy as to the exact developmental abilities that are bolstered with breastfeeding versus bottle feeding and those that are hindered by formula feeding (Der et al., 2006; Park et al., 2014). Even more interestingly, cesarean sections appear to have gender based long term developmental consequences that could not have been predicted by the current common understanding of the birth process (Takács et al., 2021). This pilot study will lay the foundation to build a larger longitudinal study looking at infant and child neuropsychological development in light of birthing interventions that were both required and elective as well as breastfeeding versus formula practices, including exclusive breastfeeding or bottle feeding, mixed feeding modalities and formula use.

**Methods:** Mothers recruited will be between the ages of 18‐35, with children between 1 month old and 5 years old. Birthing and feeding data will be self‐reported by the mother, while the child will be assessed via maternal survey using the Denver II, Ages and Stages Questionnaire, Brazelton Neonatal Behavioral Assessment Scale, and the Psychomotor Development Index.

**Results:** Preliminary data reveals differences in outcome measures based on mode of delivery and mode of feeding.

**Conclusion:** Due to the advancement of modern medicine, delivery and feeding interventions are ever‐expanding with poorly understood long‐term consequences. By assessing birthing interventions and feeding modalities in collaboration with thorough neuropsychological evaluation, we can identify further long‐term benefits and drawbacks of contemporary methods. This improved understanding of developmental impact will further clarify birthing and feeding standard of care to optimize the outcome for every child.

**References**

Der, G., Batty, G. D., & Deary, I. J. (2006). Effect of breast feeding on intelligence in children: prospective study, sibling pairs analysis, and meta‐analysis. BMJ, 333(7575), 945. https://doi.org/10.1136/bmj.38978.699583.55

Horta, B. L., Loret De Mola, C., & Victora, C. G. (2015). Breastfeeding and intelligence: A systematic review and meta‐analysis. Acta Paediatrica, International Journal of Paediatrics, 104, 14–19. https://doi.org/10.1111/APA.13139

Park, S., Kim, B. N., Kim, J. W., Shin, M. S., Yoo, H. J., & Cho, S. C. (2014). Protective effect of breastfeeding with regard to children's behavioral and cognitive problems. Nutrition Journal, 13(1), 1–5. https://doi.org/10.1186/1475-2891-13-111/TABLES/2

Takács, L., Putnam, S. P., Monk, C., Dahlen, H. G., Thornton, C., Bartoš, F., Topalidou, A., & Peters, L. L. (2021). Associations Between Mode of Birth and Neuropsychological Development in Children Aged 4 Years: Results from a Birth Cohort Study. Child Psychiatry and Human Development, 52(6), 1094–1105. https://doi.org/10.1007/S10578-020-01084-4/TABLES/4

## Single‐family room in neonatal intensive care: A qualitative analysis of fathers', mothers' and nurses' experiences

Joan Neergaard Larsen^1^, Helena Hansson^2^, Sanne Allermann Beck^1^, Vibeke Zoffmann^3^

^1^Department of Neonatal and Peadiatric Intensive Care, Copenhagen University Hospital, Copenhagen, Denmark. ^2^Department of Paediatric and Adolescent Medicine, Copenhagen University Hospital, copenhagen, Denmark. ^3^The Interdisciplinary Research Unit of Women's, Children's and Families’ Health, Copenhagen University Hospital, copenhagen, Denmark

**Background:** A sub‐division of a Neonatal Intensive Care Unit (NICU) was because of water damage forced to relocate to temporary premises with single‐family rooms (SFRs). This new setting offered a unique opportunity to explore challenges and advantages of using SFRs moving to a new‐constructed Children's Hospital in 2026 with SFR. Supporting father‐baby bonding and co‐parenting has increased and shows improvement in newborn's health, however engaging fathers remains suboptimal (1). This study investigates the real‐time experiences of parents and nurses with the SFR.

**Methods:** Two focus group interviews were conducted in June 2021. Fathers and mothers of newborns occupying SFRs participated in one interview (n = 6, 22 invited) as did Department nurses (n = 5, 11 invited). Participants were provided with a set of unfinished sentences to complete in writing as self‐reflection before the interview, and a semi‐structured interview guide from a prior study in 2006 was used for the process (2). Interviews underwent thematic analysis.

**Results:** Five themes were identified: 1) A place for joy and sorrow; 2) Room for free expression and the father's advanced potential; 3) Simulating home; 4) Self‐determined connection to the world from a safe place, and 5) Outer silence contrast inner turmoil in nurses. The SFR allowed for parental self‐expression in private, initiating family bonding, and self‐determining when and how to connect with the world. The fathers showed great potential in taking an active role and it seemed that SFR created a suitable frame. Though nurses faced challenges with a lack of departmental overview they found SFR valuable to the families.

**Conclusion:** The study shows that SFRs are beneficial to families while nurses may face communication challenges and an increased workload.

**References**

1. Fisher D, Khashu M, Adama EA, Feeley N, Garfield CF, Ireland J, et al., Fathers in neonatal units: Improving infant health by supporting the baby‐father bond and mother‐father coparenting. Journal of neonatal nursing: JNN. 2018;24(6):306‐12.

2. Beck S, Weis J, Greisen G, Andersen M & Zoffmann V Room for family‐centered care ‐ a qualitative evaluation of a neonatal intensive care unit remodeling project. Journal of Neonatal Nursing, bind 15, nr 3, s 88‐99. 2009

## Systematic review of reviews: Risks, protective factors and potential mechanisms of psychosocial well‐being of parents of preterm and/or sick infants

Rebecca Nowland, Raessa Jassat, Gill Thomson

University of Central Lancashire, Preston, United Kingdom

**Background:** Parents of infants who require neonatal care face numerous challenges (Flacking et al., 2012) which can be associated with increased risks of anxiety, depression, and stress/trauma and when compared with parents of term infants (Karatzias et al., 2007; Vigod et al., 2010). There are systematic reviews on the experiences and needs of parents with preterm infants and associated health and well‐being outcomes, but review findings have not been comprehensively synthesised.

**Methods:** A systematic review of reviews examining experiences/needs of parents with preterm and/or sick infants during the hospital stay and following discharge was conducted to identify risk and protective factors for psychosocial well‐being and potential mechanisms that may help to reduce poor psychosocial well‐being. Database searches were conducted on eight bibliographic databases: PsychInfo, CINAHL, Cochrane, MEDLINE (Ovid), Web of Science, Embase and Global Index Medicus. Handsearching involved google searches, reference checking, citation chaining and examination of author's personal files. A date restriction of 2000 to present day was applied and only reviews written in English were included. Research quality and bias was assessed using the AMSTAR 2 critical appraisal tool for systematic reviews (Shea et al., 2017). Thematic analysis (Braun & Clarke, 2012) of review findings was conducted.

**Findings:** In all 3000 articles were obtained, 6 of which were identified through handsearching, with 27 articles meeting the inclusion and exclusion criteria. Selected reviews were scoping (n = 4), qualitative (n = 6), quantitative (n = 4) or integrative (n = 12) and a meta‐analysis (n = 1). Data relating to risks and protective factors and mechanisms were categorised and charted using the socioecological model. Thematic analysis revealed 5 themes relating to supporting parents’ psychosocial needs: parents being valued and included in caregiving, developing trusting relationships with staff, screening for psychosocial factors, supporting and enabling hope, spirituality and optimism and the importance of peer support.

**Conclusion:** Findings highlight the importance development of staff‐parent relationships, opportunities for peer support and building coping and resilience in parents as potential interventions in neonatal units to promote parents’ psychosocial well‐being.

**Reference**

Braun, V., & Clarke, V. (2012). Thematic analysis. American Psychological Association.

Flacking, R., Lehtonen, L., Thomson, G., Axelin, A., Ahlqvist, S., Moran, V. H., … & SCENE group. (2012). Closeness and separation in neonatal intensive care. Acta Paediatrica, 101(10), 1032‐1037.

Karatzias, T., Chouliara, Z., Maxton, F., Freer, Y., & Power, K. (2007). Posttraumatic symptomatology in parents with premature infants: a systematic review of the literature. Journal of Prenatal and Perinatal Psychology and Health, 21(3), 249.

Shea, B. J., Reeves, B. C., Wells, G., Thuku, M., Hamel, C., Moran, J., … & Henry, D. A. (2017). AMSTAR 2: a critical appraisal tool for systematic reviews that include randomised or non‐randomised studies of healthcare interventions, or both. BMJ, 358.

Vigod, S. N., Villegas, L., Dennis, C. L., & Ross, L. E. (2010). Prevalence and risk factors for postpartum depression among women with preterm and low‐birth‐weight infants: a systematic review. BJOG: An International Journal of Obstetrics & Gynaecology, 117(5), 540‐550.

## Designing milk banking services of the future: Using implementation science to integrate and scale interventions that support lactation, and protect infant and maternal health

Natalie Shenker^1,2^, Gillian Weaver^2^

^1^Imperial College London, London, United Kingdom. ^2^Human Milk Foundation, Hertfordshire, United Kingdom

**Background:** Human milk banks (HMBs) recruit and screen human milk donors, and collect, process, screen, store and distribute donor human milk (DHM). DHM was widely available in the UK in the 1970s but most HMBs closed during the 1980s after costs and barriers to donation rose, and specialist preterm infant formulas were introduced. As DHM became limited, its usage became rationed to only the most preterm infants for whom human milk diets reduce the risk of life‐threatening complications. In response to HMB service fragility and a growing inequity of access, the Hearts Milk Bank was founded in 2017 to offer assured DHM provision and facilitate research. Hearts now provides DHM to over 50 NHS hospital neonatal units in England and Wales. Alongside, the Hearts team has pioneered the use of DHM to support mothers attempting to establish lactation, or for parents unable to lactate, in a community programme for families that are ineligible for access to DHM on the National Health Service (Griffin et al., 2022). Evaluation of this service has shown positive impacts on infant growth (Bramer et al., 2021), maternal breastfeeding outcomes (Bramer et al., 2021), parental wellbeing (Brown and Shenker, 2022), and maternal anxiety and depression (Brown et al., in preparation, 2022). This presentation will describe the programme of implementation science now underway to understand key elements of scalability of HMB services in the UK.

**Methods/results:** By assimilating data from investigations into acceptability, costings, cost‐effectiveness, operational and geographical modelling, technological innovation, optimisation of donor recruitment and support, service continuity planning and plans for randomised controlled trials, this presentation will present a future pathway for HMB service integration into existing hospital and community‐based lactation support services.

**Conclusion:** If gains in perinatal and infant health can be extrapolated, HMBs could represent a key missing piece in the jigsaw puzzle of available lactation support services in the UK.

**References**

Bramer S, Boyle R, Weaver G, Shenker N. Use of donor human milk in nonhospitalized infants: An infant growth study. Matern Child Nutr. 2021 Apr;17(2):e13128. https://doi.org/10.1111/mcn.13128. Epub 2021 Jan 6. PMID: 33404169; PMCID: PMC7988867.

Brown A, Shenker N. Receiving screened donor human milk for their infant supports parental wellbeing: a mixed‐methods study. BMC Pregnancy Childbirth. 2022 May 31;22(1):455. https://doi.org/10.1186/s12884-022-04789-7. PMID: 35641919; PMCID: PMC9154035.

Griffin S, Watt J, Wedekind S, Bramer S, Hazemi‐Jebelli Y, Boyle R, Weaver G, Shenker NS. Establishing a novel community‐focussed lactation support service: a descriptive case series. Int Breastfeed J. 2022 Jan 15;17(1):7. https://doi.org/10.1186/s13006-021-00446-5. PMID: 35033128; PMCID: PMC8760776.

## Towards Coproduction of a Public Health Breastfeeding Campaign

Jill Smith^1^, Jennifer Nicol^1^, Kate Parsons^2^, Amy McDonald^1^

^1^Small Steps Big Changes, Nottingham, United Kingdom. ^2^Aberystwyth University, Aberysthyth, United Kingdom

**Background:** Improving breastfeeding initiation and continuation rates is a United Kingdom public health priority. Small Steps Big Changes (SSBC, is one of five 5 UK based sites, funded for 10 years by the National Lottery Community as part of their A Better Start Programme) champions coproduction with parents and workforce to test science‐based interventions aimed at improving early child development outcomes, including nutrition. Across SSBC wards rates of any breastmilk initiation range from 42% to 60%, with 6‐8 weeks data 31% to 62% (SystemOne, 2022).

**Methods:** SSBC commissioned a social marketing firm to develop a breastfeeding public health campaign, underpinned by self‐determination theory (Deci & Ryan, 2017). The campaign seeks to address not just the breastfeeding dyad but what has been shown as important; the communities that exist around them (Brown 2016). A mixed methods social marketing survey, completed by 1800 mostly female, White British respondents, many with breastfeeding experience, explored community perceptions and social norms alongside drivers and barriers, in relation to breastfeeding. Reactions to previous breastfeeding campaigns were captured. Additionally, males’ views and women who lacked breastfeeding confidence were explored via two focus groups. Free text survey responses were analysed deductively using a self‐determination theory framework.

**Results:** Analysis showed self‐determination theory constructs at play in local breastfeeding decision‐making. Lack of autonomy, relatedness and competence provided reasons for low initiation and continuation of breastfeeding. Women who breastfed for longer often perceived their behaviour was self‐directed, they had self‐efficacy in tackling breastfeeding challenges and were supported to breastfeed through family support and perceived social norms. These key findings were used to develop campaign concepts and messaging. Design elements, tone and segmentation of messaging for different elements of the breastfeeding ecology were tested with 139 people, a subset of the original respondents.

**Conclusions:** The resulting co‐produced campaign draws on behaviour change theory and local research alongside parent and workforce knowledge. Parent voice was present throughout and further consultation with maternity, community and public health stakeholders informed development. The messaging prioritises autonomy, self‐efficacy and relatedness over health and benefit‐based content. Local case studies reflect these themes and provide a sense of place, with case studies pictured feeding in identifiable locations. The campaign assets and website explore the challenges of breastfeeding. The campaign will be evaluated.

**References**

Brown, A. (2016). What do women really want? Lessons for breastfeeding promotion and education. *Breastfeeding medicine*, *11*(3), 102‐110.

Ryan, R. M., & Deci, E. L. (2017). Self‐determination theory: Basic psychological needs in motivation, development, and wellness. New York, NY: Guilford Publishing.

SystmOne Data (2022). [Nottingham CityCare Children's Public Health 0‐19 Nursing Service data set] [Unpublished raw data] Nottingham CityCare.

## Exploring how midwives can support the early mother‐infant relationship: A mixed‐method study

Cathy Stoodley^1^ (BMid, MMid), Lois McKellar^2^ (RM, PhD), Tahereh Ziaian^1^ (PhD), Jennifer Fereday^1^ (RM, PhD), Mary Steen^3^ (RM, PhD) and Ian Gwilt^1^ (PhD)

^1^University of South Australia, Australia. ^2^School of Health and Social Care, Edinburgh Napier University. ^3^University of Northumbria, Newcastle

**Background:** The mother‐infant relationship is complex and dynamic, influencing the psychological development of the infant through bonding and attachment (Bicking Kinsey & Hupcey, 2013). Positive early interactions influence the quality of this relationship (Hairston, Handelzalts, Lehman‐Inbar, & Kovo, 2019). Midwives are well placed to support the developing relationship between the mother and baby, yet there has been limited research exploring the role of the midwife in this context.

**Aims:** The aim of this study was to explore how midwives can support the emerging mother‐infant relationship in the early postnatal period in the context of cultural diversity and to develop a supportive intervention.

**Methods:** A mixed‐method, exploratory study was undertaken. Stage one included a scoping review and Codesign workshops with mothers, midwives, and other stakeholders. To inform the Codesign process 14 women engaged in an activity with prompts to encourage reflection on their experiences and influences of the early mother‐infant relationship. This activity was followed by online interviews.

**Findings:** Key themes from the transcribed interviews included; making moments that matter; the role of the village; feeling like I'm winning; integrating the family and the role of health professionals. Following this, three codesign workshops explored the themes further and a variety of strategies to support the mother‐infant relationship were proposed.

**Discussion:** It was evident from the findings that strategies to support the early mother infant relationship included supporting mothers directly, as well as encouraging midwives to embed appropriate support within their practice. A health promotion activity was developed for midwives to provide education and ways to support the mother‐baby relationship.

**Conclusion:** Codesign provided a valuable approach to bring together knowledge and expertise across a range of stakeholders. It was evident that there is a need to raise awareness of how midwives can play a positive role in supporting the early mother‐infant relationship.

## Breastfeeding in very preterm twins: A prospective longitudinal comparative study

Bente Silnes Tandberg^1,2^, Hege Grundt^3^, Atle Moen^4^, Hannakaisa Niela‐Vilén^5^, Renée Flacking^6^

^1^Vestre viken HT, Drammen, Norway. ^2^Lovisenberg Diaconal University College, Oslo, Norway. ^3^Department of Neonatology, Haukeland University Hospital, Bergen, Norway. ^4^Department of Neonatology, Oslo University Hospital, Oslo, Norway. ^5^Department of Nursing Science, University of Turku, Turku, Finland. ^6^School of Health and Welfare, Dalarna University, Falun, Sweden

A few studies have reported a shorter breastfeeding duration in preterm twins compared to preterm singletons (Dong et al., 2022; Maastrup et al., 2014; Quitadamo et al., 2021). The aim of this study was to compare skin‐to‐skin contact, breastfeeding and mothers breastfeeding self‐efficacy of very preterm (28–32 gestational weeks) twins and singleton infants.

**Methods:** A longitudinal cohort design, including 49 singleton infants and 28 twins and their mothers. Data were prospectively collected on parental skin‐to‐skin contact, breast milk volume, breast milk feeding, direct breastfeeding and breastfeeding self‐efficacy, from birth to 4 months of corrected age.

**Results:** Twin mothers performed 157 min of skin‐to‐skin contact per day/per twin infant, compared to 244 min per singleton infant (< 0.001). The first breastfeeding attempt occurred for twins at a median of 133 h after birth compared to 56 h for singletons (0.002). There were no differences between twins and singletons in receiving their mother's milk or in exclusively direct breastfeeding at any time point. No differences in mothers’ breastfeeding self‐efficacy were found.

**Conclusions:** This study indicates that positive results in breastfeeding duration and exclusivity by direct breastfeeding can be achieved in mothers of very preterm twins in line with preterm singleton infants. This positive finding may contribute to the information and guidance about breastfeeding given to mothers and fathers of preterm twins.

**References**

Dong, D., Ru, X., Huang, X., Sang, T., Li, S., Wang, Y., & Feng, Q. (2022). A prospective cohort study on lactation status and breastfeeding challenges in mothers giving birth to preterm infants. International breastfeeding journal, 17(1), 1‐13.

Maastrup, R., Hansen, B. M., Kronborg, H., Bojesen, S. N., Hallum, K., Frandsen, A., … Hallstrom, I. (2014). Factors associated with exclusive breastfeeding of preterm infants. Results from a prospective national cohort study. PLoS One, 9(2), e89077. https://doi.org/10.1371/journal.pone.0089077

Quitadamo, P. A., Comegna, L., Palumbo, G., Copetti, M., Lurdo, P., Zambianco, F., … Villani, A. (2021). Feeding Twins with Human Milk and Factors Associated with Its Duration: A Qualitative and Quantitative Study in Southern Italy. Nutrients, 13(9), 3099.

## The PosiFeed Neo group benchmark of feeding strategies in Neonatal Care: Development of a Survey on feeding in neonatal unites

Bente Silnes Tandberg^1,2^, Hege Grundt^3^, Renée Flacking^4^

^1^Department of Paediatric and Adolescent Medicine, Drammen Hospital, Vestre Viken Hospital Trust, Drammen, Norway. ^2^Lovisenberg Diaconal University College, Oslo, Norway. ^3^Department of Neonatology, Haukeland University Hospital, Bergen, Norway. ^4^School of Health and Welfare, Dalarna University, Falun, Sweden

In 2021, the network Positive infant feeding in neonatal units (PosiFeed Neo group), was established as part of the larger network Separation and closeness in the neonatal environment (SCENE). PosiFeed Neo is a multidisciplinary network of clinicians, researchers, and parents, with the aim to share knowledge and experiences and to conduct research together. Although feeding support is in focus in neonatal units, there is a lack of evidence‐based guidance. The aims with the PosiFeed Neo research are to evaluate strategies/interventions to secure infants sufficient nutrition and growth where infants’ needs and capacities are acknowledged and contributes to positive feeding experiences in infants and parents.

**Method:** As a first step, current clinical practices and strategies on supportive feeding will be benchmarked across Europe. At present, there are models, strategies, and measures, that have been described to promote feeding behavior in preterm infants (Lubbe, 2018; McFadden et al., 2021; Watson & McGuire, 2016) and how to support parents in the process (Flacking et al., 2021), but the usage and practices in neonatal units are unexplored. Through discussions and Delphi rounds, a questionnaire for neonatal units was developed. The questionnaire examines cue‐based feeding practices and factors (e.g., skin‐to skin contact, early pumping) known to facilitate breastfeeding preterm infants (Maastrup et al., 2014), but also barriers for breastfeeding. The questionnaire also includes questions on guidelines and the current practices/tools to support feeding.

**Results:** In the workshop we will present the questionnaire and preliminary results from a pilot study. During the workshop, we will discuss findings from the pilot study and discuss the items included in the survey. Participants in the workshop will also be able to join the network and to use the survey in neonatal units, when relevant.

**Conclusion:** Development of a questionnaire to benchmark cue‐based feeding support with purpose of gaining knowledge of current strategies and practices to support positive feeding experiences for preterm infants.

**References**

Flacking, R., Tandberg, B. S., Niela‐Vilén, H., Jónsdóttir, R. B., Jonas, W., Ewald, U., & Thomson, G. (2021). Positive breastfeeding experiences and facilitators in mothers of preterm and low birthweight infants: a meta‐ethnographic review. International breastfeeding journal, 16(1), 1‐17.

Lubbe, W. (2018). Clinicians guide for cue‐based transition to oral feeding in preterm infants: An easy‐to‐use clinical guide. Journal of Evaluation in Clinical Practice, 24(1), 80‐88.

Maastrup, R., Hansen, B. M., Kronborg, H., Bojesen, S. N., Hallum, K., Frandsen, A., … Hallstrom, I. (2014). Factors associated with exclusive breastfeeding of preterm infants. Results from a prospective national cohort study. PLoS One, 9(2), e89077. https://doi.org/10.1371/journal.pone.0089077

McFadden, A., Fitzpatrick, B., Shinwell, S., Tosh, K., Donnan, P., Wallace, L. M., … Farre, A. (2021). Cue‐based versus scheduled feeding for preterm infants transitioning from tube to oral feeding: the Cubs mixed‐methods feasibility study.

Watson, J., & McGuire, W. (2016). Responsive versus scheduled feeding for preterm infants. Cochrane Database of Systematic Reviews(8).

## Writing the Second Edition of the World Health Organization's Infant and Young Child Feeding: Model Chapter for Textbooks for Medical Students and Allied Health Professionals

Julie Scott Taylor, MD, MSc^1^, Mariana Colmenares Castaño, MD, IBCLC^2^, Abla Alalfy, MD, FRCPCH, FRCP (UK), MRCP (UK), DCH, IBCLC (USA)^3^, Nina Chad, PhD^4^

^1^Adtalem Global Education, Chicago, USA. ^2^Centro de Lactancia y Alta Especialidad Pediatrica CLAP Hospital Médica Sur, Mexico City, Mexico. ^3^Galaa Military Complex, El Galaa Hospital for Armed Forces Officers Families, Cairo, Egypt. ^4^World Health Organization, Geneva, Switzerland

**Background:** As future healthcare professionals, medical, nursing, and midwifery students need high‐quality information about infant and young child feeding (IYCF) to be effective clinicians and health leaders. IYCF competencies for health professions require basic knowledge of a wide variety of maternal and child health topics, spanning from the molecular to the global.

**Methods:** The first edition of the WHO's Model Chapter, published in 2009, was used as a starting point. The year‐long project was divided into two main sections: focus groups and writing.

Six virtual focus groups were held in English in 2021 with 21 health care learners representing 8 nationalities. High‐level findings from the focus groups informed the writing stage of the project. A 12‐person international Steering Committee of experts from 11 countries met monthly. They worked in multidisciplinary writing teams to create overview documents and update sections, several of which were then independently by WHO content experts.

**Findings:** Medical and allied health students have limited knowledge of IYCF and breastfeeding. The majority of their training on these subjects is during clinical rotations, with a focus on clinical care of individual patients and families rather than health care systems. The current generation of medical students is very visual and fast‐paced. They learn via charts, algorithms, pictures, videos, and links rather than text.

The Second Edition of the WHO's Infant and Young Child Feeding: Model Chapter for Textbooks for Medical Students and Allied Health Professionals contains 11 learning objectives, 17 key points, nine detailed sections, a glossary, and links to current WHO resources. Each section is organized with key points, text, graphs/figures/tables/illustrations, links, and detailed references.

**Conclusion:** This document can be used during health training school by learners, including medical students and allied health professionals in nursing and midwifery, and by their teachers. Graduates who engage with this content will have the foundational knowledge required for the protection, promotion, and support breastfeeding at the individual, family, community and population health levels. Skills and competencies should follow.

**Reference**

Infant and Young Child Feeding: Model Chapter for Textbooks for Medical Students and Allied Health Professionals. Geneva: World Health Organization; 2009. PMID: 23905206.

## Investigation of breastfeeding Mother‐Infant dyad LaCtation process using Digital Infrared Thermal Imaging (MILC‐DITI): Proof‐of‐Concept

Anastasia Topalidou, Edward Sanderson, Lara McNally, Gill Thomson, Victoria Moran, Bogdan Matuszewski

University of Central Lancashire, Preston, United Kingdom

**Background:** Early and exclusive breastfeeding supports healthy development, improves cognitive performance (Krol & Crossmann, 2018), and can prevent more than 800,000 child deaths annually in children under five in the developing world (Victora, et al., 2016). Nevertheless, globally only 37%‐47% of babies <6 months are exclusively breastfed (Victora, et al., 2016) (WHO, 2021). Currently, there is little known about the vascular and thermal responses, and the mother‐infant heat exchange characteristics during breastfeeding (Chiu, Anderson, & Burkhammer, 2005). Such evidence could help provide better understanding of the maternal‐infant dyadic interaction during breastfeeding, and to help develop hypotheses about the role of physiological factors in breastfeeding complications. The aim of this proof‐of‐concept study was to evaluate the ability, validity, and feasibility of thermal imaging to record data related to lactation, milk flow, physiological alterations, and mother‐baby synchronicity.

**Methods:** The MILC‐DITI project proposal, and its methodological design were co‐produced with a Patients and Public Involvement and Engagement (PPIE) group, using two‐way processes. Three members of the PPIE group (breastfeeding mothers and their babies) were the study participants. Two of the dyads were recorded twice on separate days. After acclimatisation participants were recorded while breastfeeding with a thermal imaging radiometric camera. Static thermograms of the breasts were taken before and directly after breastfeeding. To facilitate analysis, a method was developed for tracking regions of interest and extracting temperature time‐series from the recordings. The thermograms were segmented to assess nipples’ temperature changes.

**Results:** Temperature time‐series were extracted for the mothers’ and babies’ cheeks, for which a correlation coefficient of 0.68 was found, suggesting potential synchronicity. Analysis of breast thermograms showed that the temperature of the breastfeeding nipple dropped by »1.0oC after the completion of breastfeeding, compared to the other nipple which did not exhibit notable change in temperature.

**Conclusion:** This is the first attempt to record dynamic thermal imaging data and use computer vision techniques to “decode” the maternal‐baby relationship during breastfeeding. This work showed that extraction of reliable temperature time‐series related data is possible. Larger number of data and longer periods are however needed to better facilitate the assessment of causal relationships.

## A Qualitative Investigation into Household Experiences and Coping Strategies for Food Insecurity

Ijeoma Chinyere Ukonu, Carol Ann Wallace, Nicola Lowe

University of Central Lancashire, Preston, United Kingdom

**Background:** A quantitative survey of household food insecurity conducted in Nsukka, Nigeria in 2019 revealed that 60% of households reported experiencing severe food insecurity (Ukonu, in prep). A follow‐up study has therefore been conducted to explore the experiences and coping strategies of households in this region.

**Methods:** The five most food‐insecure communities were selected for qualitative semi‐structured interviews. The district women's meeting executives were approached to identify 5 households in the food insecurity category. From this list, two women representing their households were randomly selected to give a total of ten (10) households. A semi‐structured interview topic guide (Lund, 2012) containing twenty questions exploring household experiences of food security and their coping strategies plus demographic information was piloted and adopted. Face‐to‐face interviews lasting an average of twenty‐five (25) minutes were conducted and recorded in July 2021. Data were organised through NVivo software and analysed using Thematic Network Analysis (Attride‐Stirling, 2016). Basic, organizing, and global themes were identified and discussed.

**Results:** Five global network themes emerged: household food insecurity experiences, household health and nutrition, causes and solutions to household food insecurity, household food insecurity coping strategies, and COVID‐19 lockdown. Additionally, 17 organising themes and 58 basic themes were identified. Findings revealed that food sufficiency and affordability rather than nutrition described household feeding. Most households rarely consume animal protein foods such as eggs, meat, fish, and milk. This raises questions about household nutrition, especially in children and immunocompromised household members. Fussy eating in children and poor intra‐household food distribution increased food insecurity. Safe food consumption was questionable due to food preservation practices. Some households involve the children in working or borrowing money for household food and income. Psychological experiences included worrying, sadness, insomnia, stigmatisation, and frustration. Coping strategies included food compromise, food assistance, reducing expenses, borrowing food and money, and food recycling.

**Conclusion:** Policies are urgently needed to establish food safety nets, and adequate grassroots intervention through agriculture, education, skill acquisition, and training from government, private and international partnerships.

**Keywords:** Coping strategies, Food security, Food insecurity, Household

**References**

Attride‐Stirling, J. (2016). Thematic networks: an analytic tool for qualitative research. Qualitative Research, 1(3), 385‐405. https://doi.org/10.1177/146879410100100307

Lund, T. (2012). Combining Qualitative and Quantitative Approaches: Some Arguments for Mixed Methods Research’. Journal of Educational Research, 56(2), 155‐165.

## Practical wisdom and ‘use of self’ to explain healthcare practitioners' confidence to provide breastfeeding support to the father

Maxine Wallis‐Redworth^1^, Gill Thomson^2^, Victoria Moran^2^

^1^Anglia Ruskin University, CAMBRIDGE, United Kingdom. ^2^University of Central Lancashire, Preston, United Kingdom

**Background:** Social support from family and friends has been highlighted as a key influence on the mother's feeding intention and breastfeeding initiation and continuation rates, with mothers particularly valuing the support from the father (Rempel and Rempel, 2011; Abbass‐Dick et al., 2015; Furman, Killpack, Matthews, Davis and O'Riordan, 2016). Training received by healthcare practitioners in the UK focuses on how to advise and support the breastfeeding mother; thus, the role of specifically supporting the father is inadequately addressed. This is an important consideration, as fathers report feeling overlooked, ignored, or excluded by healthcare practitioners and not receiving specific practical advice to undertake a supportive role to the breastfeeding mother (Brown and Davies, 2014).

**Methods:** A constructivist grounded theory study was undertaken with 13 healthcare practitioners working in midwifery and health visiting in the East of England. They were interviewed to explore their confidence in providing breastfeeding support to fathers.

**Findings:** The key findings identified that the healthcare practitioner's pre‐registration course, the UNICEF UK Baby Friendly training and annual employment organisational updates did not prepare them to provide breastfeeding support to the father. Confidence for this role was built through the acquisition of knowledge and experience from their professional and personal life and their personal attributes.

The findings generated the theory of ‘drawing on and engaging practical wisdom through the ‘use of self' to explain healthcare practitioners’ confidence to provide breastfeeding support to the father. In the absence of formal training, practitioners engaged practical wisdom drawing on knowledge and experience from personal and professional life combined with personal and professional attributes. They created a unique approach to each father by being able to tune into him and generate beneficial, apt, and even innovative decisions in the absence of any available support strategies and against the backdrop of organisational challenges they faced.

**Conclusion:** This research has identified the basis of healthcare practitioner confidence for breastfeeding support to fathers. More work is required to ensure that education/training and experience is available to all practitioners and is embedded into everyday practice to support breastfeeding goals of families.

**References**

Abbass‐Dick, J., Stern, S., Nelson, L.E., Watson, W. & Dennis, C.L. (2015). Coparenting breastfeeding support and exclusive breastfeeding: A randomized controlled trial. *Pediatrics*, 135(1), 102‐110.

Brown, A., & Davies, R. (2014). Fathers' experiences of supporting breastfeeding: challenges for breastfeeding promotion and education. *Maternal & Child Nutrition*, 10(4), 510‐526.

Furman, L., Killpack, S., Matthews, L., Davis, V., & O'Riordan, M. A. (2016). Engaging inner‐city fathers in breastfeeding support. *Breastfeeding Medicine*, 11(1), 15‐20.

Rempel, L.A., & Rempel, J.K. (2011). The breastfeeding team: The role of involved fathers in the breastfeeding family. *Journal of Human Lactation*, 27(2), 115‐121.

## Community perceptions and contextual considerations when collecting breastmilk samples: An experience from district Matiari of rural Sindh, Pakistan

Yaqub Wasan^1^, Jo‐Anna Baxter^2,3^, Azra Sheikh^1^, Abdullah Memon^1^, Sajid Soofi^1^, Deborah O'Connor^2,4^, Zulfiqar Bhutta^1,2,3^

^1^Aga Khan University, Karachi, Pakistan. ^2^Department of Nutritional Sciences, University of Toronto, Toronto, Canada. ^3^Centre for Global Child Health, Hospital for Sick Children, Toronto, Canada. ^4^Translational Medicine, Hospital for Sick Children, Toronto, Canada

**Background:** Data and specimen collection and analysis are critical components to understanding the relationship between breastmilk biology, breastfeeding practices, and infant health and development (Christian et al., 2021). However, depending on the context, sociocultural factors can influence a population's perception of breastmilk and breastfeeding (Zakar et al., 2018). This makes it equally important to understand and document the viewpoints and considerations that go into breastmilk specimen collection, including the use of customized procedures to address context‐specific perceptions.

**Methods:** We developed a nested‐substudy on breastmilk composition (ClinicalTrials. gov: NCT04451395) within the MaPPS Trial (ClinicalTrials. gov: NCT04451395) in rural district Matiari, Pakistan (Baxter et al., 2018). Breastmilk samples were collected by trained female staff between August 2021‐March 2022 from lactating mothers (17‐24 years) at one and/or 3 months post‐delivery. Qualitative notes were maintained to document preparatory steps, field experiences, and reflections regarding the community perceptions of breastmilk and breastmilk sample collection.

**Findings:** Unlike other biospecimens, breastmilk was thought to be very holy in the study setting. This is deeply rooted in the religio‐cultural values with breastmilk. Culturally, the dignity of a woman who breastfeeds is equally valued as a biological mother, and babies who are not breastfeed are considered ill‐fated. Furthermore, in adulthood, offspring are supposed to payback the great debt of breastfeeding to the mother, either in‐kind or cash. A mother has the right to wave the debt, or she can claim it on the day of resurrection. To facilitate breastmilk sample collection, we found enabling factors included approaching local religious leaders and community gatekeepers in advance to explain the study and provide additional information; allowing mothers time to involve their families and religious leaders in obtaining informed consent; identifying a private space for breastmilk expression; and providing information before sample collection to improve breastmilk volume recovery. Alternatively, major barriers included inflexible religious understanding of Pardah, wherein breasts are considered private parts of body, and perceived misuse of the collected breastmilk.

**Conclusions:** Culture‐specific considerations are important to conducting research ethically and responsibly. Promoting documentation of study implementation practices could substantially help investigators devise context‐specific procedures for future research.

**References**

Baxter, J. B., Wasan, Y., Soofi, S. B., Suhag, Z., & Bhutta, Z. A. (2018). Effect of life skills building education and micronutrient supplements provided from preconception versus the standard of care on low birth weight births among adolescent and young Pakistani women (15‐24 years): a prospective, population‐based cluster‐randomized trial. *Reproductive health*, 15(1), 104.

Christian, P., Smith, E. R., Lee, S. E., Vargas, A. J., Bremer, A. A., & Raiten, D. J. (2021). The need to study human milk as a biological system. *The American journal of clinical nutrition*, 113(5), 1063–1072.

Zakar, R., Zakar, M. Z., Zaheer, L., & Fischer, F. (2018). Exploring parental perceptions and knowledge regarding breastfeeding practices in Rajanpur, Punjab Province, Pakistan. *International breastfeeding journal*, 13, 24.

## Testing the validity of the International Complementary Feeding Evaluation Tool (ICFET) to detect disordered feeding and stability over time

Charlotte Wright^1^, Antonina Mutoro^2^, Ada L. Garcia^1^

^1^Human Nutrition dept, University of Glasgow, Glasgow, United Kingdom. ^2^African Population and Health Research Center, Nairobi, Kenya

**Background:** Measures of complementary feeding vary between settings and rarely assess child eating behaviour. We have developed a tool to assess diet diversity and frequency and eating behaviour in young children, valid for use in multiple languages. This has already been shown to have face validity and relate to growth outcomes in 572 6–24 months children in 6 countries. We next aimed to test its predictive validity to detect disordered feeding and its repeatability.

**Methods:** The ICFET was completed on two occasions in children (N = 30) attending a UK feeding clinic for tube weaning and/or severe feeding problems and in moderately malnourished children starting treatment in nutrition clinics in Kenya (N = 19) and Pakistan (N = 43).

The tool includes country specific food frequency table, questions on avidity and food refusal, generating scores for number of food groups eaten, avidity and food refusal expressed as SD scores (mean 0% and 95% range ±2) compared to a UK sample. The correlation of each score with a 2nd measure was used to assess stability over time.

**Results:** The UK Feeding clinic children were aged mean (range) 3.6 (1‐11) years and 47% had learning disability. They ate very few food groups (mean (SD) Z score −2.9 (1.6)) and had low avidity (−1.99 (1.4)) and high avoidance (1.8 (2.0)) z scores. All 3 scales showed moderate correlation between scale completions mean (SD) 43 (24) weeks apart: Avidity Spearmann's R = 0.59 *p* = 0.003, Refusal 0.72 *p* < 0.001, food frequency 0.66 *p* < 0.001. Only food frequency significantly increased by mean (SD) 0.52 (1.3) *p* = 0.049.

In both malnutrition clinics avidity scores were low and refusal scores high. When repeated after 1‐2 months they showed moderate correlation and refusal tended to decrease (see table).

**Table:** 

	Kenya			Pakistan		
		Mean (SD) Z score			Mean (SD) Z score	
	*R* ^1^	Baseline	Change	*R* ^1^	Baseline	Change
**Avidity**	0.52*	−1.8 ***(0.9)	+0.32*(0.6)	0.96**	−2.8*** (0.9)	−0.1** (0.27)
**Refusal**	0.55*	+0.86** (1.1)	−0.55* (1.1)	0.46**	+1.5*** (0.9)	−0.5*** (0.65)

P  < 0.001; ** < 0.01; * < 0.05. ^1^ *Spearmann's R*, time1 vs time2.

**Conclusion:** The ICFET identifies disordered eating behaviour and shows moderate stability over time. It should prove useful in future intervention studies and nutrition surveys.

## Posters

## Breastfeeding education and support for individuals with visual impairment: A scoping review protocol

Emma‐Rose Biggar^1^, Lisa McKenna^1^, Lisa H. Amir^2,3^

^1^School of Nursing and Midwifery, La Trobe University, Melbourne, Australia. ^2^Judith Lumley Centre, School of Nursing & Midwifery, La Trobe University, Bundoora, Australia. ^3^Breastfeeding service, Royal Women's Hospital, Parkville, Australia

**Background:** Women are encouraged to breastfeed, however, establishing breastfeeding is more than just bringing baby to breast. It is a learnt skill. Successfully establishing breastfeeding can be heavily influenced by the support families receive. Access to skilled support and accurate information has shown to increase breastfeeding exclusivity and duration (Gavine et al., 2022). Breastfeeding rates among women with disability or their support needs has not been explored in great depth (Mitra et al., 2015). Little is known about how mothers with visual impairment manage infant feeding.

The aim of this scoping review is to identify and map available evidence regarding the needs of mothers with visual impairment for initiating, establishing, and maintaining lactation/breastfeeding, and the supports and services currently available to meet those needs.

**Methods:** The scoping review will be conducted in accordance with the Joanna Briggs Institute methodology for scoping reviews and was registered with Open Science Framework on 7 September 2022 (Peters et al., 2022). An initial search of MEDLINE and Embase was undertaken to identify articles on the topic and develop a search strategy in consultation with a university librarian. This strategy, including identified keywords and index terms will be adapted for each database and information source as required. Following the database search, the table of contents in journals specific to breastfeeding will be explored. The websites of Australian organisations/agencies relevant to visual impairment will be examined. The reference lists of the selected studies will be screened for additional studies not previously identified. All sources of data in English will be included. No date range or geographical location will be applied. Identified articles will be uploaded to EndNote and Covidence for screening.

**Results:** The results of the full search and study inclusion process will be reported in the final scoping review using the PRISMA‐ScR flowchart (Tricco et al., 2018). Key themes will be presented with specific details about the participants, concept, context and key findings relevant to the aims of the review.

**Conclusion:** Exploring the experiences and needs specific to blind individuals wanting to breastfeed will guide future research and development of resources to enable access to effective support and education.

**References**

Gavine, A., Shinwell, S. C., Buchanan, P., Farre, A., Wade, A., Lynn, F., Marshall, J., Cumming, S. E., Dare, S., & McFadden, A. (2022). Support for healthy breastfeeding mothers with healthy term babies. Cochrane Database of Systematic Reviews 10:CD001141. https://doi.org/10.1002/14651858.CD001141.pub6

Mitra, M., Clements, K. M., Zhang, J., Iezzoni, L. I., Smeltzer, S. C., & Long‐Bellil, L. M. (2015). Maternal characteristics, pregnancy complications and adverse birth outcomes among women with disabilities. Medical Care, 53(12), 1027.

Peters, M. D. J., Godfrey, C., McInerney, P., Khalil, H., Larsen, P., Marnie, C., Pollock, D., Tricco, A. C., & Munn, Z. (2022). Best practice guidance and reporting items for the development of scoping review protocols. JBI Evidence Synthesis, 20(4):953‐968. https://journals.lww.com/jbisrir/Fulltext/2022/04000/Best_practice_guidance_and_reporting_items_for_the.3.aspx

Tricco, A. C., Lillie, E., Zarin, W., O'Brien, K. K., Colquhoun, H., Levac, D., Moher, D., Peters, M. D. J., Horsley, T., Weeks, L., Hempel, S., Akl, E. A., Chang, C., McGowan, J., Stewart, L., Hartling, L., Aldcroft, A., Wilson, M. G., Garritty, C., … Straus, S. E. (2018). PRISMA Extension for Scoping Reviews (PRISMA‐ScR): Checklist and Explanation. Annals of Internal Medicine, 169(7), 467‐473. https://doi.org/10.7326/M18-0850

## Market influence on breastfeeding observed across seven international studies (Indonesia, Vietnam, India, Thailand, Nigeria, Philippines and Mexico) impacting compliance of the WHO Code and relevant national regulations

Brenda K. Brewer^1^, Nadine Nasser^2^

^1^Westat, Rockville, MD, USA. ^2^Access to Nutrition Foundation, Utrecht, Netherlands

**Background:** Research indicates formula marketing has significant effect on breastfeeding. Mothers are less likely to exclusively breastfeed and more likely to breastfeed for shorter periods. Review of epidemiology and biology studies over three decades estimated that 832,000 child deaths and 20,000 breast cancer deaths could be saved in low‐ and middle‐income countries if breastfeeding increased (The Lancet Breastfeeding Series [TLBS], 2016). Children breastfed longer have fewer infections resulting in lower morbidity and mortality and higher intelligence (TLBS, 2016). In 2021, global baby food market was valued at >$70B. (Euromonitor International, 2022). Despite this, countries’ regulations controlling marketing varies considerably as documented in seven country assessments (Vietnam [2015], Indonesia [2015], India [2016], Thailand [2017], Nigeria [2017], Philippines [2020], Mexico [2020‐2021]).

**Methods:** Baby food companies’ compliance with the WHO Code, subsequent WHA resolutions, and relevant national regulations was assessed. Protocols followed included Interagency Group on Breastfeeding Monitoring (IGBM) in Vietnam, Indonesia, and India (IGBM, 2007), Protocol for the Assessment and Monitoring of “The Code” and Relevant National Measures (WHO, 2015) in Thailand and Nigeria, and Monitoring the Market of Breast‐milk Substitutes: Protocol for Periodic Assessment (WHO, 2017) for Philippines and Mexico. Assessments focused on marketing practices of six largest global baby food companies. Data included: exposure of women to breastmilk substitute marketing; promotional/educational materials and donated equipment observed in health facilities; marketing/promotions observed in selected retail and online stores; package labels and inserts; and traditional/online media monitoring.

**Findings:** Fewer incidences of noncompliance by country and company occurred with stricter local regulations. India's and Nigeria's national regulations go beyond the Code. Traditional media was most frequent source of marketing messages and more prevalent in Indonesia, Thailand and Mexico. More point‐of‐sale market promotions (including online) was observed in Indonesia, Thailand and Mexico.

**Conclusions:** Ever breastfed rates were similar ( ~ 95%) except in Mexico, and decreased at 1 year in countries with increased marketing (e.g., number media messages and point‐of‐sale promotions) (UNICEF, n.d.). India has the strongest regulations and highest rates of breast feeding at 1 year ( ~ 85%) and 2 years ( ~ 68%). Contact with mothers through social media is increasing across all countries.

**References**

Interagency Group on Breastfeeding Monitoring (IGBM). (2007). Monitoring compliance with the international code of marketing of breast‐milk substitutes: IGBM protocol for estimating the prevalence of violations of the code and national measures. London: IGBM.

Euromonitor International. (2022). Euromonitor International Passport. Retrieved from: https://www.euromonitor.com/our-expertise/passport

The Lancet Breastfeeding Series (TLBS). (2016). Breastfeeding in the 21st century: epidemiology, mechanisms, and lifelong effect. The Lancet, 387, 475‐490.

UNICEF. (n.d). Multiple Indicator Cluster Survey (MICS). Retrieved from: https://www.unicef.org/statistics/index_24302.html

World Health Organization (WHO). (2015). Protocol for the assessment and monitoring of “the code” and relevant national measures. Retrieved from: http://www.who.int/nutrition/netcode/protocol_summary.pdf

World Health Organization (WHO). (2017). Monitoring the marketing of breast‐milk substitutes: protocol for periodic assessment. Retrieved from: NetCode toolkit: monitoring the marketing of breast‐milk substitutes: protocol for periodic assessments (who. int).

## The evolution of online breastfeeding peer support

Nicole Bridges

Western Sydney University, Sydney, Australia

**Background:** In today's age of online communication and social media, one factor to be explored is the way social networking sites (SNSs) are being employed to provide breastfeeding peer support. One of the most interesting developments in social media is the rapid and continuous proliferation of Facebook. What began as a way to connect students, now boasts 2.934 billion (about one‐third of the world's population) monthly active users (Kemp, 2022) making it the world's most ‘active’ social media platform.

Traditionally, the Australian Breastfeeding Association (ABA) has provided information, education and peer support for breastfeeding mothers via their Breastfeeding Helpline, email counselling, online forums, and face‐to‐face at group meetings. In response to the COVID‐19 pandemic, the ABA and other breastfeeding support organisations have had to consider the role of Facebook and other SNSs, and step‐up their online presence as they endeavour to support, educate and engage breastfeeding parents well into the 21st century.

**Methods:** Employing an online ethnographic research approach, the aim of my research is to explore the experiences of users of closed Facebook groups attached to the ABA and how these parents seek and share breastfeeding support and information (Bridges, 2016; Bridges, Howell, & Schmied, 2018a, 2018b).

**Results:** In addition to showcasing how the ABA has responded to the COVID‐19 pandemic, I will explore the findings about the characteristics and contents of queries, shares, comments, and comment responses illustrating the ways in which parents not only gained access to support for their own breastfeeding experiences, but offered support to others.

**Conclusion:** It is evident that the ABA are providing a meaningful and valuable service to their members by supporting the use of SNSs in the form of closed Facebook groups for breastfeeding support and information. Furthermore, participation in such groups can be empowering for both new and more experienced parents and those who support them, including their families, health professionals, and breastfeeding peer supporters.

**References**

Bridges, N. (2016). The Faces of Breastfeeding Support: Experiences of mothers seeking breastfeeding support online. Breastfeeding Review, 24(1), 11‐20.

Bridges, N., Howell, G. V. J., & Schmied, V. (2018a). Breastfeeding peer support on social networking sites. Breastfeeding Review, 26(2), 17‐27.

Bridges, N., Howell, G. V. J., & Schmied, V. (2018b). Exploring breastfeeding support on social media. International Breastfeeding Journal, 13(22), 1‐9.

Kemp, S. (2022). Facebook Statistics and Trends. Retrieved from: https://datareportal.com/essential-facebook-stats

## “A very fun and messy journey”: An evaluation of the HENRY Starting Solids Workshop and its impact upon complementary feeding practices

Amy Brown, Sara Jones, Grace Hollinrake, Aimee Grant, Laura Wilkinson, Hannah Rowan

Swansea University, Swansea, United Kingdom

**Background:** The complementary feeding period when infants are introduced to solid foods is an important time, but one where parents often have lots of anxieties. The HENRY Starting Solids workshop is designed to prepare parents/carers to introduce solid foods to their baby and is free to attend.

**Methods:** We used a mixed methods longitudinal design to measure the impact of the workshop upon timing of solid foods, foods offered, responsive feeding and parental confidence. All participants who took part in a Starting Solids workshop between August 2020 and December 2021 were invited to take part. Participants completed a survey pre workshop (n = 867) and post workshop (n = 439) and another survey after starting solid foods with their baby (n = 58). We then conducted 11 interviews with mothers when their baby was 9 – 15 months old.

**Results:** Most participants had good knowledge around when to start solid foods, but many did not feel confident about the wider process of how to do this such as what foods to give, in what quantity and how to do so safely. The workshop improved knowledge and confidence which impacted upon feeding behaviours with parents attributing the workshop to what foods they offered their baby, feeding styles and safety considerations. Examples of impact included recalling messages such as introducing more bitter tasting vegetables as first foods, trusting a baby to set the pace of the meal, and remembering that food at this age is as much of a learning experience as it is about amounts consumed. However, baby snack foods did still play a role, usually for convenience with parents viewing them as different to ‘adult’ snacks.

**Conclusion:** When we think about ‘starting solids’ a lot of focus is often placed upon timing of first foods with many studies using it as a key outcome measure. Timing is of course important and associated with later health outcomes, but it is important to remember that the experience of starting solids is broader than this. The complementary feeding period is a time where taste foundations can be laid, babies encouraged to follow their own internal cues and family mealtimes encouraged.

**References**

Scientific Advisory Committee for Nutrition. Feeding in the first year of life. 2018. London

Arden, M.A. (2010). Conflicting influences on UK mothers’ decisions to introduce solid foods to their infants. Maternal & child nutrition, Apr;6(2):159‐73.

Brown, A., Jones, S.W., Hollinrake, G., Grant, A, Wilkinson, L & Rowan, H. (2022). “A very fun and messy journey” An evaluation of the HENRY Starting Solids Workshop and its impact upon complementary feeding practices. HENRY: Oxfordshire

Brown, A., & Rowan H. (2016). Maternal and infant factors associated with reasons for introducing solid foods. Maternal & child nutrition, Jul;12(3):500‐15.

Schwartz, C., et al., (2011). Development of healthy eating habits early in life. Review of recent evidence and selected guidelines. Appetite, Dec 1;57(3):796‐807.

## Effect of overweight on the energy value of human milk from donors from a human milk bank in Brazil

Lucíola Sant'Anna de Castro^1^, Rebeca de Freitas Paiva^1^, Ana Carolina Lavio Rocha^1^, Barbara Tideman Sartorio Camargo Camargo^1^, Ana Carolina Prima de Souza Souza^1^, Adriana Rosa Carneiro^2^, Cristiana Araújo Guiller Ferreira^1^, Adriana Souza Torsoni^3^, Michael G. Ross^4^, Mina Desai^4^, Bernardo Lessa Horta^5^, Kelly Pereira Coca^1^

^1^Universidade Federal de São Paulo, São Paulo, Brazil. ^2^Banco de Leite Humano/Centro Ana Abrão ‐ UNIFESP, São Paulo, Brazil. ^3^Universidade de Campinas, Campinas, Brazil. ^4^The Lundquist Institute at Harbor‐UCLA, Los Angeles, USA. ^5^Universidade Federal de Pelotas, Pelotas, Brazil

**Background:** For premature babies and with low birth weight, who do not have their own mother's milk available, pasteurized HM from donors is recommended as the first option for their feeding. Maternal nutritional status is associated with changes in fat and lactose concentrations in Human Milk (HM) (Leghi et al., 2020). The aim of our study was to evaluate the nutritional status of donors and the energy value of their human milk.

**Methods:** Cross‐sectional study with donors from the Human Milk Bank at Breastfeeding Center in São Paulo, Brazil. Maternal sociodemographic and anthropometric data were collected from the medical records. Body Mass Index (BMI) were calculated to assess nutritional status and the HM energy value was determined by the Creamatocrit method. We classified the value of calorie as normocaloric HM less than <700Kcal/L and hypercaloric more than 700Kcal/L. The Chi‐square statistics and Fisher's exact test and Student's t test were used for data analysis.

**Results:** 279 donors were included, with a mean age of 32.6 years (SD = 5.3), most with higher education (73.8%), and with a partner (87.8%). Regarding nutritional status, 53.4% were eutrophic, 31.9% overweight and 14.7% obese women. The average caloric (Kcal/L) of donated HM was 598.6 Kcal (SD = 148.3), most of which were classified as normocaloric (52.1%) followed by hypercaloric (20.5%). When associated with the HM fat content and the nutritional status of donors, it was observed that the mean HM fat content of overweight donors is higher when compared to that of eutrophic women (*p* = 0.048).

**Conclusions:** Overweight/obese women had a higher frequency of HM classified as hypercaloric and with a higher fat content. BMI composition in HM should be considered given its influence on the nutritional status of babies and health conditions throughout life.

Leghi, G., Netting, M., Middleton, P., Wlodek, M., Geddes, D. & Muhlhausler, B. (2020) The Impact of Maternal Obesity on Human Milk Macronutrient Composition: A Systematic Review and Meta‐Analysis. *Nutrients*, 12(4):934.

## Impact of Maternal Nutritional Status of the Nutritional Composition of Human Milk and Infant Growth

Lucíola Sant'anna De Castro^1^, Ana Carolina Lavio Rocha^1^, Bárbara Tideman Sartorio Camargo^1^, Ana Carolina Prima de Souza^1^, Adriana Souza Torsoni^2^, Marcio Alberto Torsoni^2^, Bernardo Lessa Horta^3^, Michael el Gross^4^, Mina Desai^4^, Kelly Pereira Coca^1^

^1^Universidade Federal de São Paulo, São Paulo, Brazil. ^2^Universidade de Campinas, Campinas, Brazil. ^3^Universidade Federal de Pelotas, Pelotas, Brazil. ^4^The Lundquist Institute at Harbor‐UCLA, Los Angeles, USA

**Background:** The human milk (HM) composition is dynamic and may vary according to a variety of factors related to mother (Bzikowska‐Jura et al., 2020). The aim of this study was to investigate the relationship between maternal nutritional status and the composition of HM.

**Methods:** We prospectively followed breastfeeding women in the first and sixth month postpartum at a Breastfeeding Center in São Paulo, Brazil. We collected maternal sociodemographic data by interview. Anthropometric, body composition (bioelectrical bioimpedance), HM (electric pump) and milk analysis (Miris®) at one and 6 months postpartum. Nutritional status was classified according to the pre‐gestational Body Mass Index (BMI) as eutrophic (18‐24.9) vs OW/OB (>25). Body composition (BC) was classified as P50 of the sample distribution (%BC ≤32,3 vs %BC >32,3). Statistical comparisons were performed with chi‐square and paired and unpaired t‐test.

**Results:** 53 breastfeeding women, 56.6% eutrophic and 43.4% OW/OB, with a mean age of 31.7 ± 5.4 years were followed. Eutrophic women presented the mean of weight gain during pregnancy (13.3 vs 8.5Kg; *p* = 0.009) higher than the OW/OB, but they lost more weight (3.1 kg) compared to OW/OB who gained weight (0.5Kg). OW/OB women had higher fat mass (*p* = 0.018) from the months postpartum and their average HM calories and percentage of body fat (*p* = 0.043) was higher in the first month. The fat content of HM increased in both eutrophic (5.4 to 6.3 g; *p* = 0.091) and OW/OB women (5.9 to 6.5 g; *p* > 0.05) at the period, without statistical significance. When evaluating infant weight gain and maternal body composition, we observed that girls whose mothers had a higher percentage of body fat had greater weight gain at 6 months (*p* = 0.009).

**Conclusions:** The increase in maternal body fat may influence the energy content of HM with repercussions on the weight gain of female babies, contributing to the generational cycle of obesity.

Bzikowska‐Jura A, Sobieraj P, Szostak‐W˛egierek D & Wesołowska A. (2020). Impact of Infant and Maternal Factors on Energy and Macronutrient Composition of Human Milk. Nutrients, 12,(2591), 1‐14. https://doi.org/10.3390/nu12092591

## Complex Case Management Techniques: Case Review of Stacked Rare Diseases

Darby Dickton^1^, Jimi Francis^2^

^1^The Foundation for Maternal Infant Lactation Knowledge, San Antonio, USA. ^2^University of Texas ‐ San Antonio, San Antonio, USA

**Background:** As providers, we have a duty to do the best possible by every patient, including cases that may feel overwhelming. This case challenged every team member in many ways, and while such a presentation is unlikely to occur again, there are many things to be learned from such a case. This case review will examine the journey of a 31‐year‐old primigravid female with Ehlers Danlos Syndrome (EDS)(Karthikeyan & Venkat‐Raman, 2018), a rare connective disease, and Chronic Inflammatory Demyelinating Polyneuropathy (CIDP), a rare autoimmune neurologic condition managed with weekly plasmapheresis via port access(Manley & Geary, 2017).

**Findings:** During a screening echocardiogram in her first trimester, she was found to have a large right catheter‐related atrial thrombus (CRAT) and immediately started on anticoagulation (Yang et al., 2018). The pregnancy was challenged by malnutrition and weight loss. In the third trimester, the patient identified a right breast mass with rapid growth. On imaging and biopsy, this mass was further classified as a giant phyllodes tumor. The patient was induced and had an unmedicated vaginal delivery. The delivery was complicated by a primary post‐partum hemorrhage from retained placenta and a delayed hemorrhage 3 weeks later from the rare complication known as Subinvolution of the Placental Site (SPS)(Recognition and Management of Secondary Postpartum Hemorrhage ‐, n.d.). The infant was born with a class 4 tongue tie which was clipped at 3 days old but was otherwise healthy and exclusively provided breastmilk from birth.

**Conclusion:** This case was fraught with medical dilemmas and difficult choices. The comorbidities during pregnancy gave every choice a level of unquantifiable risk, and in the post‐partum period the hemorrhages complicated the picture as well as slowed down milk production. Here we will specifically explore the pregnancy and lactation considerations, related treatment decisions, and how care could have been improved.

**References**

Karthikeyan, A., & Venkat‐Raman, N. (2018). Hypermobile Ehlers–Danlos syndrome and pregnancy. Obstetric Medicine, 11(3), 104. https://doi.org/10.1177/1753495X18754577

Manley, C., & Geary, F. (2017). An Incidence of Chronic Inflammatory Demyelinating Polyneuropathy in Pregnancy [28 L]. Obstetrics & Gynecology, 129(1), S128–S129. https://doi.org/10.1097/01.AOG.0000514659.74781.24

Recognition and management of secondary postpartum hemorrhage ‐. (n.d.). Retrieved September 25, 2022, from https://www.npwomenshealthcare.com/management-of-secondary-postpartum-hemorrhage/

Yang, H., Chen, F., Jiao, H., Luo, H., Yu, Y., Hong, H. G., Li, Y., Fu, P., & Cui, T. (2018). Management of tunneled‐cuffed catheter‐related right atrial thrombosis in hemodialysis patients. Journal of Vascular Surgery, 68(5), 1491–1498. https://doi.org/10.1016/J.JVS.2018.02.039

## Increases in systolic and diastolic blood pressure during pregnancy are associated with vitamin D and magnesium deficiency in early pregnancy

Jimi Francis^1^, M. Naowar^2^, J. Baxter^2^, P. Flynn^2^ and Darby Dickton^3^

^1^Assistant Professor, College of Health, Community, and Policy, University of Texas at San Antonio, San Antonio, Texas, USA. ^2^Student, University of Texas at San Antonio, San Antonio, Texas. ^3^Medical Director, Foundation for Maternal, Infant, and Lactation Knowledge, San Antonio, Texas, USA

**Background:** The study aimed to evaluate vitamin D and magnesium during pregnancy to determine any associations with systolic and diastolic blood pressure changes.

**Methods:** Women were recruited in their first trimester of pregnancy. The sample size calculation was based on a confidence interval of 20 and a confidence level of 95%. Measurements were collected at 12‐, 20‐, and 30 weeks’ gestation. Anthropometric measurements included height, weight, wrist and ankle circumference, and blood pressure. Serum was tested for vitamin D, magnesium, C‐reactive protein, and erythrocyte sedimentation rate (ESR). Weight and height were measured to obtain Body Mass Index (BMI). Blood pressure and circumferences were also measured. Descriptive statistics, t‐tests, chi‐square, and analysis of variance were used to analyze the data collected.

**Results:** Twenty‐five participants completed the study. At 12 weeks gestation, 32% were clinically deficient in vitamin D, and 36% were clinically deficient in magnesium. Of the total participants, 20% were deficient in both vitamin D and magnesium. Those deficient in vitamin D and magnesium had high systolic and diastolic blood pressure at 30 weeks of gestation.

**Conclusions:** Vitamin D and magnesium are crucial nutrients for proper vascular system function. Deficiencies of vitamin D and magnesium are associated with increased systolic and diastolic blood pressure. Correcting deficiency of these nutrients in early pregnancy may reduce the risk of increases in systolic and diastolic blood pressure in late pregnancy. Using vitamin D and magnesium to evaluate the potential risk of developing high blood pressure during pregnancy may be a viable way of preventing increases in blood pressure. The results of this study warrant further investigation with a larger sample size. Funding Sources Partial funding for this project was received from the Foundation for Maternal, Infant, and Lactation Knowledge.

## Transition to motherhood during COVID‐19 in Croatia: Survey of new mothers to evaluate hospital practices

Irena Zakarija‐Grkovic^1^, Gill Thomson^2^, Daniela Drandic^3^, Anita Pavicic Bosnjak^4^, Ana Vidovic Roguljic^5^, Lisa Amir^6^

^1^University of Split School of Medicine, Split, Croatia. ^2^University of Central Lancashire, Preston, United Kingdom. ^3^Roda ‐ Parents in Action, Zagreb, Croatia. ^4^University Hospital Centre Zagreb, Zagreb, Croatia. ^5^Split Dalmatia County Community Health Centre, Split, Croatia. ^6^La Trobe University, Melbourne, Australia

**Background:** The Baby‐friendly Hospital Initiative (BFHI) was fully implemented in all 31 public maternity hospitals in Croatia by 2016. In 2018, results on implementation of the BFHI ‘Steps’ 3‐9 among healthy, term maternal‐infant dyads in a large, tertiary maternity facility were published (Zakarija‐Grkovic et al., 2018). The recent COVID‐19 pandemic has caused disruptions to most areas of health care (Moynihan et al., 2021), and the World Health Organization calls for research to investigate how the pandemic influenced the provision of care in maternity facilities (Ahmed et al., 2021). Hence, our aim was to investigate the adherence to BFHI standards in Croatian maternity hospitals during the COVID‐19 pandemic, and to compare the findings with those collected during the previous study

**Methods:** A cross‐sectional study was conducted via an online questionnaire between February and April 2022 among resident Croatian women who had enrolled in the RODA (‘Parents in Action') online antenatal course. Women aged 18+years, who had given birth in a Croatian maternity facility between February 2020 and December 2021 were invited to participate. The questionnaire contained 75 items, covering sociodemographic characteristics, hospital practices, community support, maternal mental and physical health.

**Results:** 3289 responses were received, of which 2130 were complete. To compare our findings with our earlier study, mothers of preterm infants (n = 120) were removed for this analysis, bringing the total to 2010. In pregnancy, 1471 (82%) of women did not receive any information from their health care providers on: breastfeeding, the importance of skin‐to‐skin contact, ‘rooming‐in’ or risks of formula supplementation. Initiation of breastfeeding within 1 h of birth improved (42%; n=851vs 31% pre‐pandemic), as did skin‐to‐skin contact for at least an hour (39%; n = 776 vs 23% pre‐pandemic). The proportion of participants who received breastfeeding help within 6 h of birth were lower, 27% (n = 536), compared to 69% pre‐pandemic. Fewer participants reported ‘rooming‐in’: only 43% (n = 869) were with their baby both day and night, compared to 99% pre‐pandemic. During the first 2 days after birth, 72% (n = 1456) of healthy term newborns received something other than human milk.

**Conclusion:** In‐hospital breastfeeding support in Croatia decreased during the pandemic, consistent with a study from Spain (Muñoz‐Amat et al., 2021). Reports from mothers indicate high levels of in‐hospital supplementation and marked lowering of BFHI standards in Croatian maternity hospitals during the pandemic.

**References**

Ahmed, N. U., Maamri, A., Falade, A. G., Ayede, A. I., Bhutta, A., Gambhir, A., … Navabi, Z. S. (2021). Global research priorities on COVID‐19 for maternal, newborn, child and adolescent health. Journal of global health, 11, 04071. https://doi.org/10.7189/jogh.11.04071

Moynihan, R., Sanders, S., Michaleff, Z. A., Scott, A. M., Clark, J., To, E. J., … Albarqouni, L. (2021). Impact of COVID‐19 pandemic on utilisation of healthcare services: a systematic review. BMJ open, 11(3), e045343. https://doi.org/10.1136/bmjopen-2020-045343

Muñoz‐Amat, B., Pallás‐Alonso, C. R., & Hernández‐Aguilar, M. T. (2021). Good practices in perinatal care and breastfeeding protection during the first wave of the COVID‐19 pandemic: a national situation analysis among BFHI maternity hospitals in Spain. International breastfeeding journal, 16(1), 66. https://doi.org/10.1186/s13006-021-00407-y

Zakarija‐Grković, I., Boban, M., Janković, S., Ćuže, A., & Burmaz, T. (2018). Compliance With WHO/UNICEF BFHI Standards in Croatia After Implementation of the BFHI. Journal of human lactation: official journal of International Lactation Consultant Association, 34(1), 106–115. https://doi.org/10.1177/0890334417703367

## The feeding protocol *10% per day*: A pilot study

Linn J Gustavsen^1,2^, Cecilie D Evensen^2^, Ingrid H Ravn^1^, Kjersti Sonning^3^, Bente S Tandberg^2,4^

^1^Oslo Metropolitan University, Oslo, Norway. ^2^Vestre Viken Hospital Trust, Drammen, Norway. ^3^Oslo University Hospital, Oslo, Norway. ^4^Lovisenberg Diaconal University College, Oslo, Norway

**Background:** To ensure adequate nutritional requirements for the preterm infant, recommended amount of fluid for stable growing infants will require intakes of 150‐180 ml/kg (Embleton et al., 2022). Due to the preterm infant immaturity, a feeding tube is usually required to meet these needs (Lubbe, 2018). Several neonatal intensive care units in Norway are using a feeding protocol to enhance full oral feeding. The protocol, called *10% per day*, describes a reduction of the enteral nutrition to 100 ml/kg/day. This is an experience‐based strategy and there is no previous evidence of how it affects the infants process to full oral feeding.

**Purpose:** To describe time to attain full oral feeding, how many trials of the protocol were used and breastfeeding rates at discharge, as well as how the protocol was used according to guidelines and infants weight gain during the protocol.

**Method:** A quantitative retrospective observative descriptive pilot study was conducted in a level 3a Nicu in Norway. Data was collected from electronic charts over a twelve‐week period. Descriptive analysis was performed with the use of SPSS®.

**Results:** Twelve preterm infants born before 34 weeks of gestational age were included. The infants used an average of 9 days from the start of the protocol to attain full oral feeding. Half of the infants needed more than one attempt with the protocol. Eight infants were exclusively breastfed at discharge. Practice shows several aberrations from the protocol and the results suggests that the weight gain was negatively affected.

**Conclusion:** This pilot study shows several aberrations from the protocol and suggest the need of a thorough review. A study of a larger scale is needed to determine whether the protocol is enhancing full oral feeding as intended, especially in regard to the nonoptimal weight gain. This pilot study is the first to describe the protocol and can contribute to a discussion on the approaches to enhance breastfeeding in the preterm infant.

**Reference**

Embleton, N. D., Moltu, S. J., Lapillonne, A., van den Akker, C. H. P., Carnielli, V., Fusch, C., … Domellöf, M. (2022). Enteral Nutrition in Preterm Infants (2022): A Position Paper from the ESPGHAN Committee on Nutrition and invited experts. Journal of Pediatric Gastroenterology and Nutrition. https://doi.org/10.1097/mpg.0000000000003642

Lubbe, W. (2018). Clinicians guide for cue‐based transition to oral feeding in preterm infants: An easy‐to‐use clinical guide. Journal of evaluation in clinical practice, 24(1), 80‐88.

## What do we know about parents’ experiences and needs when it comes to mixed feeding: A systematic review

Karen Hall, Amy Brown, Sara Jones

Swansea University, Swansea, United Kingdom

**Background:** We know that although intention to breastfeed is high, many families make the decision to combine breastfeeding with use of formula to feed their baby. In the last infant feeding survey whilst 23% of babies were exclusively breastfed at 6 weeks, 55% were getting at least some breastmilk at this time, indicating a high rate of partial breastfeeding. However many parents feel unsupported with mixed feeding, often stopping any breastfeeding because they believe it unnecessary or have not been shown how to combine breast and bottle feeding. There is little research on these experiences and how to better support mixed feeding.

**Methods:** A systematic review was conducted to identify parents’ experiences and needs when it comes to mixed feeding their baby. Initial literature searches focused on parents’ experience and expectations of mixed feeding, and excluded studies related to health outcomes, specific health contexts such as HIV and gestational diabetes. Papers were limited to settings where mixed feeding takes place in a similar cultural setting to the UK.

**Findings:** Several themes were identified which illustrated why parents choose to introduce formula milk alongside breastfeeding. For some this is based on a perception that while breastfeeding is “best” for babies, it is unsustainable, and formula use is inevitable. Notably, parents feel unsupported to balance breast and formula milk, and social and cultural narratives about morality in infant feeding leave them feeling judged or criticised in their decision.

Overall there is little published directly about parents’ intentions and experiences of mixed feeding. Much of the data about mixed feeding is ‘hidden’ within broader breastfeeding research, and focuses on parents’ feelings when feeding does not go to plan. The findings indicate a lack of understanding of how parents decide to start mixed feeding, how they undertake it, whether it does alleviate those challenges and where they get support and information.

**Conclusion:** For parents who wish to, or need to mix feed, there is a need for research that can provide accessible information about how mixed feeding can work, enabling them to maximise the mixed feeding period and meet their own feeding goals.

**References**

Brown, A. (2016). What do women really want? Lessons for breastfeeding promotion and education. *Breastfeeding medicine*, *11*(3), 102‐110.

Burton, A., Taylor, J., Swain, S., Heyes, J., Cust, F., & Dean, S. (2022). A qualitative exploration of mixed feeding intentions in first‐time mothers. *British Journal of Midwifery*, *30*(1), 20‐29.

McAndrew, F., Thompson, J., Fellows, L., Large, A., Speed, M. & Renfrew, M. (2012). *Infant feeding survey 2010.* Health and Social Care Information Centre. https://sp.ukdataservice.ac.uk/doc/7281/mrdoc/pdf/7281_ifs-uk-2010_report.pdf

Papadopoulos, N. G., Balan, T. A., van der Merwe, L. F., Pang, W. W., Michaelis, L. J., Shek, L. P., Vandenplas, Y., Teoh, O. H., Fiocchi, A. G., & Chong, Y. S. (2022). Mixed Milk Feeding: A New Approach to Describe Feeding Patterns in the First Year of Life Based on Individual Participant Data from Two Randomised Controlled Trials. *Nutrients*, *14*(11), 2190. https://doi.org/10.3390/nu14112190

## Caregiver Training Results in Accurate Reporting of Crying Episodes

Matt Heidman^1^, Susan Dallabrida^2^, Analice Costa^2^

^1^SPRIM US LLC, Orlando, USA. ^2^SPRIM US LLC, Boston, USA

**Background:** Accuracy of caregiver reported outcomes is essential for infant growth and tolerability study success. Crying/fussiness, stool consistencies and other gastrointestinal characteristics are important parameters regarding tolerability and inter‐caregiver reporting can see a significant amount of subjectivity and vary greatly within an infant growth study, compromising data. This study sought to elucidate how caregiver reported questions related to crying episodes are answered before and after a short amount of training and how training impacts caregivers’ understanding and how they would answer the question.

**Methods:** A digital survey was issued for 90 days in the US (n = 121) and 30 days in Mexico (n = 88) targeting respondents with children ≤4 years of age. Respondents were asked a question in two formats, first without a line of training text and second with a line of training text. The question set was as follows, “If your baby were crying for 10 min and stopped crying for 3 min and then started to cry again for 2 min, how many episodes of crying would you report this as?” followed by the same question beginning with “If you were given the instruction that an episode of CRYING stops when there is a period of contentment that lasts more than 5 min…”. Four response items were provided for both questions, 1) 1 episode, 2) 2 episodes, 3) It depends on how severe the crying was at each point in time, 4) There is not enough information to be able to answer the question. Response frequencies between questions were compared.

**Results:** Responses to the question without training saw high amount of variability in the US with 25% selecting “1 episode”, 42% selecting “2 episodes”, 16% selecting “It depends on how severe the crying was at each point in time”, and 17% selecting “There is not enough information to be able to answer the question” and in Mexico respondents selected 18%, 52%, 20% and 9% respectively. However, responses to the question after training saw more consolidation in the US, with 73% of respondents selecting “1 episode” representing a 2.9 fold increase in those selecting the correct answer. Additionally in Mexico, with 42% of respondents selecting “1 episode” resenting a 2.3 fold increase.

**Conclusions:** Caregiver reported outcomes are critical for infant growth and tolerability studies, however, can be highly subjective and see a high variability of responses without guidance. Training is critical to standardize all caregivers’ perspective regarding how to answer questions accurately to provide a sound data set.

## High Interest in Infant Related Decentralized Clinical Trial Participation Among Caregivers

Matt Heidman^1^, Susan Dallabrida^2^, Analice Costa^2^

^1^SPRIM US LLC, Orlando, USA. ^2^SPRIM US LLC, Boston, USA

**Objective:** High participant burden in clinical trials has been a notorious issue among standard site based clinical trials, especially among caregivers participating in infant growth and tolerability studies. Study participation requirements such as frequent site visits and doctor evaluations for infant growth result in poor study protocol adherence and high dropout rates, negatively impacting study data and results. This study sought to elucidate infant caregivers’ stance and sensitivities regarding clinical trial participation and their inclination toward decentralized clinical trials (DCT).

**Methods:** A digital survey was issued for 90 days in the US (n = 121) and 30 days in Mexico (n = 88) targeting respondents with children ≤4 years of age. Regarding study visits and vital data collection, respondents were asked which they would prefer between, 1) nurse visits to their home, 2) doctor visits, or 3) learning how to take their infant's vital signs at home. Regarding use of measurement devices for data collection, respondents were asked what they would prefer between, 1) being sent devices and do everything at home or 2) doctor visits. Lastly, regarding how to be instructed on device use, respondents were asked which they would prefer between 1) Reviewing a “how to do” digital training video on their smartphone, 2) Reviewing written instructions, or 3) a telehealth video call with a nurse.

**Results:** In the US 78% stated they would rather learn how to take their infant's vital signs at home, 88% stated they would rather be sent devices and do everything at home, and 55% stated they would want to review a “how to do” digital training video on their smartphone when learning how to use a study device. In Mexico respondent frequencies were 78%, 64% and 60% respectively.

**Conclusions:** Among infant caregivers in North America, there is a clear interest and strong inclination toward participating in DCTs and learning and executing study procedures from the home over traditional clinical trial procedures. By utilizing a DCT model in infant growth and tolerability studies, investigators have the potential to decrease participant burden, increase satisfaction and improve participant retention and data quality.

## Experience, skills and attitudes of multi‐disciplinary paediatric professionals supporting breastfed medically complex children

Lyndsey Hookway, Amy Brown, Janice Lewis

Swansea University, Swansea, United Kingdom

**Background:** Breastfeeding may be particularly beneficial for medically complex children (Hassiotou and Geddes, 2015; Theurich et al., 2021). However, childhood illness is associated with additional challenges and lower breastfeeding rates. The Baby Friendly Initiative has been shown to improve health professional skills although the standards have not yet been adopted in paediatrics and most professionals defer to personal experience (Pound et al., 2014; Baker et al., 2021; Boss et al., 2021).

There are no previous multidisciplinary studies of breastfeeding knowledge and attitudes in paediatrics, thus this survey was conducted to establish their confidence, barriers and skills supporting breastfeeding.

**Methods:** The online survey recruited paediatric healthcare professionals from November 2020‐March 2021. Questions about attitudes to breastfeeding, training, credentials, barriers and ward culture were included with Likert scales and 409 professionals were included in the analysis.

**Results:** A total of 245 paediatric nurses, 103 doctors, 45 allied health professionals and 16 health care assistants completed the survey. Most professionals (70.4%) had no breastfeeding training, 16% had completed 1‐3 days of training and 13% had extensive training (peer supporter, breastfeeding counsellor or IBCLC). Many (93.3%) felt that different skills are required in paediatrics and professionals noted that standard breastfeeding training focuses on establishing breastfeeding in healthy newborns rather than more complex cases.

Participants were asked about 13 clinical competencies and an aggregate skill score was calculated. Multiple univariate analysis of variance (ANOVA) found that more extensive training was correlated with higher skill scores (*p* < 0.001). There were many barriers to providing support including lack of knowledge and the need to measure fluid balance, and relatively little awareness of support to refer to, especially among those with lower skill levels (*p* < 0.001).

**Conclusion:** This national sample of professionals nevertheless had both basic and more specialised knowledge gaps and training needs. Medically complex children encounter many barriers to optimal feeding ‐ including absence of paediatric lactation staff, and may have additional challenges such as re‐establishing oral feeding after ventilation or enteral/parenteral feeding. Skill deficits identified in this study indicate that existing training is insufficient, and bespoke paediatric breastfeeding training based on identified clinical challenges is justified.

## Barriers and facilitators of breastfeeding and breast milk feeding amongst parents with an infant with cleft lip and/or palate: A mixed methods systematic review

Kan Man Carmen Li

King's College London, London, United Kingdom

**Introduction:** Cleft lip and/or palate is the most common craniofacial abnormality, affecting 1 in 700 infants in the United Kingdom (UK). Breastfeeding infants with cleft lip and/or palate is challenging and will likely require assistance. There is limited literature into breastfeeding outcomes of cleft lip and/or palate infants, leading to policymakers having a challenging time making breastfeeding recommendations (Boyce et al., 2019).

**Aim:** To explore the barriers and facilitators of breastfeeding amongst parents with an infant with cleft lip and/or palate.

**Methods:** A systematic search was conducted on seven databases (CINAHL, MEDLINE, Embase, PsycINFO, Global Health, Web of Science and Scopus) and grey literature platforms. Eligibility criteria included studies involving parents/carers of a child with cleft lip and/or palate exploring aspects of breastfeeding and breast milk provision. The guideline for mixed‐methods systematic reviews by the Joanna Brigg's Institute was followed. Data were extracted from included studies and analysed using a convergent integrated approach, where quantitative data was transformed into a qualitative format. Each included study was quality appraised using a JBI checklist appropriate to the study's research design.

**Results:** Parents expressed breastfeeding as a challenging experience. Barriers of breastfeeding and breastmilk provision involved experiencing breastfeeding and expressing difficulties, mixed infant feeding advice from healthcare professionals. Many parents reported needing to use specialised feeding bottles or feed via an enteral feeding device. Some women did not attempt breastfeeding and opted for formula feeding. Facilitators of breastfeeding and breastmilk provision included support from partners, specialist cleft healthcare professionals and online support groups.

**Conclusions:** Optimising breastfeeding and breastmilk provision for infants with cleft lip and/or palate is essential for the infant's health and strengthening parent‐infant bonding. Specialised support from healthcare professionals is paramount to optimising breastfeeding and breastmilk provision. Further research is required into interventions to support parents with breastfeeding and breastmilk provision.

## COVID‐19 response and breastfeeding related policy among a cohort of US birthing hospitals

Anne Merewood^1^, Laura Burnham^2^, Jacqueline Berger^3^, Aishat Gambari^4^, Nathan Nickel^5^

^1^Boston University School of Medicine, Boston, USA. ^2^Boston Medical Center, Boston, USA. ^3^Georgia Southern University, Statesboro, USA. ^4^Emory University, Atlanta, USA. ^5^University of Manitoba, Manitoba, Canada

**Background:** During the COVID‐19 pandemic, patient care recommendations for U.S. birthing hospitals were in flux and caused disruptions in skin‐to‐skin practices, lactation support, and postpartum discharge, all of which can affect breastfeeding initiation and duration. In Mississippi, 37/41 birthing hospitals participate in the Communities and Hospitals Advancing Maternity Practices (CHAMPS) program, which helps hospitals implement the Ten Steps to Successful Breastfeeding, leading to significant increases in hospital breastfeeding rates from 2014 to 19. Access to data from this cohort allowed us to compare breastfeeding and maternity care practice rates in enrolled hospitals before and during the COVID‐19 pandemic, and to assess changes in maternity care policies in response to the pandemic.

**Methods:** Enrolled hospitals submitted monthly data on any and exclusive breastfeeding, skin‐to‐skin care, and rooming‐in. We assessed hospital policy adaptation during the pandemic using an online survey in May and September 2020, and tested for differences using chi‐squared statistics. We compared pre‐COVID‐19 breastfeeding and maternity practice data with data during COVID using a fuzzy interrupted time series design with generalized linear models.

**Results:** Pre‐COVID, all Baby‐Friendly documented measures were on a significant continuous upward trend overall and for black dyads in this cohort of hospitals. During COVID, the monthly rate of improvement slowed statistically (1 to 3 percentage points each month) for skin‐to‐skin and rooming practices. No significant changes in trends for breastfeeding initiation or exclusivity were noted. Hospitals used a range of sources as references for changes to maternity care policy, and these policies differed between institutions.

**Conclusions:** COVID‐19 in Mississippi was associated with decreased compliance with the Ten Steps measured here, but there was no corresponding drop in hospital breastfeeding rates, despite hospitals modifying policy to encourage skin‐to‐skin and rooming‐in. Overall, policies and practices were inconsistent during the pandemic, and changes measured to practices were detrimental. Ongoing monitoring is recommended.

## Maternal Services Delivering Breastfeeding or Expressed Breastmilk Support to Social work, Foster Families and Mothers for Babies Removed into Care: A Scoping Review

Vicky Mitchell^1^, Marianne White^2^, Shona Shinwell^1^

^1^Mother and Infant Research Unit, University of Dundee, Dundee, United Kingdom. ^2^NHS Tayside Infant Feeding Team, Dundee, United Kingdom

**Background:** In the UK, a significantly high number of babies are removed from their mothers at birth, and placed into foster care (Raab et al., 2020). Babies in care have the right to be breastfed or receive breastmilk under the UN Convention on the Rights of the Child (United Nations, 2022), however, support to foster families and mothers is often untimely and in cohesive, limiting the success of such practices. By working in partnership, maternal healthcare professionals and social workers can support foster families and mothers with babies in care to continue breastfeeding during contact visits or through milk expressing (Critchley et al., 2021). This review aims to map the literature surrounding support to social workers, families and mothers of babies removed into care, around the topic of breastfeeding or expressed breastmilk (EBM).

**Methods:** The scoping review was conducted across seven electronic databases using synonyms of ‘breastfeeding’, ‘social work', ‘foster care’, ‘infant’, ‘maternal health service’, and ‘support’. The search terms were collated with all researchers and screened with a librarian at the University of Dundee to ensure a thorough search and use of expertise. Grey literature was searched using the phrase ‘guidelines for maternal expressed milk for looked after children’, across Google Scholar and Google. Additionally, forward citation searching was conducted across the included studies. Inclusion criteria was based on the topic of breastfeeding or EBM for babies in care; population included mothers with an infant removed, foster families or immediate care giver, infants under the age of 2 years, social workers, midwives, or infant feeding teams; conducted between the years of 1994 – present; worldwide literature; peer review, case studies, primary and secondary research; both qualitative, quantitative, and mixed method studies with qualitative results being extracted. Data extraction will be conducted by two researchers using data extraction forms. The search has concluded 19 included studies so far, with reference list searching ongoing.

**Conclusion:** This scoping review is part of a wider quality improvement project within NHS Tayside, which aims to improve the services and guidelines for supportive and timely breastfeeding and/or EBM for babies removed into care.

## Postpartum Substance Use Disorder: A Residential Treatment Proposal

Nathan Mittelstadt^1^, Darby Dickton^2^, Caitlan Talamas^3^, Jimi Francis^4^

^1^Texas A&M ‐ San Antonio, San Antonio, USA. ^2^Foundation for Maternal Infant Lactation Knowledge, San Antonio, USA. ^3^UT Health San Antonio, San Antonio, USA. ^4^University of Texas ‐ San Antonio, San Antonio, USA

**Background:** Women with Substance Use Disorder (SUD) are usually underrepresented during their pregnancy due to a lack of prenatal care and are often victims of prejudiced care by providers (Weber et al., 2021). There is also high comorbidity between postpartum depression and SUDs, both appearing to predispose to the other (Chapman & Wu, 2013). Unfortunately, many of these are outpatient techniques that need a great deal of support, both in financial terms and childcare, as they require focus on either mother or the neonate but not usually the mother/infant dyad(Martin & Parlier‐Ahmad, 2021). In this proposed inpatient treatment model, women could attend a residential facility with their infant after delivery.

**Methods:** Care for these mothers would be, ideally, optimized with improved prenatal care before delivery. After delivery, mothers requiring medical observation would complete the mother/neonatal detoxification and then transition to the residential facility for no less than 36 days, at which time their status could be assessed and their stay extended as determined by clinical staff evaluation for no more than 56 days. With active participation and involvement in the program, these mothers will receive a completion certificate from the program for social and/or legal use.

**Results:** Such a facility would provide immersive support to manage the substance disorder under the care of a counseling team; assist with newborn bonding while socially supported by both nursing staff and other inpatient mothers; lastly, lactation consultants would be readily available to optimize infant feeding. Weekly themes would address several critical foundational quality‐of‐life skills, and counseling would use a series of assignments to engage mothers in active reflection of their life and choices. These themes are also aimed at educating residential mothers as parents. Improvements and overall well‐being would be followed weekly with the Maternal Postpartum Quality of Life Instrument (MPQOLI).

**Conclusion:** Although there have, historically, been some trial residential facilities for this population, no current standard for this practice exists (Meinhofer et al., 2020). By having mothers participate in daily support groups, individual counseling sessions, interactive parenting classes, and engaging activities to build healthy hobbies, these women could experience better and sustaining outcomes (Clark, 2001).

**References**

Chapman, S. & Wu, L. (2013). Postpartum Substance Use and Depressive Symptoms: A Review. *Women Health*, 53(5): 479‐503. https://www.ncbi.nlm.nih.gov/pmc/articles/PMC3742364/

Clark, H. (2001). Residential substance abuse treatment for pregnant and postpartum women and their children: treatment and policy implications ‐ PubMed. *Child Welfare*, 179–198. https://pubmed.ncbi.nlm.nih.gov/11291900/

Martin, C. E., & Parlier‐Ahmad, A. B. (2021). Addiction treatment in the postpartum period: an opportunity for evidence‐based personalized medicine. *International Review of Psychiatry (Abingdon, England)*, *33*(6), 579. https://doi.org/10.1080/09540261.2021.1898349

Meinhofer, A., Hinde, J. M., & Ali, M. M. (2020). Substance use disorder treatment services for pregnant and postpartum women in residential and outpatient settings. *Journal of Substance Abuse Treatment*, *110*, 9. https://doi.org/10.1016/J.JSAT.2019.12.005

Weber, A., Miskle, B., Lynch, A., Arndt, S., & Acion, L. (2021). Substance Use in Pregnancy: Identifying Stigma and Improving Care. *Substance Abuse and Rehabilitation*, *12*, 105. https://doi.org/10.2147/SAR.S319180

## The ESSaM Project: An Evaluation of the Safer Sleep for Babies SUDI [Sudden Unexpected Death in Infancy] Prevention Campaign

Laura Murray, Helen Ball, Alice‐Amber Keegan, Megan Law

Durham Infancy & Sleep Centre, Durham University, Durham, United Kingdom

**Background:** In 2018, The Lullaby Trust, UNICEF UK BFI, and Basis (Baby Sleep Info Source) collaborated to design and co‐brand new ‘Safer Sleep for Babies’ (SSfB) materials for use by health professionals and parents across the UK. These materials were endorsed by Public Health England and launched in March 2019. We evaluated the materials and explored how the guidance has been received by health professionals and parents.

**Methods:** Online surveys were conducted with 234 health professionals in 20 NHS Trusts where SSfB had and had not been used. 154 families receiving post‐natal care in the above Trusts also completed surveys, and 13 took part in focus groups.

**Results:** Health professionals who had received SSfB training and who worked in a Trust where the materials were used were more likely to offer parents correct, up‐to‐date safer sleep information. Parents who received SSfB information as part of post‐natal care were more likely to implement safer sleep practices than those who received information from other sources. The SSfB materials encourage dialogue with parents to identify key risks and support families with risk minimisation strategies, particularly with regard to bed‐sharing. This represents a radical departure from previous SUDI/SIDS reduction campaigns that have had a one‐size‐fits‐all approach. Parents who received the intervention were more likely to list co‐sleeping and bed‐sharing as things to consider when thinking about infant sleep safety.

**Conclusions:** The SSfB SUDI Prevention Campaign has been received positively by health professionals and families. Where it has been implemented, it appears to increase the accuracy of safer sleep advice given by health professionals, and the safety of infant sleep practices followed by families.

**References**

Ball, H. L., & Volpe, L. E. (2013). Sudden Infant Death Syndrome (SIDS) risk reduction and infant sleep location ‐ moving the discussion forward. Social Science & Medicine, 79, 84–91. https://doi.org/10.1016/j.socscimed.2012.03.025

Cole, R., Young, J., Kearney, L., Thompson, J. M. D. (2022). Infant Care Practices, Caregiver Awareness of Safe Sleep Advice and Barriers to Implementation: A Scoping Review. Int. J. Environ. Res. Public Health, 19, 7712. https://doi.org/10.3390/ijerph19137712

de Luca, F., Gómez‐Durán, E. L. & Arimany‐Manso, J. (2017). Paediatricians’ Practice About SUDDEN Infant Death Syndrome in Catalonia, Spain. Matern Child Health J, 21, 1267–1276. https://doi.org/10.1007/s10995-016-2225-4

## Exploring blog narratives of parental loneliness: A thematic network analysis

Rebecca Nowland^1^, Gill Thomson^1^, Lucy Cross^1^, Karen Whittaker^1^, Peggy Gregory^1^, Joanna Charles^2^, Crispin Day^3^

^1^University of Central Lancashire, Preston, United Kingdom. ^2^Public Health Waves, Llandudno, United Kingdom. ^3^Kings College London, London, United Kingdom

**Background:** UK‐based national surveys (Action for Children, 2017) and international longitudinal studies (Luoma, Korhonen, Puura, & Salmelin, 2019) have shown that around a third of parents report that they feel lonely often or always. There is limited research about the experience of loneliness in parenthood, however blogs authored by parents, sharing their personal experiences about loneliness offer a potential rich data source (Wilson, Kenny, & Dickson‐Swift, 2015).

**Method:** This study identified and analysed blog narratives written by parents who had experienced loneliness to increase understanding of their experiences. Thematic network analysis (Attride‐Stirling, 2001) was used to analyse the narratives to summarise the main themes, and highlight patterns in understanding by presenting them as a thematic network.

**Findings:** One hundred and ninety‐six relevant blog posts were identified, of which 157 had contact details to request permission to use the blog post in the study. Twenty‐two parent bloggers gave their permission. Four organising themes emerged in the analysis, which centred on a global theme of disconnection. Disconnection underpinned themes relating to a sense that being a parent was overwhelming, changes in identity linked to becoming a parent, difficulties in sharing feelings of loneliness with others, and a lack of social connection.

**Conclusion:** Findings point to parents being unprepared for the transition to parenthood, with implications for antenatal education, and further opportunities for parents to connect to reduce social isolation.

**References**

Action for Children. It starts with hello: a report looking into the impact of loneliness in children, young people and families. London: Action for Children; 2017.

Attride‐Stirling, J. (2001). Thematic networks: an analytic tool for qualitative research. Qualitative research, 1(3), 385‐405.

Luoma, I., Korhonen, M., Puura, K., & Salmelin, R. K. (2019). Maternal loneliness: concurrent and longitudinal associations with depressive symptoms and child adjustment. Psychology, health & medicine, 24(6), 667‐679.

Wilson, E., Kenny, A., & Dickson‐Swift, V. (2015). Using blogs as a qualitative health research tool: a scoping review. International journal of qualitative methods, 14(5), 1609406915618049.

## NICU parents' mental health: A comparative study with parents of term and healthy infants, 1 month after discharge

Christine Persson^1,2^, Jenny Ericson^1,2^, Raziye Salari^3^, Mats Eriksson^4^, Renée Flacking^1^

^1^School of Health and Welfare, Dalarna University, Falun, Sweden. ^2^Centre for Clinical Research Dalarna, Uppsala University, Falun, Sweden. ^3^Child Health and Parenting (CHAP), Department of Public Health and Caring Sciences, Uppsala University, Uppsala, Sweden. ^4^Faculty of Medicine and Health, School of Health Sciences, Örebro University, Örebro, Sweden

**Background:** Parents who have been at neonatal units (NICUs) with their infants are at increased risks for exhibiting symptoms of psychological distress. A stressful birth experience, early and prolonged separation and unclear responsibilities for the infant are identified as key risk factors for the onset of depression, symptoms of psychological trauma, and parental anxiety during the time in NICU (de Paula Eduardo et al., 2019; Flacking et al., 2012). NICU mothers experience lower levels of depression when they have unlimited access to their infants, a trustworthy partnership with staff, receive substantial emotional support (Axelin et al., 2021), and have a single‐family room (Tandberg et al., 2019). In Sweden most parents can stay at the unit 24/7 (Flacking et al., 2019), but we still don't know how NICU parents are affected after discharge when they had the possibility to stay in NICU 24/7 with their infant, compared to parents of term and healthy infants. Aim: To compare mental health in parents of preterm/ill infants and parents of term and healthy infants before birth and 1 month after hospital discharge.

**Method:** A comparative cohort design was used. Parents from six NICUs (n = 439) and four maternal units (MUs) (n = 484) in Sweden answered a survey 1 month after discharge.

**Results:** Parents from NICUs reported significantly more traumatic births and rated their health worse the first week after giving birth, compared to MU parents. One month after discharge, there was no difference between NICU and MU parents regarding symptoms of postnatal depression (EPDS). We observed an association between traumatic birth and depression, but only for MU mothers. Both parents at all NICUs had access to their infant 24/7 and they were significantly more often staying together as a family at the NICU. In total, 80% of NICU parents were satisfied with the emotional support they received from staff and significantly more NICU fathers were satisfied compared to MU fathers.

**Conclusion:** ‘Family togetherness’, parent‐infant closeness, and emotional support at the NICU could be protective factors for developing depression in NICU parents in the short term because it strengthens parenthood, attachment, and resilience.

**References**

Axelin, A., Feeley, N., Cambell‐Yeo, M., Silnes Tandberg, B., Szczapa, T., Wielenga, J., … (SCENE) research group (2021). Symptoms of depression in parents after discharge from NICU associated with family‐centred care. Journal of Advanced Nursing, 00, 1‐12. https://doi.org/10.1111/jan.15128

de Paula Eduardo, J. A. F., de Rezende, M. G., Menezes, P. R., & Del‐Ben, C. M. (2019). Preterm birth as a risk factor for postpartum depression: A systematic review and meta‐analysis. Journal of Affective Disorders, 259, 392‐403. https://doi.org/10.1016/j.jad.2019.08.069

Flacking, R., Breili, C., & Eriksson, M. (2019). Facilities for presence and provision of support to parents and significant others in neonatal units. Acta Paediatrica, 108(12), 2186‐2191. https://doi.org/10.1111/apa.14948

Flacking, R., Lehtonen, L., Thomson, G., Axelin, A., Ahlqvist, S., Moran, V. H., … Dykes, F. (2012). Closeness and separation in neonatal intensive care. Acta Paediatrica, 101(10), 1032‐1037. https://doi.org/10.1111/j.1651-2227.2012.02787.x

Tandberg, B. S., Flacking, R., Markestad, T., Grundt, H., & Moen, A. (2019). Parent psychological wellbeing in a single‐family room versus an open bay neonatal intensive care unit. PloS One, 14(11). https://doi.org/10.1371/journal.pone.0224488

## Complementary Food: A Cohort Study of Nutritional Monitoring in Brazilian Children

Ana Carolina Lavio Rocha^1^, Sophia Izzo^1^, Lucíola Sant'Anna de Castro^1^, Bernardo Lessa Horta^2^, Adriana Souza Torsoni^3^, Mina Desai^4^, Michael Ross^5^, Kelly Pereira Coca^1^

^1^UNIFESP, São Paulo, Brazil. ^2^Universidade Federal de Pelotas, São Paulo, Brazil. ^3^Universidade Estadual de Campinas, São Paulo, Brazil. ^4^The Lundquist Institute, Los Angeles, USA. ^5^University of California, Los Angeles, Los Angeles, USA

**Background:** Childhood obesity is a public health problem and many factors such as maternal obesity, child's birth weight and/or overfeeding are associated with early children being overweight. Adequate food introduction at 6 months age with continuing breastfeeding is related to the prevention of nutritional deviations (Brazil 2019). The aim of this study was to analyze food consumption between 6 and 11 months of infants after exclusive breastfeeding.

**Methods:** We followed exclusively breastfeeding babies who initiate complementary food at 6 months of age in a retrospective cohort study between 2019 and 2020, in an Outpatient Clinic at Breastfeeding Center, São Paulo, Brazil. Data collection was identified by nutritional records of follow up monitored by nutritionists in introduction of complementary food on the sixth and eleventh month of infants. Sociodemographic data, caregivers information, prolonged breastfeeding, and food consumption was analyzed based on the Food Consumption Marker Form of Food and Nutrition Surveillance System based in the last 24 h and the last month (Brazil Ministry of Health). Statistical comparisons were performed with chi‐square and paired and unpaired t‐test.

**Results:** We analyzed 83 infants, 50.6% male, most of them (93,8%) were in exclusive breastfeeding at 6 months, with a significant reduction in breastfeeding to 56.4% at 11 months (*p* < 0.001), as well as the significant increase in formula feeding (*p* = 0.008). Mother was identified as the main caregiver responsible for the care (58%), preparation (53%) and supply of meals (48.8%) to their infants. There was a reduction in the consumption of healthy food markers and an improvement in the adequacy of the consistencies offered in the period (*p* < 0.001), as expected after nutritional follow up. Ultra‐processed foods were introduced into the child's diet from the eleven month, such as instant noodles (23% vs 0%; *p* < 0.001) and sweetened beverages (9.7% vs 1,2%) compared to the 6 months.

**Conclusions:** After 6 months infants need to be monitored to continue supporting breastfeeding and an adequate introduction of complementary food could prevent childhood obesity.

BRASIL (2019). Guia alimentar para crianças brasileiras menores de 2 anos. http://189.28.128.100/dab/docs/portaldab/publicacoes/guia_da_crianca_2019.pdf

## Building a Breastfeeding Friendly Lanarkshire

Susan Short, AnneMarie Bruce

NHS Lanarkshire, Carluke, United Kingdom

**Background:** Breastfeeding rates in Lanarkshire are among the lowest in the UK, historically less than 50% of women start breastfeeding with many stopping quickly so because of this many women in Lanarkshire have never seen a baby being breastfed or see it as an option for them. Despite intensive support to mothers, breastfeeding rates really weren't improving. Unicef Call to Action (2016) resonated with us‐ this wasn't about just supporting the women, it was about the community where they lived.

**Methods:** Using an asset based approach, we have worked for the last 7 years with both colleagues and external partners as well as listening to the communities’ voices to work towards a Breastfeeding Friendly Lanarkshire Approach. We have held an annual “Breastfeeding Summit” multidisciplinary conference to bring together all organisations and parents to see how we can work together to improve breastfeeding in Lanarkshire, identifying barriers as well as what works well.

**Results:** 3 key themes and barriers have emerged to inform action plans and drive the direction of work. These are feeding outside the home, information in pregnancy and education services. The ongoing role out of the Breastfeeding Friendly Scotland scheme, training and resources for staff to support informed discussions, social media campaigns as well as laterally working with partners in education to ensure breastfeeding features in the curriculum from early level to secondary school, has started to show changes to the culture in Lanarkshire, including the drop off (attrition) rates.

**Conclusions:** While we are forever learning and have a long way to go, we continue to evaluate, listen to feedback and working with our communities and families to learn from them what is working and what we can change to create a nurtured and enabled community.

## Infant and Young Child Feeding in Emergencies in the UK

Patricia Wise^1^, Helen Gray^2^, Clare Meynell^2^, Alison Spiro^3^

^1^NCT, Bristol, United Kingdom. ^2^Lactation Consultants of Great Britain, Bath, United Kingdom. ^3^Brunel University, London, United Kingdom

**Background:** Emergencies do occur in the UK and Local Resilience Forums (LRFs) have responsibility for emergency planning in England and Wales, with local authorities having responsibility for accommodation. The World Breastfeeding Trends initiative (WBTi) UK report in 2016 found that there is no UK national guidance on emergency planning for the care and feeding of infants and young children, or of pregnant or breastfeeding mothers.

**Methods:** To supplement the findings of the 2016 WBTi UK report, fresh data was gathered by emailing Local Resilience Fora and local authorities, and by an online search of their websites along with the websites of relevant emergency planning and response organisations in the UK.

**Findings:** There is guidance for citizens on LRF websites about coping in an emergency, usually including the contents of a ‘grab bag’. Supplies needed by families to feed babies during emergencies are rarely laid out in detail. The WBTi UK team held a Forum in 2017 to raise awareness of the issues of infant feeding in emergencies, publishes resources on its blog and website and has developed key social media messages and a Facebook page called “Safely Fed UK: infant and young child feeding in emergencies.” The team also produced a leaflet for LRFs and local authorities in 2022.

**Conclusions:** Supporting breastfeeding as the normal way to feed babies increases their resilience in emergencies, but infants who are not breastfed are more vulnerable. This poster gathers some of the current sources of information for parents but clearer statutory guidance is needed.

**References**

Gray, H. (2018). Breastfeeding in Emergency Situations. In Brown, A. The Positive Breastfeeding Book. Pinter and Martin.

Gray, H. (2020). Infant Feeding in Emergencies: what doctors need to know. In A. Brown and W. Jones W (Eds.). A Guide to Supporting Breastfeeding for the Medical Profession. Routledge.

Infant Feeding in Emergencies Core Group. (2017). Operational Guidance on Infant and Young Child Feeding in Emergencies (3rd ed.). https://www.ennonline.net/operationalguidance-v3-2017

Trickey, H. and Gray, H. (2017). Protecting Babies in Emergencies. DECIPHer blog. Cardiff University. https://decipher.uk.net/blog/remembering-heather-trickey/

WBTi Steering Group. (2016). World Breastfeeding Trends Initiative UK Report 2016. https://ukbreastfeedingtrends.files.wordpress.com/2017/03/wbti-uk-report-2016-part-1-14-2-17.pdf

